# Morphological, Ecological, and Molecular Divergence of *Conogethes pinicolalis* from *C. punctiferalis* (Lepidoptera: Crambidae)

**DOI:** 10.3390/insects12050455

**Published:** 2021-05-15

**Authors:** Na-Ra Jeong, Min-Jee Kim, Sung-Soo Kim, Sei-Woong Choi, Ik-Soo Kim

**Affiliations:** 1 Department of Applied Biology, College of Agriculture and Life Sciences, Chonnam National University, Gwangju 61186, Korea; ioveskfk@naver.com (N.-R.J.); minjeekim3@gmail.com (M.-J.K.); 2 Experiment and Analysis Division, Honam Regional Office, Animal and Plant Quarantine Agency, Gunsan 54096, Korea; 3 Research Institute for East Asian Environment and Biology, Seoul 05207, Korea; nabifri@chol.com; 4 Department of Environmental Education, Mokpo National University, Muan 58554, Korea; choisw@mokpo.ac.kr

**Keywords:** *Conogethes pinicolalis*, *Conogethes punctiferalis*, Pinaceae-feeding type, COI, EF1α

## Abstract

**Simple Summary:**

Currently, two species of *Conogethes* have been documented in Korea: *C. punctiferalis* and *C. pinicolalis*. However, as *C. pinicolalis* has long been considered as a Pinaceae-feeding type of the yellow peach moth, *C. punctiferalis*, studies on *C. pinicolalis* are limited. Therefore, in this study, the divergence of *C. pinicolalis* from the fruit-feeding moth *C. punctiferalis* was analyzed in terms of morphology, ecology, and genetics. *C. pinicolalis* differs from *C. punctiferalis* in several morphological features. Through field observation, we confirmed that pine trees are the host plants for the first generation of *C. pinicolalis* larvae. We successfully reared *C. pinicolalis* larvae to adults by providing them pine needles as a diet. Sequences of mitochondrial *COI* of these two species substantially diverged by an average of 5.46%. Overall nuclear *EF1α*-based phylogeny confirmed each species as an independent clade, but a few haplotypes of *EF1α* indicated incomplete lineage sorting between these two species, suggesting a short divergence time.

**Abstract:**

*Conogethes pinicolalis* has long been considered as a Pinaceae-feeding type of the yellow peach moth, *C. punctiferalis*, in Korea. In this study, the divergence of *C. pinicolalis* from the fruit-feeding moth *C. punctiferalis* was analyzed in terms of morphology, ecology, and genetics. *C. pinicolalis* differs from *C. punctiferalis* in several morphological features. Through field observation, we confirmed that pine trees are the host plants for the first generation of *C. pinicolalis* larvae, in contrast to fruit-feeding *C. punctiferalis* larvae. We successfully reared *C. pinicolalis* larvae to adults by providing them pine needles as a diet. From a genetic perspective, the sequences of mitochondrial *COI* of these two species substantially diverged by an average of 5.46%; moreover, phylogenetic analysis clearly assigned each species to an independent clade. On the other hand, nuclear *EF1α* showed a lower sequence divergence (2.10%) than *COI*. Overall, *EF1α*-based phylogenetic analysis confirmed each species as an independent clade, but a few haplotypes of *EF1α* indicated incomplete lineage sorting between these two species. In conclusion, our results demonstrate that *C. pinicolalis* is an independent species according to general taxonomic criteria; however, analysis of the *EF1α* sequence revealed a short divergence time.

## 1. Introduction

*Conogethes* Meyrick, 1884 (Lepidoptera: Crambidae), is a genus of the subfamily Spilomelinae Guenée, 1854, which includes 15 described species in the Indomalayan and Australasian realm [1,2,3]. The genus contains several species that are considered pests on economically important food plants and pine trees including *Conogethes punctiferalis* Inoue et Yamanaka, 2006 (Lepidoptera: Crambidae). *C. punctiferalis* is distributed widely in Asia and Oceania, and especially in Korea, Japan, China, Taiwan, Vietnam, Myanmar, Thailand, Nepal, India, the Philippines, and Australia [4]. *C. punctiferalis* can be recognized from its light yellow or orange scales distributed throughout the body and black spots scattered on the wings and dorsal abdomen [4]. Larvae of *C. punctiferalis* are a serious pest of chestnut, apple, pear, plum, and papaya [5,6]. This species reproduces twice per year in Korea but often produces three generations per year in the southern region [7]. In the first generation, adults of *C. punctiferalis* lay eggs on fruits, such as peaches and plums; next, the larvae damage the fruits [8], and during the second generation, adults of *C. punctiferalis* lay eggs on chestnuts [9,10].

In Japan, *C. punctiferalis, C. pinicolalis*, and *C. parvipunctalis* have been recorded [4], but the species status of *C. pinicolalis* has long been argued. *C. punctiferalis* has been acknowledged to consist of two ecotypes, which differ in their host plant preference, namely a fruit-feeding type (FFT) and a Pinaceae-feeding type (PFT) [11]. The PFT is oligophagous, feeding mainly on various species of pines (Pinaceae) in Japan [4], but not on fruits, in contrast to the polyphagous FFT [4,12]. However, males of the two ecotypes were interchangeably attracted to both female ecotypes, owing to a similar female sex pheromone system [13]. Later, Honda and Mitsuhashi [14] found differences between the FFT and PFT, with respect to morphological characteristics, such as male genitalia, the ovipositor in female genitalia, as well as the larval mouth, larval pinacular, and pupal cremaster. However, these authors did not classify the FFT and PFT into different species because of the interchangeable attractancy discovered in a previous study [15]. In contrast, Inoue and Yamanaka [4] described the PFT as a new species, *C. pinicolalis*, separated from the FFT, *C. punctiferalis*, mainly based on the morphological features.

In Korea, the genus *Conogethes* has been listed as including a single species, *C. punctiferalis* [16]; however, Inoue and Yamanaka [4] recorded *C. pinicolalis*, formerly known as the PFT of *C. punctiferalis*, in Korea, using a total of 97 specimens collected in Japan, Korea, Taiwan, China, and Thailand. According to the Illustrated Flora and Fauna of Korea [17], the PFT was once recorded as *Dichocrosis* sp., whose genus name was later synonymized with *Conogethes* [18], but no species name was assigned to it. Nevertheless, no subsequent study has assessed the species status of *C. pinicolalis* in Korea; thus, here *C. pinicolalis* has long been considered a PFT of *C. punctiferalis*. Furthermore, this species is not listed in the checklist of Korean insects [16], and consequently, no information on ecological aspects of *C. pinicolalis*, including its preferential hosts, is available in Korea.

To examine the species status of *C. pinicolalis*, formerly known as the PFT of *C. punctiferalis*, its divergence from *C. punctiferalis* was analyzed from a morphological, ecological, and genetic perspective. For the purpose of this study, first, *C. pinicolalis* larvae dwelling on the leaves of pine trees were monitored in the field, and adults were collected using either insect nets or pheromone traps installed with a lure for *C. punctiferalis*. Second, field-collected *C. pinicolalis* larvae at various stages were cultured indoors on pine tree leaves until egg deposition. Third, adult morphology was compared between the two species. Finally, the sequence divergence of geographic samples of the two species was analyzed by applying phylogenetic and population genetic analyses to the mitochondrial cytochrome c oxidase subunit I (*COI*) and nuclear elongation factor 1 alpha (*EF1α*).

## 2. Materials and Methods

### 2.1. Sampling

**Figure 1:**
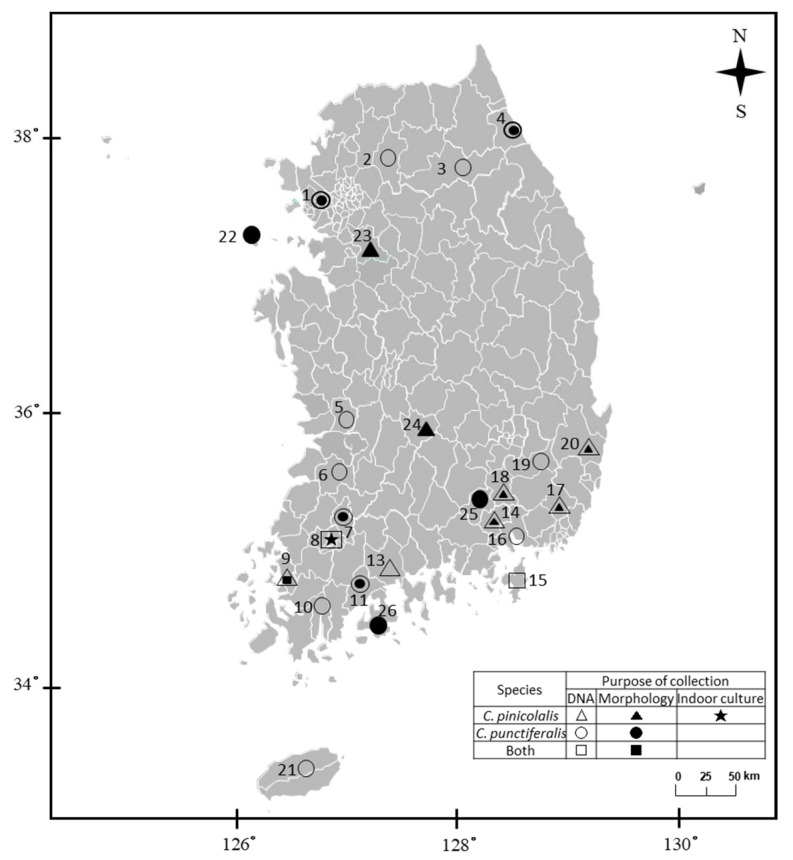
Sampling locations of *Conogethes pinicolalis* and *C. punctiferalis* in Korea. The general locality names are as follows: 1, Mt. Gyeyangsan, Incheon Metropolitan City; 2, Gapyeong, Gyeonggi Province; 3, Hongcheon, Gangwondo Province; 4, Yangyang, Gangwondo Province; 5, Iksan, Jeollabukdo Province; 6, Jeongup, Jeollabukdo Province; 7, Damyang, Jeollanamdo Province; 8, Gwangju Metropolitan City; 9, Mt. Seungdalsan, Muan, Jeollanamdo Province; 10, Gangjin, Jeollanamdo Province; 11, Boseong, Jeollanamdo Province; 12, Suncheon, Jeollanamdo Province; 13, Sancheong, Gyeongsangnamdo Province; 14, Mt. Jayangsan, Haman, Gyeongsangnamdo Province; 15, Geoje, Gyeongsangnamdo Province; 16, Changwon, Gyeongsangnamdo Province; 17, Mt. Wonhyosan, Yangsan, Gyeongsangnamdo Province; 18, Mt. Hwawangsan, Changnyeong, Gyeongsangnamdo Province; 19, Cheongdo, Gyeongsangbukdo Province; 20, Gyeongju, Gyeongsangbukdo Province; 21, Jeju, Jejudo Province; 22, Gulupdo, Incheon Metropolitan City; 23, Yongin, Gyeonggido Province; 24, Muju, Jeollabukdo Province; 25, Ueiryeong, Gyeongsangbukdo Province; and 26, Goheung, Jeollanamdo Province. △, ○, and □ indicate the localities where individuals of *C. pinicolalis*, *C. punctiferalis*, and both species used in the molecular experiment were collected, respectively. ▲, ●, and ■ indicate the localities where individuals of *C. pinicolalis*, *C. punctiferalis*, and both species used for morphological trait comparison were collected, respectively. ★ indicates the localities where *C. pinicolalis* individuals were observed for ecological analysis and collected for indoor culture.

Every 10 days from 12 April to 2 July 2019, Korean red pines of the species *Pinus densiflora* (varieties *P. densiflora* ‘Aurea’ and *P. densiflora* f. *multicaulis* Uyeki) were monitored for the presence of *C. pinicolalis* at a plantation located in the city of Gwangju (locality 8; Figure 1) and an arboretum located in Suncheon, Jeollnamdo Province (locality 12; Figure 1), Korea, where pine trees and pine seedlings, respectively, are grown for commercial purposes; here, larvae were hand collected for indoor ecological observation and culture. On 13 and 27 June 2020, respectively, a single and two adult males, which were observed on Korean red pines during field monitoring, were also hand collected. These adults collected from red pines and some of the larvae were used for sequence analysis after morphological record (Figure 1).

For morphological examination, a total of 11 adults of *C. pinicolalis* and 14 adults of *C. punctiferalis* were collected from eight and nine localities in Korea, respectively (Figure 1). The wing expanse, labial palpus, male hind tibia, and genitalia of both species, which are important morphological characteristics in *Conogethes*, were examined.

For molecular analysis, 59 and 98 individuals of *C. pinicolalis* and *C. punctiferalis*, respectively, among adults and larvae, were collected from nine and 15 localities in Korea between 20 June 2012 and 20 April 2019 (Figure 1; Table S1). The adults of both species used for morphological examination and molecular analysis were collected using either light traps or a *C. punctiferalis*-specific pheromone lure installed in funnel traps (Greenagrotech, Gyeongsan, Korea). *C. punctiferalis* larvae at various stages were all collected from the inside of fallen fruits, such as peaches (*Prunus persica*), apricots (*Prunus armeniaca*), and chestnuts (*Castanea crenata*), whereas *C. pinicolalis* larvae were collected from Korean pine trees. Field-collected individuals were stored at −70 °C for subsequent molecular experiments. Physical voucher specimens of each species (adults or larvae) were deposited in Chonnam National University and Mokpo National University.

### 2.2. Insect Rearing

To investigate the life cycle of *C. pinicolalis*, field-collected *C. pinicolalis* larvae at various stages were brought to the laboratory. For indoor culture, *C. pinicolalis* larvae were placed in an insect breeding dish (diameter, 10 cm; height, 4 cm; SPL Life Science, Pocheon, Korea) and provided with fresh pine branches collected in the field on a filter paper moistened with double distilled water (Figure S1A). Fresh pine needles were provided to the larvae whenever one half of the pine needles had been consumed. After emergence from pupal cocoons, adults were allowed to mate at a 1:1 or 2:1 of male-to-female ratio in the mating space, which was shaped into a triangular pyramid with sides of approximately 17 cm made of butter paper (Figure S1B). Alternatively, two differently sized insect breeding cages (20 × 20 × 15 cm, Figure S1C; or 24.5 × 24.5 × 63 cm, Figure S1D) were provided for mating [15]. To induce egg deposition, a gauze-covered pack containing pine needles and pine powder was installed in the insect breeding cages (Figure S1E) [19]. Mating and oviposition were monitored every 24 h.

Immediately after oviposition, eggs were observed but were crushed one day after oviposition as they did not hatch. Therefore, the full life cycle starting from the egg stage was not successfully monitored. Nevertheless, pupal duration, the genital morphology of both sexes, the life span of adults, and the shape of eggs were investigated because after collection from the field, most larvae at various stages survived until the adult stage and some of the females produced eggs.

### 2.3. Genomic DNA Extraction

Genomic DNA was extracted from two hind legs of adults and from the thorax and abdomen of larvae after removal of midgut and head using a Wizard^TM^ Genomic DNA Purification Kit (Promega, Madison, WI, USA), proteinase K (Thermo Fisher Scientific, Rockford, IL, USA), isopropyl alcohol (Amresco, Solon, OH, USA), and 70% ethanol.

### 2.4. PCR and Sequencing

Referring to a preceding study [20], forward (5′-ACTCAACAAATCATAAAGATATTGG-3′) and reverse (5′-TGATTTTTTGGTCACCCTGAAGTTTA-3′) primers targeting *COI* were designed to amplify and sequence a 658-bp region of *COI*, corresponding to the DNA barcoding region. PCR was conducted under the following conditions: an initial denaturation at 94 °C for 4 min, followed by 30 cycles of 94 °C for 1 min, 50–51 °C for 1 min, and 72 °C for 1 min, with a subsequent final extension at 72 °C for 7 min.

The *EF1α* primers used in this study were adapted from previous studies: the forward directional primer (Oscar-6143, 5′-GGCCCAAGGAAATGGGCAAGGG-3′) from Hundsdöerfer et al. [21] and the reverse directional primer (EfrcM4, 5′-ACAGCVACKGTYTGYCTCATRTC-3′) from Monteiro and Pierce [22]. After sequencing a few individuals using this primer set, an additional pair of primers to amplify approximately 787 bp of *EF1α*, excluding the primer sites, were designed to increase amplification efficiency: forward directional primer, 5′-AAATATGCCTGGGTATTGGAC-3′; reverse directional primer, 5′-CTTGGAGTCTCCAGCGACGT-3′. Thirty-five cycles of amplification (94 °C for 1 min, 50–56 °C for 1 min, and 72 °C for 1 min) were conducted after an initial denaturation step at 94 °C for 4 min, and the final extension step was performed for 10 min at 72 °C. *COI* amplicons were directly sequenced after PCR and purification using a PCR Purification kit (Qiagen, Hilden, Germany), whereas *EF1α* amplicons were cloned after PCR. Cloning was carried out using a T-Blunt^TM^ PCR Cloning kit (SolGent, Daejeon, Korea) and HIT^TM^ DH5α High 10^8^ competent cells (Real Biotech Co., Banqiao City, Taiwan). The resultant plasmid DNA was isolated using a Plasmid Mini Extraction Kit (Bioneer, Daejeon, Korea). Electrophoresis was carried out to confirm successful DNA amplification using 0.5× TAE buffer on a 0.5% agarose gel. DNA sequencing was conducted using the ABI PRISM^®^ BigDye^®^ Terminator ver. 3.1 Cycle Sequencing kit with an ABI PRISM^®^ 3100 Genetic Analyzer (Applied Biosystems, Foster City, CA, USA). All products were sequenced from both strands.

### 2.5. Sequence Analysis

*COI* was sequenced for each of the 59 *C. pinicolalis* and 98 *C. punctiferalis* individuals collected from nine and 15 localities, respectively (Figure 1; Table S1). For *EF1α*, 4–13 clones per individual were sequenced for 12 *C. pinicolalis* individuals collected from eight localities and 5–14 clones per individual were sequenced for 15 *C. punctiferalis* individuals collected from nine localities (Figure 1; Table S1).

Both directional *COI* and *EF1α* sequences of each individual were aligned using SeqMan (DNASTAR, Madison, WI, USA) to generate qualified individual sequences. Sequence alignment was conducted using Clustal Omega [23]. Each *COI* and *EF1α* sequence was compared to those available in public sequence databases, such as GenBank, through a Blast search (http://blast.ncbi.nlm.nih.gov/Blast.cgi, accessed on 15 January 2021) to verify the accuracy of the sequences. Each *COI* and *EF1α* sequence was considered a different haplotype when homologous sequences from two individuals differed by ≥1 nucleotide for *COI* and either differed by ≥1 nucleotide or presented an insertion/deletion for *EF1α*. Thus, haplotypes were designated as follows: BARCPI01, BARCPI02, BARCPI03, and so forth for *C. pinicolalis COI*; BARCPU01, BARCPU02, BARCPU03, and so forth for *C. punctiferalis COI*; EF1ACPI01, EF1ACPI02, EF1ACPI03, and so forth for *C. pinicolalis EF1α*; EF1ACPU01, EF1ACPU02, EF1ACPU03, and so forth for *C. punctiferalis EF1α*. Among *EF1α* sequences, several untranslated pseudogene sequences were detected. These were named PI1, PI2, PI3, and so forth for *C. pinicolalis* and PU1, PU2, PU3, and so forth for *C. punctiferalis* (Table S1). The sequence divergences of each *COI* and *EF1α* haplotype were calculated via the unrooted pairwise distance method using PAUP* ver. 4.0a167 [24].

### 2.6. Phylogenetic Analysis

For phylogenetic analysis to illustrate the relationship between *C. pinicolalis* and *C. punctiferalis*, 81 *COI* sequences from nine *Conogethes* species, including 35 *C. punctiferalis COI* haplotypes that originated from Korea, China, Pakistan, Australia, Japan, and Thailand were retrieved from GenBank and BOLD Systems (Table S2). These sequences overlap with ≥655 bp of current *COI* sequences. However, no single sequence of *C. pinicolalis COI* corresponds to this length. For *EF1α*-based phylogenetic analysis, only sequences obtained in this study were used because only the *EF1α* sequence of *Conogethes* nr. *punctiferalis* (GenBank acc. no. JX017872) was recorded in GenBank. Sequence alignment was performed using Clustal X software [25]. The GTR + GAMMA + I model was selected as the best substitution model for both *COI* and *EF1α* by Modeltest and was applied for carrying out Bayesian inference (BI) using MrBayes ver. 3.2.7 [26], which is incorporated into the CIPRES Portal ver. 3.1 [27]. Two independent runs of four incrementally heated Monte Carlo-Monte Carlo chains (one cold chain and three hot chains) were simultaneously run for one million generations, with sampling conducted every 100 generations. The confidence values of the BI tree are presented as Bayesian posterior probabilities (BPPs) in percent. The co-familial species *Glyphodes quadrimaculalis* [28] and *Eurrhyparodes* cf. *lygdamis* [29] were used as an outgroup for *COI* and *EF1α*, respectively. Trees were visualized with FigTree ver. 1.4.4 (http://tree.bio.ed.ac.uk/software/figtree/, accessed on 15 January 2021).

### 2.7. Population Genetic Structure

Principal coordinate analysis (PCoA) [30] was performed using the pairwise genetic distance (*F*_ST_), which was obtained from Arlequin ver. 3.5 [31], to detect and plot the relationships among *C. punctiferalis* and *C. pinicolalis* populations using GenAlEx ver. 6.5 with default parameters [32]. The genetic structure of *C. punctiferalis* and *C. pinicolalis* populations was analyzed using Bayesian Analysis of Population Structure (BAPS) ver. 6.0 [33]. The analysis was performed by clustering, with a linked locus option and an independent model. In this process, mixture analysis was performed to identify optimal clusters based on maximum log marginal likelihood values (*K*), which ranged from 1 to 10.

## 3. Results

### 3.1. Biology

**Figure 2:**
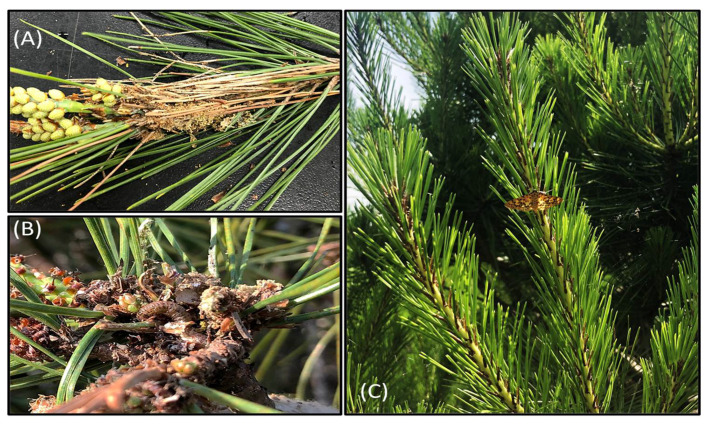
Representative images of *Conogethes pinicolalis* individuals observed on pine trees. (**A**) *C. pinicolalis* cocoon; (**B**) *C. pinicolalis* larvae; (**C**) *C. pinicolalis* adult.

During field monitoring, we found damaged pine needles, which were stuck together to form bundles (Figure 2A). Inside bundles, larvae were living in lengthy semi-blocked cocoons made of pine needles and ball-shaped excrement, the interior of which was lined with white silk (Figure 2A,B). These observations confirmed that the pine tree is indeed the host plant for *C. pinicolalis* larvae. After the first observation of a *C. pinicolalis* adult male on a pine tree on 13 June (Figure 2C), two adults and four empty pupae exuviae were additionally observed on 27 June 2019, confirming that the pine tree is the host plant for *C. pinicolalis*.

**Figure 3:**
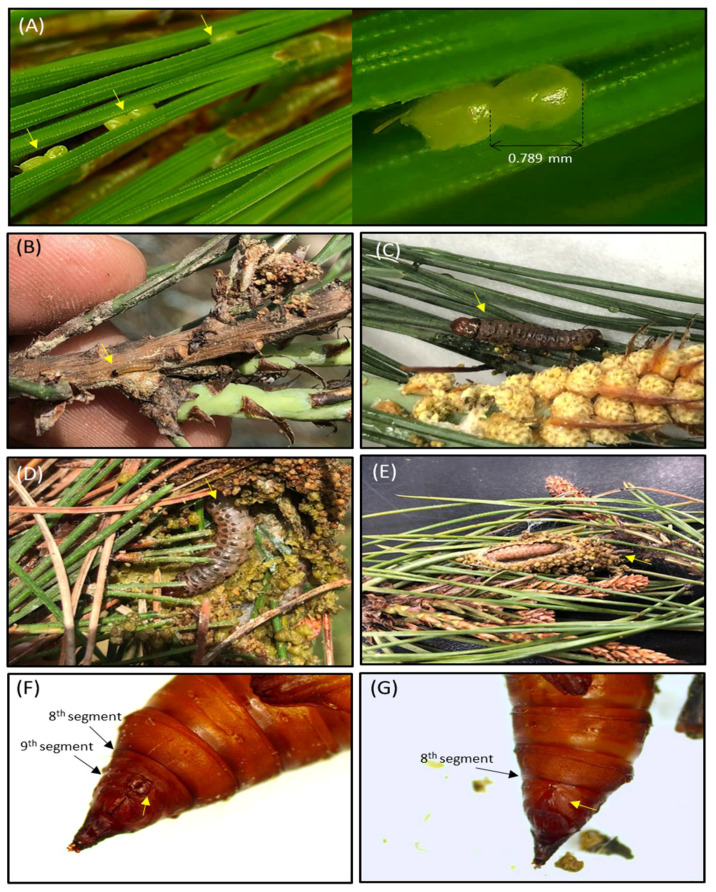
Developmental stages of *Conogethes pinicolalis* larvae. (**A**) Eggs on pine needles. (**B**) Young larvae; (**C**) Mid-stage larvae; (**D**) Last-stage larvae; (**E**) Pre-pupal-stage larvae. (**F**) Male pupa, having two bumps separated by a narrow groove that resembles the Arabic numeral eight (8) on the abdominal segment 9; (**G**) female pupa, exhibiting a longitudinal notch at the genitalia opening on the abdominal segment 8.

To investigate the life cycle of *C. pinicolalis*, field-collected larvae, along with pine branches, were brought to the laboratory. *C. pinicolalis* larvae at various instars were provided with pine needles and branches (Figure S1). Larvae actively ate new pine shoot and successfully pupated and emerged. To obtain fertilized eggs, the adults were allowed to mate at a 1:1 or 2:1 of male-to-female ratio, while placed in three different conditions, specifically in a triangular pyramid (Figure S1B) or two differently sized insect breeding cages (Figure S1C,D), and provided with a gauze-covered pack containing pine needles and pine powder. Although no egg was laid in the triangular pyramid and smaller-sized cage by any of the 16 tested adult pairs, eight *C. pinicolalis* females placed in the larger insect breeding cage deposited 22 eggs; however, eggs did not hatch. Eggs were yellowy, oval, with a maximum diameter of approximately 0.79 mm and were deposited alone or in pairs between pine needles (Figure 3A). As no egg hatched, we were unable to measure the duration of the egg and larval stages. It is not possible to infer the exact reason why the eggs did not hatch, but one likely hypothesis is that they failed to get fertilized as the copulation activity was very limited. Additional studies are required to evaluate this hypothesis. Nevertheless, larvae at different stages were successfully grown into pupae and adults.

During larval culture of *C. pinicolalis*, approximately three successive exuviae were observed; therefore, we speculated that larvae grew until at least the 4th instar. Based on this observation, larvae were divided into three categories showing different morphological characteristics, as follows: young larvae (i.e., 1st−2nd instar), with a yellowish-black head (Figure 3B); mid-stage larvae (i.e., 3rd instar), with a dark body color and a brown head (Figure 3C); last-stage larvae (i.e., 4th instar), with a bright green and pale pink body (Figure 3D). Pre-pupal-stage larvae stopped eating and lived in a blocked cocoon shaped as an elongated oval, made of pine needles and ball-shaped excrement, and lined in white silk at the interior, similar to those observed in the field (Figure 3E). After pupation in the cocoon, pupae underwent sclerotization and became brown. Male pupae had two bumps that were separated by a narrow groove resembling the Arabic numeral eight (8) on the abdominal segment 9 (Figure 3F), whereas female pupae had a longitudinal notch at the genitalia opening on the abdominal segment 8 (Figure 3G). The pupal and adult period lasted approximately 12.02 and 8.05 days, respectively (Table S3).

### 3.2. Systematics

**Order Lepidoptera Linnaeus, 1758**

**Family Crambidae Latreille, 1810**

**Genus *Conogethes* Meyrick, 1884**

*Conogethes* Meyrick, 1884, Trans. Ent. Soc. 1884:314.

**Figure 4:**
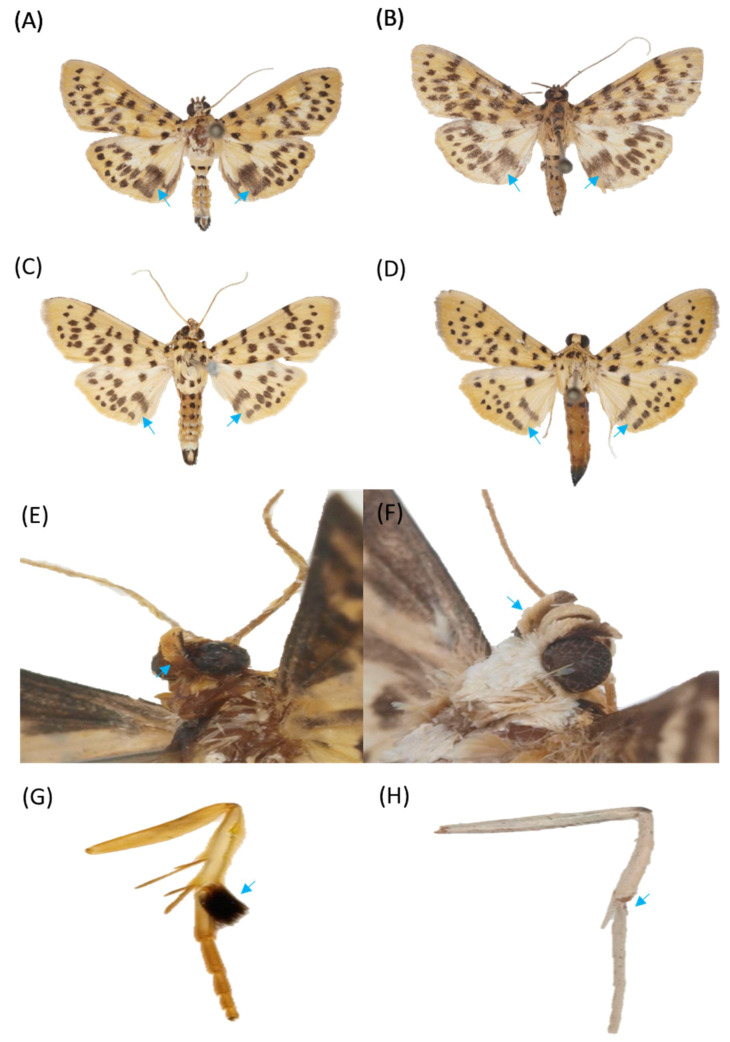
Representative images of *Conogethes pinicolalis* and *C. punctiferalis* adults. (**A**) *C. pinicolalis* male; (**B**) *C. pinicolalis* female; (**C**) *C. punctiferalis* male; (**D**) *C. punctiferalis* female. (**E**) Lateral view of the labial palpus of *C. pinicolalis*; (**F**) Lateral view of the labial palpus of *C. punctiferalis*. (**G**) Hind tibia and hind tarsus of a *C. pinicolalis* male; (**H**) Hind tibia and hind tarsus of a *C. punctiferalis* male. Arrows on adult hind wings indicate scattered large black spots at the hindwing. Arrows on the hind tibia indicate a large tuft on the first segment of the hind tibia.

**Figure 5:**
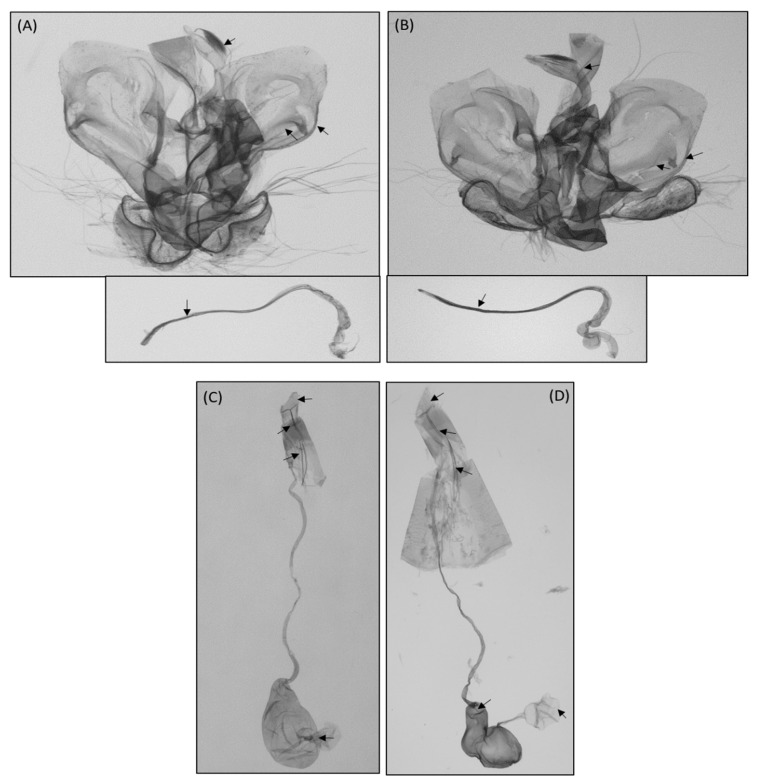
Male and female genitalia of *Conogethes pinicolalis* and *C. punctiferalis*. (**A**) *C. pinicolalis* male; (**B**) *C. punctiferalis* male; (**C**) C. pinicolalis female; (**D**) *C. punctiferalis* female.

***Conogethes pinicolalis* Inoue and Yamanaka, 2006**

(Korean name: So-na-mu-deul-myeong-na-bang) Figs. 4A, 4B, 4E, 4G, 5A, and 5C (in this study)

*Conogethes pinicolalis* Inoue and Yamanaka, 2006, Tinea 19 (2): 80.

Type locality: Bushi Iruma City Honshu Pref., Japan.

*Conogethes* sp.: Inoue, 1982, 1: 338, pl. 39, figs. 36, 37.

*Dichocrocis* sp.: Park, 1983: 340, pl. 20, Figure 309.

*Astura punctiferalis* (part): Pryer, 1885, Trans. Asiat. Soc. Japan 13: 63. nec Guenée, 1854.

Material examined. One female, Yongin, Gyeonggido Province, 37°17′33.3” N 127°09′36.0” E, 25 June 2017, MNU 3744, Kim SS; one female, Muju, Jeollabukdo Province, 23 September 2016, MNU 3745, Kim SS; two males, two females, Suncheon, Jeollanamdo Province, 35°04′51.3” N 127°22′02.2” E, 8 June 2018, CNU 8196, 8198, 8197, 8199, Kim I; one male, Mt. Seungdalsan, Jeollanamdo Province, 34°54′31.7” N 126°27′24.7” E, 31 August 2016, MNU 3746, Kim SS; one male, Mt. Jayangsan, Gyeongsangnamdo Province, 35°17′26.7” N 128°26′55.7” E, 6 October 2016, CNU 8484, Kim SS; one male, Mt. Wonhyosan, Gyeongsangnamdo Province, 35°23′53.3” N 129°06′19.0” E, 30 June 2016, CNU 8485, Kim SS; one female, Mt. Hwawangsan, Gyeongsangnamdo Province, 26 June 2014, MNU 3732, Kim SS; one female, Gyeongju, Gyeongsangbukdo Province, 2 September 2009, MNU 3747, Kim SS. Abbreviations are as follows: MNU, Mokpo National University; CNU, Chonnam National University.

Diagnosis. This species is slightly smaller than *C. punctiferalis*, with an average wingspan of 2.531 mm (range, 2.240–2.891 mm; n = 11). The wing ground color and pattern elements are very similar to those of *C. punctiferalis*, but *C. pinicolalis* can be distinguished by the dark ochreous second segment of the labial palpus, which is yellowish white in *C. punctiferalis*. A distinctive feature of male adults is a large fuscous tuft on the hind tibia, which has no tuft in *C. punctiferalis*. Moreover, the series of black spots on the upper sides of both wings are usually larger than those of *C. punctiferalis*, especially the postmedial series on the hind wings, which usually coalesce. The male genitalia of *C. pinicolalis* are similar to those of *C. punctiferalis,* but can be distinguished by a more protruded distal margin of the valva, a thick saccular arm, and a long aedeagus. Conversely, the female genitalia of *C. pinicolalis* are very similar to those of *C. punctiferalis* and almost impossible to distinguish from them.

Hosts. Pinus densiflora, P. thunbergii, P. koraiensis, P. parviflora, P. rigida, Abies holophylla, A. koreana, Cedrus deodara, C. atlantica, and C. libani in the Pinaceae [4].

Distribution. Korea [Gwangju, Muan, Suncheon, Geoje, Haman, Yangsan, Sancheong, Changnyeong, Gyeongju (this study); Mt. Nojasan, Geoje (Bae et al. [34]); Mt. Jirisan, Sancheong (Department of Biology, University of Incheon); Mt. Sambangsan, Yeongweol (Bae et al. [34]); Mueui, Incheon (Kim et al.)], Japan [Hokkaido- Kuroiwa, Yakumocho (Kogi H); Tomarikawa, Kumaishicho (Kogi H); Kaitorima, Taisei-cho, Kudou-gun (Komatsu T); Ukishima Park, Kitahiyama Town, Setana-gun (Komatsu T); Okawa, Nanae Town, Kameda-gun (Komatsu T); Honshu-Takao-san, Tokyo (Yamanaka H); Kugenuma, Kanagawa (Inoue H); Toshiya, Unazuki Town, Toyama (Tanaka C); Azohara, Unazuki Town, Toyama (Yamanaka H); Kokurobe, Unazuki Town, Toyama (Yamanaka H); Kanetsuri, Unazuki Town, Toyama (Yamanaka H) Eiraku-sho, Toyama City, Toyama (Yamanaka H); Seyo-machi, Fukuyama City, Hiroshima (Tomisawa A); Ryukyu-Uragami, Naze, Amami-oshima (Sekiguchi Y)], Taiwan [Paleng, Taoyuan Hsien (Shibata Y)], China [Nanling, Shaoguan, Guangdong (Kishida Y and Sato R); Nankunshan, Huizhou, Guangdong (Kishida Y and Sato R)] and Thailand [Doi Pui, Chiang Mai (Owada M)] [4].

**Conogethes punctiferalis Guenée, 1854**

(Korean name: Bok-sung-a-myeong-na-bang) Figure 4C,D,F,H, and Figure 5B,D

*Astura punctiferalis* Guenée, 1854, in Boisduval and Guenée, Hist. nat. Insects (Lépid.) 8: 320. Type locality: Central India.

*Dichocrocis punctiferalis*: Park, 1983: 339, pl. 20, Figure 308.

*Conogethes punctiferalis*: Bae, Byun, and Paek, 2008: 94.

Material examined. One male, Mt. Gyeyangsan, Incheon Metropolitan City, 37°33′16.4” N 126°42′54.2” E, 12 October 2018, CNU 8214, Kim I; one male, Gulupdo, Incheon Metropolitan City, 29 August 2016, MNU 3308, Kim SS; one male, Yangyang, Gangwondo, Province, 24 September 2018, CNU8218, Kim I; one male, Ueiryeong, Gyeongsangbukdo Province, 1 June 2014, MNU 3733, Kim SS; one male, two females, Boseong, Jeollanamdo Province, 34°51′56.8” N 127°18′36.8” E, 28 June 2018, CNU 8166, 8167, 8169, Kim I; one male, Suncheon, Jeollanamdo Province, 34°58′22.4” N 127°14′46.6” E, 3 October 2018, CNU 8228, Kim I; two females, Suncheon, Jeollanamdo Province, 35°04′12.8” N 127°13′39.5” E, 28 September 2018, CNU 8232, 8234, Kim I; one male, Damyang, Jeollanamdo Province, 35°11′14.2” N 126°58′47.8” E, 15 October 2018, CNU 8238, Kim I; one male, one female, Muan, Jeollanamdo Province, 19 August 2018, MNU 3732, 3734, Kim SS; one female, Goheung, Jeollanamdo Province, 22 August 2014, MNU 3748, Kim SS.

Diagnosis. The average wingspan of *C. punctiferalis* is of approximately 2.804 mm (range, 2.636–2.997 mm; n = 14). The labial palpus is broad, upturned, and pale yellow, while the second segment of the labial palpus is mostly yellowish white. The frons are pale yellow and evenly scaled, and antennae are pale brownish-yellow and filiform in both sexes. The wing ground color ranges from pale yellow to orange yellow; the forewing base has several areas showing four black spots; the hindwings consist of a series of black spots on the antemedial, postmedial, and submarginal lines, with a rather large discocellular black spot. The abdomen is pale yellow dorsally with black spots on each segment and a black anal tuft in males. The male genitalia of *C. punctiferalis* are characterized by a narrow, slender, and curved ventrad; an uncus dilated in its apical one third; a basally expanded juxta; a short and more or less tapered saccus; a short and slightly oval valva with a narrow and tapered sacculus and a weakly protruded saccular margin; and a very long, slender, and basally strongly curved aedeagus with a long and slender thorn-like cornutus. The female genitalia of *C. punctiferalis* have an apophysis anterioris about as long as the apophysis posteriors; a narrow, membranous, and funnel-shaped ostium; a sclerotized antrum; a narrow and long doctus bursae; and an ovate corpus bursae with an appendix bursae.

Hosts. Quercus acutissima and Castanea crenata in the Fagaceae; Prunus serrulata var. spontanea, P. persica, P. armeniaca, Malus pumila, and Pyrus pyrifolia var. culta in the Rosaeae; Ficus carica in the Moraceae; Diospyros kaki in the Ebenaceae; Citrus sinensis in the Rutaceae; Gossypium hirsutum in the Malvaceae; Punica granatum in the Lythraceae; and Helianthus annuus in the Asteraceae [35,36]. The larvae of this species have been known mainly as a pest of fruits and pods of many plants in the Eastern Palearctic and Indo-Australian regions [4,8,37].

Distribution. Korea [Incheon, Gapyeong, Hongcheon, Yangyang, Iksan, Jeongup, Damyang, Gwangju, Suncheon, Boseong, Gangjin, Cheongdo, Changwon, Geoje, Jeju-Island (this study); Changdeokgung, Seoul (Bae et al. [34]); Park Incheon, Incheon (Lee CM); Mt. Gwanmosan, Incheon (Bae et al. [34]); Mt. Geomdansan, Hanam (Lee et al.); Mt. Gamaksan, Yeoncheon (Bae et al. [34]); Byeonsanbando, Buan (Bae et al. [34])], Japan [Hokkaido-Yoshioka-toge, Fukushima Town, Matsumae-gun (Komatsu T); Honshu-Takao-san, Tokyo (Inoue H and Yamanaka H); Chigasaki, Kanagawa (Inoue H); Kugenuma, Kanagawa (Inoue H); Bushi, Iruma City, Saitama (Inoue H); Mt. Iwamuro, Shiquoka (Inoue H); Nakabusa Spa, Minamiazumi-gun, Nagano (Yamanaka H); Mt. Mitsubo, Asahi-machi, Toyama (Yamanaka H); Inonedaira, Arimine, Toyama (Yamanaka H); Osawano Town, Toyama (Yamanaka H); Ioridani, Hosoiri-mura, Toyama (Nakai A); Sannokuma, Furudoike, Toyama City, Toyama (Yamanaka H); Kanetsuri, Unazuki Town, Toyama (Yamanaka H); Toha-mura, Toyama (Yamanaka H); Kurikara, Tsubata-machi, Ishikawa (Tomisawa A); Houdatsusan, Oshimizu-machi, Ishikawa (Tomisawa A); Shishiku-kogen, Tsurugi Town, Ishikawa (Tomisawa A); Saikakurindo, Tsurugi Town, Ishikawa (Tomisawa A); Ishinose, Shiramine-mura, Ishikawa (Tomisawa A); Shikoku-Uchiko. Kita-gun, Ehime (Yasukawa M); Kyushu-Ikeda, Kimotsuki-gun, Kagoshima (Yasukawa M); Uchinono, Hioki-gun, Kagoshima (Yasukawa M); Izuhara, Tsuhima (Yasukawa M); Nenbutsuzaka, Tsushima (Watanabe T); Konogiyama, Tshshima (Yatanabe T); Mitake, Tsushima (Yatanabe T); Azamo, Tsushima (Watanabe T); Konogiyama, Tsushima (Watanabe T); Shirikubiyama, Tsushima (Watanabe T); Taterayama, Tsushima (Watanabe T); Ryukyu-Nakijin, Okinawa (Azuma S); Yona, Okinawa (Owada M and Deguchi K); Ishigakjima (Kanmiya K); Komi, Iriomotejima (Owada M); Funaura, Iriomotejima (Azuma S and Kanazawa I)], Taiwan [Hernglong Lodge, Miaoli Hsien (Kawabe A)], China [Nanling, Shaoguan, Guangdong (Sata R and Kishida Y); Nankunshan, Huizhou, Guangdong (Sata R)], Vietnam [Bao Loe, 18 km from Ho Chi Minh (Endo T)], Myanmar, Thailand [Lamphun (Kuroki et al.); Doi Pui, Chiang Mai (Kuroki et al.); Khao Yai, Nakhon Nayok (Kuroki et al.); Doi Inthanon, Chiyanh Mai (Owada M, Kuroki et al.); Doi suthep, Chiang Mai (Saito S and Saito A)], Nepal [Godavari, Kathmandu (Haruta T)], India [Nilgiri Hill, Gudalur (Hasegawa T); Aritaal, Dalapchand, Sikkim (Haruta T)], the Philippines [Mt. Kitangla, Mindanao; Irawan Palawan], Borneo [Crocker Range, Kota Kinabalu], Indonesia [Sumatra; Mt. Makaweiben, Java; Nr. Tondano, Mt. Makaweiben, Sulawesi], and Australia [4].

### 3.3. Molecular Analyses

#### 3.3.1. Haplotype Diversity

From the 59 *C. pinicolalis* individuals, 12 *COI* haplotypes (BARCPI01–BARCPI12; Table S1), with an average sequence divergence of 0.62% (range, 0.15–1.68%), were obtained (Table 1; Table S4). In the case of *C. punctiferalis*, 15 *COI* haplotypes (BARCPU01–BARCPU15; Table S1), with an average sequence divergence of 0.58% (range, 0.15–1.53%), were obtained from 98 individuals (Table 1; Table S4). When available public data for *C. punctiferalis* were included (18 haplotypes from Korea, China, Pakistan, Australia, Japan, and Thailand), 33 haplotypes showed an average intraspecific divergence of 2.50% (range, 0.15–5.80%), triggering an abrupt increase in the average and range of divergence (Table 1). This result depended on ten haplotypes that originated from Australia; when these ten haplotypes were excluded, the average and range of divergence dropped to levels similar to those obtained from the data of the current study (Table 1). Thus, these haplotypes were excluded from the subsequent divergence comparison between *Conogethes* species, to avoid overestimating species divergence. Excluding the ten haplotypes originating from Australia, the average sequence divergence between *C. pinicolalis* and *C. punctiferalis* was 5.46% (range, 4.89–6.26%), suggesting substantial genetic divergence between *C. pinicolalis* and *C. punctiferalis* (Table 1).

With the inclusion of *COI* sequences of other *Conogethes* species (43 haplotypes for nine species; Table 1), the average interspecific divergence of *C. pinicolalis* from other *Conogethes* species ranged from 5.43 (*C. pandamalis*) to 11.52% (*C. tharsalea*) and that of *C. punctiferalis* ranged from 5.01 (*C. semifascialis*) to 9.60% (*C. haemactalis*), indicating that *C. pinicolalis* and *C. punctiferalis* are equidistant to many other *Conogethes* species pairs (Table 1).

For *EF1α*, 4–13 clones per individual were sequenced for 12 *C. pinicolalis* individuals collected from eight localities (Table S1; Figure 1). As a result, a total of 48 haplotypes, consisting of 42 translated (EF1ACPI01–EF1ACPI42) and six untranslated haplotypes (PI1–PI6), were obtained, corresponding to 2–9 haplotypes per individual (Table S1). The average intraspecific divergence of *C. pinicolalis*, including both translated and untranslated haplotypes, was 0.87% (range, 0.13–5.33%), and excluding untranslated haplotypes, this was 0.86% (range, 0.13–5.33%); finally, that of untranslated haplotypes alone was 0.93% (range, 0.64–1.27%) (Table 2; Table S5). Thus, the divergence of the untranslated haplotypes, which are non-functional gene copies, was only slightly larger than that of the translated haplotypes, but well within the range of divergence found among translated haplotypes. It is noteworthy that the maximum sequence divergence among *EF1α* haplotypes in *C. pinicolalis* is unusually high (5.33%; Table 2). This remarkable divergence was caused by the comparison of the highly divergent EF1ACPI18 haplotype to the others. When EF1ACPI18 was excluded, the intraspecific divergence dropped, ranging from 0.13 to 2.03% (1–16 nucleotide positions). This haplotype was detected with limited frequency and geographic distribution in only one of the five clones of each of the two individuals collected at Mt. Jayangsan (locality 14) and Geoje (locality 15) (Table S1; Figure 1). In the case of *C. punctiferalis*, 5–14 clones collected from 15 individuals across nine localities were sequenced. As a result, a total of 62 haplotypes, consisting of 55 translated (EF1ACPU01–EF1ACPU55) and seven untranslated haplotypes (PU1–PU7), were obtained, corresponding to 2–7 haplotypes per individual (Table S1). The average intraspecific divergence of *C. punctiferalis*, including both translated and untranslated haplotypes, was 0.66% (range, 0.13–1.52%), and excluding untranslated haplotypes, this was 0.63% (range, 0.13–1.52%); finally, that of untranslated haplotypes alone was 0.91% (range, 0.51–1.14%) (Table 2; Table S5). Therefore, the intraspecific divergence of *C. punctiferalis* showed slightly larger variation among untranslated haplotypes but was well within the range of divergence found among translated haplotypes.

The average sequence divergence of *EF1α* haplotypes between *C. punctiferalis* and *C. pinicolalis*, including both translated and untranslated haplotypes, was 2.11% (range, 1.02–5.96%; 8–47 nucleotide positions), and excluding untranslated haplotypes, this was 2.10% (range, 1.02–5.96%; 8–47 nucleotide positions); finally, that of untranslated haplotypes alone was 2.25% (range, 1.90–2.54%; 15–20 nucleotide positions) (Table 2; Table S5). These results also indicate that sequence divergence between the two species increased only slightly when untranslated haplotypes were included. Excluding the highly divergent EF1ACPI18 haplotype of *C. pinicolalis*, the average sequence divergence of translated haplotypes alone between the two species was 2.10% (range, 1.40–2.92%; 11–23 nucleotide positions).

#### 3.3.2. Haplotype Relationships

**Figure 6:**
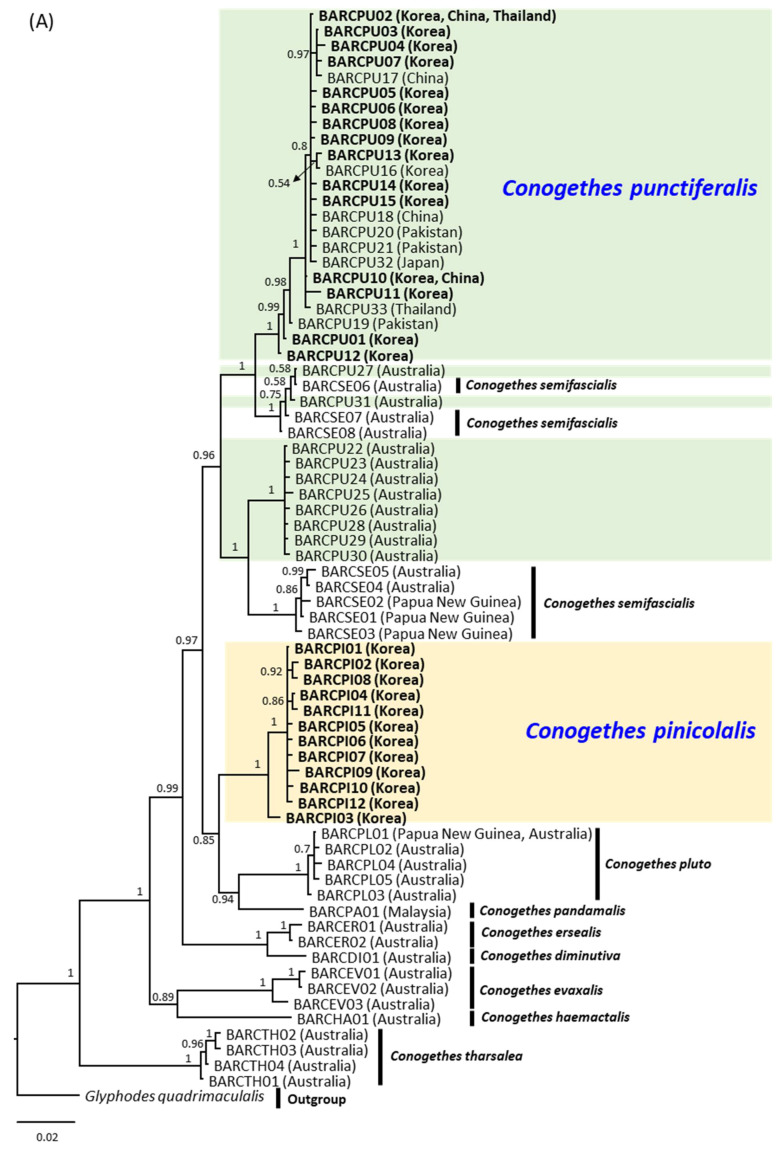

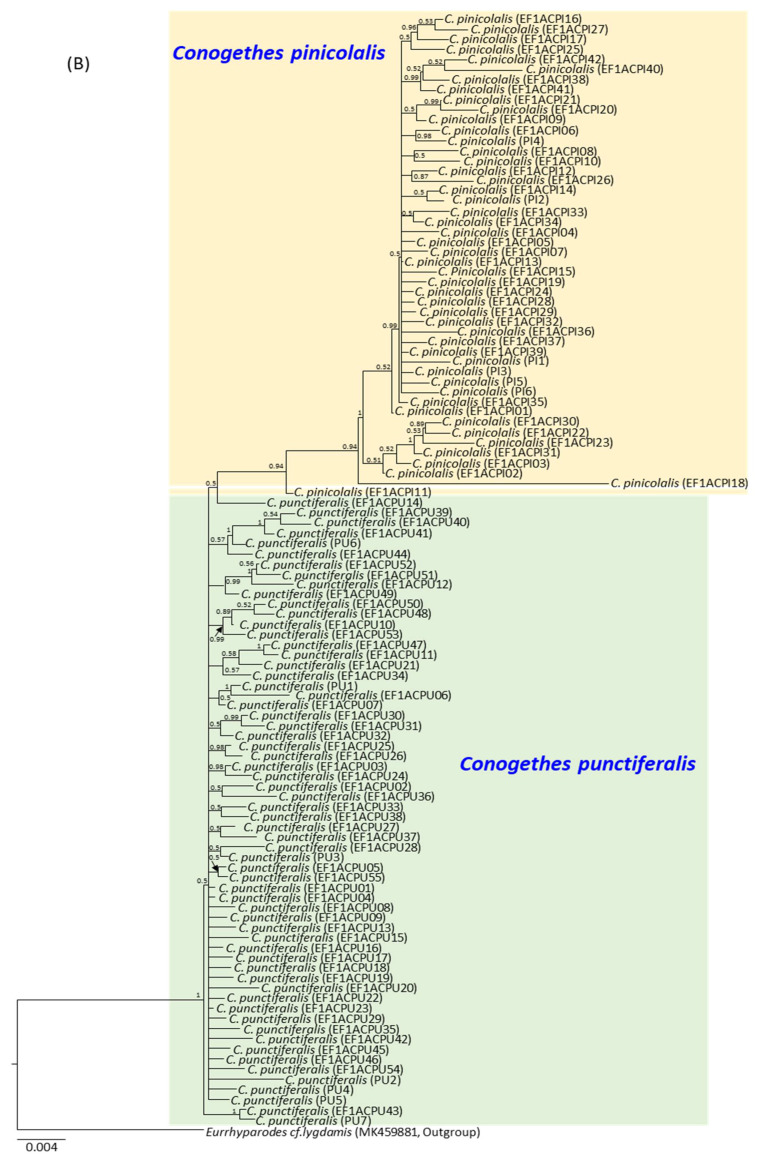
Phylogenetic relationships among *Conogethes pinicolalis* and *C. punctiferalis* haplotypes using Bayesian inference. (**A**) *COI* haplotype-based phylogenetic tree. The analysis was performed with the haplotypes obtained in this study (bold) and those retrieved from GenBank and BOLD Systems, in addition to available sequences from other *Conogethes* species. Text within parentheses indicates the origin of sequences; (**B**) *EF1α* haplotype-based analysis with translated and untranslated haplotypes. Text within parentheses indicates the haplotype names. Values on nodes and pointed by arrows indicate Bayesian posterior probabilities (BPPs, in percentage). The scale bar indicates the number of substitutions per site.

Phylogenetic analysis to illustrate the relationships between *C. pinicolalis* and *C. punctiferalis* was performed using all the 12 and 15 *COI* haplotypes, respectively, which were obtained during this study, along with those of several *Conogethes* species obtained from public data. The 15 haplotypes obtained in this study (BARCPU01–BARCPU15) and 18 *C. punctiferalis* haplotypes retrieved from public data, which originated from Korea, China, Pakistan, Japan, and Thailand, formed a tight monophyletic group with the highest nodal support (BPP = 1.0) (Figure 6A). However, ten *C. punctiferalis* haplotypes originating from Australia were divided into two groups: one group commingled with Australian haplotypes of *C. semifascialis* and another group consisted solely of *C. punctiferalis* haplotypes originating from Australia. The former group was placed as the sister to Australia-excluded monophyletic *C. punctiferalis* group. Indeed, a substantial *COI* sequence divergence of *C. punctiferalis* between Australia and Asia was reported (~6%) [38,39] and also is supported in this study, providing an average divergence of 4.78% (range, 2.29–5.80%). In the case of *C. pinicolalis*, the 12 haplotypes obtained in this study also formed a single group with the highest nodal support (BPP = 1.0) (Figure 6A). This group was placed as the sister to a group consisting of *C. pluto* and *C. pandamalis*.

Phylogenetic analysis using both translated and untranslated *EF1α* haplotypes of *C. pinicolalis* and *C. punctiferalis* showed that *C. pinicolalis* formed a tight monophyletic group with high nodal support (BPP = 0.94) (Figure 6B). EF1ACPI18, the most divergent of all *C. pinicolalis* haplotypes, was placed as the second most basal lineage, with the longest branch length, but was still included in the *C. pinicolalis* group. Furthermore, six untranslated haplotypes of *C. pinicolalis* (Table 2) were included in the *C. pinicolalis* group. On the other hand, the haplotypes of *C. punctiferalis* did not form any strongly inclusive group (BPP = 0.5) and one haplotype, EF1ACPU14, was even placed as the most basal lineage of *C. pinicolalis* haplotypes, although the nodal support was weak (BPP = 0.5; Figure 6B). Nevertheless, the average sequence divergence of EF1ACPU14 from conspecific haplotypes was much smaller (0.53%; range, 0.25–0.89%) than that from *C. pinicolalis* haplotypes (1.85%; range, 1.02–5.33%) (Table S5). When untranslated haplotypes of *EF1α* were removed, the nodal support for the *C. pinicolalis* group dropped to 0.63, whereas that for the *C. punctiferalis* group increased to 0.67, leading to the formation of an additional inclusive group, with respect to the results obtained with the inclusion of untranslated haplotypes (Figure S2). Furthermore, the equivocal EF1ACPU14 haplotype was now clearly included in the *C. punctiferalis* group.

#### 3.3.3. Population Structure

**Figure 7:**
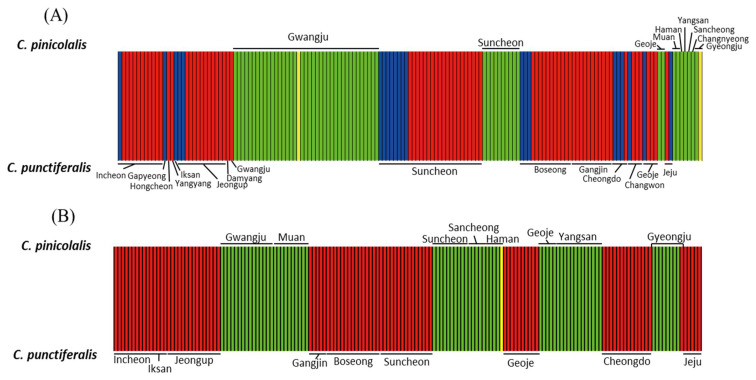
Bayesian clustering analysis. (**A**) *COI* haplotype-based analysis of *Conogethes pinicolalis* and *C. punctiferalis*. The green and yellow haplogroups were detected in *C. pinicolalis,* whereas the blue and red haplogroups were detected in *C. punctiferalis* with an optimum number of clusters (*K*) of 4. (**B**) *EF1α* haplotype-based analysis with translated and untranslated haplotypes of *C. pinicolalis* and *C. punctiferalis. EF1α* haplotype-based analysis included 48 haplotypes, consisting of 42 translated and six untranslated haplotypes, from 12 *C. pinicolalis* individuals, as well as 62 haplotypes, consisting of 55 translated and seven untranslated haplotypes, from 15 *C. punctiferalis* individuals. The green and yellow haplogroups were detected in *C. pinicolalis,* whereas the red haplogroup was detected in *C. punctiferalis,* with a *K* of 3. Note that one of the four clones (EF1ACPI11) of a *C. pinicolalis* individual collected at Gyeongju (locality 20) was assigned to the *C. punctiferalis* haplogroup (red). Each vertical bar represents an individual and its associated probability of belonging to the assigned cluster.

Bayesian analysis of *COI* haplotype clusters to understand the clustering pattern of *C. pinicolalis* and *C. punctiferalis* showed that the optimal *K* value was 4, suggesting that the *Conogethes* (*C. punctiferalis* and *C. pinicolalis*) individuals collected from 21 populations formed four *COI* haplotype clusters, hereafter referred to as haplogroups (Figure 7A). The assignment results for *K* = 4 showed that each species had two haplogroups (green and yellow for *C. pinicolalis*; red and blue for *C. punctiferalis*) independent from each other, regardless of the geographic location of sample collection (Figure 7A). In some localities both species were collected at a single sampling site via pheromone luring and from fallen fruits (e.g., Gwangju, Suncheon, and Geoje), but they were clearly assigned to different *COI* haplogroups, in strong agreement with species identity (Figure 7A).

Bayesian analysis of *EF1α* haplotype clusters was performed using two methods, by either including or excluding untranslated haplotypes, which provided an identical result. The assignment results including both translated and untranslated *EF1α* haplotypes for *K* = 3 showed that two haplogroups (green and yellow) were found only in *C. pinicolalis*, whereas the third haplogroup (red) was detected only in *C. punctiferalis* (Figure 7B; Figure S3). The yellow haplogroup was represented only by a single haplotype (EF1ACPI18) of *C. pinicolalis*, which was highly divergent from other *C. pinicolalis* haplotypes (Table 2; Figure 6B), but was still correctly assigned to *C. pinicolalis*. In Suncheon and Geoje, both species were sampled but they clearly separated into two clusters based on species identity (Figure 6B). On the other hand, EF1ACPI11, which was detected in one of four clones of an individual collected from Gyeongju (locality 20; Table S1; Figure 1) that was recognized as *C. pinicolalis* according to *COI*- and *EF1α*-based analyses including phylogenetic analysis (Figure 6B; Figure S2), was assigned to the red haplogroup, which was attributed exclusively to *C. punctiferalis* (Figure 7B; Figure S2). Indeed, the sequence divergence of EF1ACPI11 from other *C. pinicolalis* haplotypes ranged from 1.14 (9 bp) to 4.85% (39 bp), while ranging from 1.02 (8 bp) to 1.65% (13 bp) when compared to *C. punctiferalis* haplotypes, thereby showing a closer relationship to the latter (Table S5). Except this haplotype, the other haplotypes were clearly assigned to each conspecific haplogroup.

**Figure 8:**
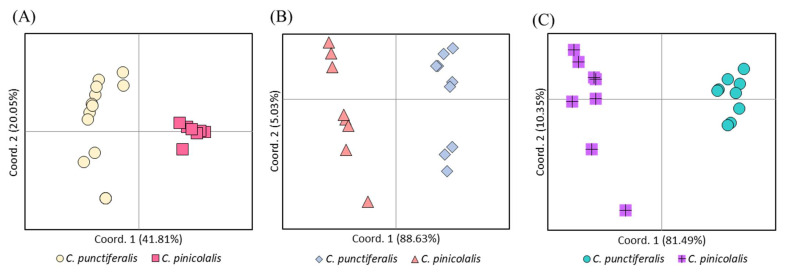
Principal coordinate analysis of individual populations of *Conogethes pinicolalis* and *C. punctiferalis*. (**A**) *COI*-based analysis using 24 populations; (**B**) *EF1α*-based analysis with translated and untranslated haplotypes using 17 populations; (**C**) *EF1α*-based analysis with translated haplotypes only using 17 populations. The variance explained by the first and second components is indicated on the X and Y axes, respectively.

A PCoA [30] was performed using the *F*_ST_ among *C. punctiferalis* and *C. pinicolalis* populations (Figure 8). The first component of the *COI*-based analysis accounted for 41.81% of the variance and clearly explained the divergence of *C. punctiferalis* from *C. pinicolalis*, whereas the second component, which accounted only for 20.05% of the variance, did not correlate to the divergence of the two species (Figure 8A). *EF1α*-based analyses, both including and excluding untranslated haplotypes, showed clear divergence of the two species along the first component (explaining 88.15 and 81.49% of the variance, respectively), clearly indicating genetic divergence between *C. punctiferalis* and *C. pinicolalis* populations (Figure 8B,C).

## 4. Discussion

The recognition that *C. punctiferalis* and *C. pinicolalis* are distinct species provides the basis for further fundamental investigations of their biology and for establishing effective methods for pest management. *C. pinicolalis* was originally considered one of the ecotypes (PFT) of *C. punctiferalis*, but it was subsequently redescribed as a new species [4]. From a morphological perspective, *C. pinicolalis* is distinct from *C. punctiferalis* in several features, such as the second segment of the labial palpus (Figure 4E), the presence of a large fuscous tuft on the male hind tibia (Figure 4G), and a long aedeagus in the male genitalia (Figure 5A) [4]. Thus, *C. pinicolalis* is clearly distinct from *C. punctiferalis*, morphologically. Consequently, we newly assigned the Korean name So-na-mu-deul-myeong-na-bang to *C. pinicolalis*.

From an ecological perspective, the observation and collection of larvae and adults on pine trees from April to July (Figure 2C), and the success of indoor culture of larvae at different stages until adult emergence and egg deposition by providing pine needles (Figure 3) confirmed that the host of *C. pinicolalis* is the pine tree, at least during the first generation. On the other hand, all the larvae collected inside peaches and apricots during the same period belonged to *C. punctiferalis*, indicating an obvious ecological isolation between these two species, at least for the first generation. However, it was obvious that *C. punctiferalis* develops in chestnuts during the second generation, since adults were collected between September and October 2018 and overwintering larvae were observed inside fallen chestnuts, between single chestnuts, and inside chestnut clusters in October 2018 (Table S1). Furthermore, other studies also detected *C. punctiferalis* larvae infecting chestnuts at the second generation [37,40]. However, we did not investigate the host of the second generation of *C. pinicolalis*; therefore, additional studies on this aspect are required.

From a molecular perspective, *COI* sequences showed a substantial average divergence between *C. pinicolalis* and *C. punctiferalis* (Table 2), leading to their separation into independent species in phylogenetic analysis (Figure 6A), independent haplogroups in BAPS (Figure 7A), and separated clusters in PCoA (Figure 8A). The average *COI* sequence divergence of 5.46% between *C. pinicolalis* and *C. punctiferalis* (range, 4.89–6.26%) may not be negligible. Previously, the two species occurring in China also were investigated for their divergence using mitochondrial genes and found 5.75% of minimum sequence divergence in 731-bp portion of *COI* [41]. In fact, Kim et al. [42] compared the sequence divergence of each genetic region of all mitochondrial genes in a lepidopteran superfamily, Bombycoidea, to understand the within-genus sequence divergence. These authors showed that the *COI* sequence divergence between species of the same genus ranged approximately from 4 to 7.5%, suggesting this gene to be a good indicator for species delimitation, although this divergence was the fifth lowest among the 15 mitochondrial genes analyzed.

The divergence of *EF1α* between *C. pinicolalis* and *C. punctiferalis* was lower than that of *COI*, presenting an average of 2.11 and 2.10% when including and excluding untranslated haplotypes, respectively (Table 2). Considering that, due to biparental inheritance, the coalescence time of nuclear DNA is approximately four times longer than that of mitochondrial DNA, which is transmitted maternally, substantially lower divergence in *EF1α* could be inevitable, although a precise explanation of this phenomenon may require further rigorous study [43]. Indeed, Kim et al. [44] compared the sequence divergence of *COI* and *EF1α* in *Stathmopoda* species (Lepidoptera: Stathmopodidae) and found that the average interspecific divergence of *EF1α* was 7.5% (range, 5.2–10.9%), whereas that of *COI* was 11.8% (range, 8.2–16.1%) [44], indicating obviously higher variation in the mitochondrial gene *COI* than in the nuclear gene *EF1α*.

In this study, most of the *EF1α* haplotypes were well within the conspecific divergence range and were placed properly in conspecific groups during phylogenetic analysis, supporting the separation of each species as independent; however, exceptions were also found. In particular, EF1ACPI11, which originated from *C. pinicolalis*, showed a rather close genetic relationship to the haplotypes of *C. punctiferalis* (Table 2), although it was positioned as the most basal haplotype within the *C. pinicolalis* group in the *EF1α*-based phylogenetic tree including both untranslated and translated haplotypes (Figure 6B); moreover, this haplotype was assigned to a *C. punctiferalis* haplogroup in BAPS analysis (Figure 7B; Figure S3). Furthermore, EF1ACPU14 from *C. punctiferalis* was placed as the most basal lineage of *C. pinicolalis* haplotypes in the *EF1α*-based phylogenetic tree including both untranslated and translated haplotypes (Figure 6B), although the sequence divergence of EF1ACPU14 to conspecific haplotypes was much smaller than that to *C. pinicolalis* haplotypes (Table 2). Finally, EF1ACPI18 from *C. pinicolalis* was exceptionally divergent from other conspecific haplotypes (5.8%) and was placed as the second most basal haplotype within the *C. pinicolalis* clade, with the longest branch length (Table 2; Figure 6B; Figure S2). Probably, these equivocal haplotypes indicate that not enough time has elapsed for complete lineage sorting of the two species in terms of *EF1α* divergence, which requires a coalescence time four times longer than that of mitochondrial DNA [43]. Paradoxically, the short divergence time between these two species may be reflected in the fact that *EF1α* untranslated haplotypes were only slightly more divergent than those of translated sequences (Table 2). Overall, *EF1α*-based analyses showed that *C. pinicolalis* has substantially diverged from *C. punctiferalis,* but were not as complete as *COI* analysis. Consistent with *EF1α*-based analyses, a mating experiment showed that several eggs were produced only when crossing FFT females and PFT males, with an unusually higher male:female ratio in the F_1_ [15]. Similarly, the female pheromone of *C. punctiferalis* was effective to attract males of both species [13]; this was also observed during the current study.

Similar to *C. pinicolalis* and *C. punctiferalis*, examples of lepidopteran species showing incomplete speciation have often been reported. For example, species of *Helicoverpa* were fundamentally distinguished based on the morphology of male and female genitalia [45]. Among them, *H. zea* and *H. armigera* can be distinguished by observing the male abdomen and genitalia, and in particular, the difference in the shape of the eighth sternite margin: in fact, *H. zea* has a V-shaped sternite margin, whereas *H. armigera* has a U-shaped sternite margin [46]. In addition, the mean valve length of the male genitalia is 4.98 mm for *H. zea* and 4.48 mm for *H. armigera* [46]. Furthermore, *COI* and triosephosphate isomerase also indicated clear divergence between these two species in network analysis [47]. Nevertheless, hybrid offspring between *H. zea* and *H. armigera* was also detected through a microsatellite DNA-based analysis [48]. Structure analysis to explain the genetic identity of each species showed that individuals of *H. zea* and *H. armigera* are mostly identifiable; however, one *H. zea* and nine *H. armigera* individuals showed a genetic similarity of more than 50%, indicating that they arose from hybridization between these two species [48].

Considering that *C. pinicolalis* and *C. punctiferalis* clearly differ in their host plants at least for the first generation the control strategy should be established independently for each species. In particular, a new control strategy for *C. pinicolalis* on pines is required in that current control methods in Korea have been focused only on *C. punctiferalis* infesting non-pine trees [8,9,10,37].

## 5. Conclusions

The current study indicates that *C. pinicolalis* clearly has a different host than that of *C. punctiferalis*, at least for the first generation. In the field, larvae and adults of *C. pinicolalis* were detected on pine trees, whereas *C. punctiferalis* individuals were found inside peaches and apricots in the first generation and inside chestnuts in the second generation. In addition, the growth of *C. pinicolalis* larvae on pine tree leaves until egg deposition further confirmed that pine trees are the hosts of *C. pinicolalis*, at least for the first generation. Morphological differences also indicated the divergence of *C. pinicolalis* from *C. punctiferalis*, consistent with previous reports [4,41]. Further, *COI*-based analyses clearly evidenced substantial divergence between these two species. Thus, morphological and molecular detection may be effective methods to distinguish these two pests. On the other hand, although *EF1α*-based analyses showed a distinct separation between these two species, indications of incomplete speciation were also observed. Collectively, our results confirmed that the PFT of *C. punctiferalis* occurring in Korea is *C. pinicolalis*; however, additional studies are needed to identify the host plant for the second generation and the overwintering niche of *C. pinicolalis*.

## Figures and Tables

**Table 1:** Summary estimates of *COI* sequence divergence (%) within and between *Conogethes* species.

Taxon	No. Haplotype	Min.	Max.	Aver.
Intraspecific divergence in each *Conogethes* species				
*Conogethes punctiferalis* (current study only)	15	0.15	1.53	0.58
*Conogethes punctiferalis* (current study and public data)	33	0.15	5.80	2.50
*Conogethes punctiferalis* (current study and public data, excluding Australian haplotypes)	23	0.15	1.53	0.56
*Conogethes pinicolalis*	12	0.15	1.68	0.62
*Conogethes pluto*	5	0.15	0.46	0.31
*Conogethes semifascialis*	8	0.15	5.19	2.78
*Conogethes tharsalea*	4	0.15	0.61	0.43
*Conogethes ersealis*	2	-	-	0.46
*Conogethes evaxalis*	3	0.15	1.83	1.22
Interspecific divergence between *Conogethes* species				
*C. punctiferalis* and *C. pinicolalis* (current study only)		4.89	6.26	5.44
*C. punctiferalis* and *C. pinicolalis* (current study and public data)		4.89	7.02	5.76
*C. punctiferalis* and *C. pinicolalis* (current study and public data, excluding Australian haplotypes)		4.89	6.26	5.46
*C. punctiferalis* and *C. pluto*		5.34	6.72	6.15
*C. punctiferalis* and *C. semifascialis*		0.15	5.80	4.25
*C. punctiferalis* and *C. tharsalea*		9.16	10.69	10.06
*C. punctiferalis* and *C. ersealis*		7.18	8.55	7.72
*C. punctiferalis* and *C. evaxalis*		7.63	10.23	8.83
*C. punctiferalis* and *C. diminutiva*		7.79	8.86	8.36
*C. punctiferalis* and *C. haemactalis*		9.31	10.08	9.63
*C. punctiferalis* and *C. pandamalis*		5.65	7.18	6.20
*C. punctiferalis* and *C. pluto* (excluding Australian haplotypes ^1^)		5.34	6.72	6.13
*C. punctiferalis* and *C. semifascialis* (excluding Australian haplotypes ^1^)		1.99	5.80	4.38
*C. punctiferalis* and *C. tharsalea* (excluding Australian haplotypes ^1^)		9.16	10.53	9.93
*C. punctiferalis* and *C. ersealis* (excluding Australian haplotypes ^1^)		7.33	8.55	7.94
*C. punctiferalis* and *C. evaxalis* (excluding Australian haplotypes ^1^)		7.79	9.47	8.61
*C. punctiferalis* and *C. diminutive* (excluding Australian haplotypes ^1^)		7.79	8.86	8.32
*C. punctiferalis* and *C. haemactalis* (excluding Australian haplotypes ^1^)		9.31	10.08	9.63
*C. punctiferalis* and *C. pandamalis* (excluding Australian haplotypes ^1^)		5.65	6.41	5.95
*C. punctiferalis* and *C. pluto* (*C. punctiferalis* from current study only)		5.34	6.72	6.12
*C. punctiferalis* and *C. semifascialis* (*C. punctiferalis* from current study only)		1.99	5.80	4.36
*C. punctiferalis* and *C. tharsalea* (*C. punctiferalis* from current study only)		9.16	10.53	9.92
*C. punctiferalis* and *C. ersealis* (*C. punctiferalis* from current study only)		7.33	8.55	7.96
*C. punctiferalis* and *C. evaxalis* (*C. punctiferalis* from current study only)		7.79	9.47	8.60
*C. punctiferalis* and *C. diminutive* (*C. punctiferalis* from current study only)		7.79	8.86	8.29
*C. punctiferalis* and *C. haemactalis* (*C. punctiferalis* from current study only)		9.31	10.08	9.65
*C. punctiferalis* and *C. pandamalis* (*C. punctiferalis* from current study only)		5.65	6.41	5.91
*C. pinicolalis* and *C. pluto*		5.95	6.72	6.51
*C. pinicolalis* and *C. semifascialis*		5.04	6.57	5.89
*C. pinicolalis* and *C. tharsalea*		10.99	12.06	11.52
*C. pinicolalis* and *C. ersealis*		6.87	7.94	7.29
*C. pinicolalis* and *C. evaxalis*		7.94	9.47	8.85
*C. pinicolalis* and *C. diminutive*		7.48	8.24	7.77
*C. pinicolalis* and *C. haemactalis*		9.01	9.77	9.37
*C. pinicolalis* and *C. pandamalis*		4.89	5.80	5.43
*C. pluto* and *C. semifascialis*		6.11	6.87	6.44
*C. pluto* and *C. tharsalea*		10.69	11.45	11.04
*C. pluto* and *C. ersealis*		6.87	7.63	7.27
*C. pluto* and *C. evaxalis*		9.16	9.92	9.56
*C. pluto* and *C. diminutiva*		8.24	8.70	8.43
*C. pluto* and *C. haemactalis*		9.77	10.23	10.02
*C. pluto* and *C. pandamalis*		5.80	6.11	5.98
*C. semifascialis* and *C. tharsalea*		9.93	11.45	10.67
*C. semifascialis* and *C. ersealis*		6.72	8.86	8.00
*C. semifascialis* and *C. evaxalis*		7.48	9.92	9.10
*C. semifascialis* and *C. diminutiva*		7.63	9.31	8.66
*C. semifascialis* and *C. haemactalis*		9.01	9.47	9.18
*C. semifascialis* and *C. pandamalis*		5.50	7.02	6.47
*C. tharsalea* and *C. ersealis*		11.15	11.60	11.38
*C. tharsalea* and *C. evaxalis*		11.60	12.06	11.82
*C. tharsalea* and *C. diminutiva*		11.76	12.06	11.95
*C. tharsalea* and *C. haemactalis*		10.99	11.60	11.34
*C. tharsalea* and *C. pandamalis*		11.60	12.06	11.83
*C. ersealis* and *C. evaxalis*		9.16	9.47	9.29
*C. ersealis* and *C. diminutiva*		2.60	3.05	2.83
*C. ersealis* and *C. haemactalis*		8.70	8.86	8.78
*C. ersealis* and *C. pandamalis*		7.94	8.24	8.09
*C. evaxalis* and *C. diminutiva*		10.23	10.69	10.53
*C. evaxalis* and *C. haemactalis*		9.31	9.62	9.47
*C. evaxalis* and *C. pandamalis*		9.47	9.62	9.52
*C. diminutiva* and *C. haemactalis*		-	-	9.62
*C. diminutiva* and *C. pandamalis*		-	-	8.40
*C. haemactalis* and *C. pandamalis*		-	-	10.23
Within *Conogethes* divergence		0.15	12.06	5.77

-, not available. ^1^ excluding ten haplotypes that originated from Australia, which showed unusually higher sequence divergence.

**Table 2:** Summary estimates of *EF1α* sequence divergence (%) within and between *Conogethes* species.

Taxon	No. Haplotype	Min.	Max.	Aver.
Intraspecific divergence of *C. pinicolalis* (inicluding PI ^1^)	48	0.13	5.33	0.87
Intraspecific divergence of *C. pinicolalis* (excluding PI ^1^)	42	0.13	5.33	0.86
Intraspecific divergence of *C. pinicolalis* (only PI ^1^)	6	0.64	1.27	0.93
Intraspecific divergence of *C. punctiferalis* (including PU ^2^)	62	0.13	1.52	0.66
Intraspecific divergence of *C. punctiferalis* (excluding PU ^2^)	55	0.13	1.52	0.63
Intraspecific divergence of *C. punctiferalis* (only PU ^2^)	7	0.51	1.14	0.91
*C. punctiferalis* and *C. pinicolalis* (including untranslated haplotypes)		1.02	5.96	2.11
*C. punctiferalis* and *C. pinicolalis* (excluding untranslated haplotypes)		1.02	5.96	2.10
*C. punctiferalis* and *C. pinicolalis* (only untranslated haplotypes)		1.90	2.54	2.25

^1^ PI, untranslated *EF1α* haplotypes of *C. pinicolalis*; ^2^ PU, untranslated *EF1α* haplotypes of *C. punctiferalis*.

## Data Availability

The data presented in this study are available in the text and Supplementary Material here.

## Supplementary Materials

The following are available online at https://www.mdpi.com/article/10.3390/insects12050455/s1, Figure S1: Materials used for the investigation of the life cycle of *Conogethes pinicolalis*. (A) Insect breeding dish used for indoor culture of larvae (Ø, 10 cm; height, 4 cm), in addition to pine twigs. (B) Triangular pyramid cage, made of butter paper, for mating and oviposition. (C) Insect breeding cage (20 cm × 20 cm × 15 cm). (D) Insect breeding cage (24.5 cm × 24.5 cm × 63 cm). (E) Gauze-covered pack to induce egg deposition, Figure S2: *EF1α* haplotype-based phylogenetic analysis using solely translated haplotypes and Bayesian inference. Text within parentheses indicates the haplotype names. Values on nodes indicate Bayesian posterior probabilities (in percentage). The scale bar indicates the number of substitutions per site. *Glyphodes quadrimaculalis* (Crambidae) was used as an outgroup, Figure S3: *EF1α* haplotype-based Bayesian clustering analysis of *C. pinicolalis* and *C. punctiferalis.* The analysis was carried out with translated haplotypes only. The green and yellow haplogroups were detected in *C. pinicolalis,* whereas the red haplogroup was detected in *C. punctiferalis*, with an optimum number of clusters (*K*) of 3. Note that one of four clones (EF1ACPI11) of a *C. pinicolalis* individual collected at Gyeongju (locality 20) was assigned to the *C. punctiferalis* haplogroup (red). Each vertical bar represents an individual and its associated probability of belonging to the assigned cluster, Table S1: List of *Conogethes pinicolalis* and *C. punctiferalis* samples sequenced for *COI* and *EF1α*, Table S2: List of *COI* sequences of *Conogethes* species retrieved from GenBank and BOLD Systems, Table S3: Pupal period and lifespan of *Conogethes pinicolalis* (days), Table S4: Pairwise comparisons of *COI* haplotypes of *Conogethes* species, Table S5: Pairwise comparisons of *EF1α* haplotypes of *Conogethes pinicolalis* and *C. punctiferalis*
