# Supplementary material for: Morphological, Ecological, and Molecular Divergence of Conogethes pinicolalis from C. punctiferalis (Lepidoptera: Crambidae)
Source: Insects. 2021 May 15;12(5):455. doi: 10.3390/insects12050455 (PMC8156953; doi:10.3390/insects12050455)
Supplement: Supplementary file 1 [file insects-12-00455-s001.zip › All Tables S1-S5.pdf]

Table S1. List of *Conogethes pinicolalis* and *C. punctiferalis* samples sequenced for *COI* and *EF1α*

Locality (no. of individuals)	Collection date	Sample animal number	*COI* haplotype (658 bp)	GenBank no.	*EF1α* haplotype (787 bp)	GenBank no.
1. Mt. Gyeyangsan, Incheon Metropolitan City (11)	2016. 09. 27	CNU7272	BARCPU01	MW315217	EF1ACPU01	MW348773
	2016. 09. 27	-	-	-	EF1ACPU01	MW348773
	2016. 09. 27	-	-	-	EF1ACPU02	MW348774
	2016. 09. 27	-	-	-	EF1ACPU03	MW348775
	2016. 09 .27	-	-	-	EF1ACPU03	MW348775
	2018. 10. 12	CNU8214	BARCPU13	MW315218	EF1ACPU01	MW348776
	2018. 10. 12	-	-	-	EF1ACPU01	MW348776
	2018. 10. 12	-	-	-	EF1ACPU37	MW348777
	2018. 10. 12	-	-	-	EF1ACPU38	MW348778
	2018. 10. 12	-	-	-	PU5†	MW348779
	2018. 10. 12	CNU8208	BARCPU02	MW315219	-	-
	2018. 10. 12	CNU8209	BARCPU02	MW315220	-	-
	2018. 10. 12	CNU8210	BARCPU02	MW315221	-	-
	2018.10. 12	CNU8211	BARCPU02	MW315222	-	-
	2018.10. 12	CNU8212	BARCPU02	MW315223	-	-
	2018.10. 12	CNU8213	BARCPU02	MW315224	-	-
	2018.10. 12	CNU8215	BARCPU02	MW315225	-	-
	2018.10. 12	CNU8216	BARCPU13	MW315226	-	-
	2018.10. 12	CNU8217	BARCPU02	MW315227	-	-
2. Gapyeong, GG (1)	2018.10. 02	CNU8219	BARCPU02	MW315228	-	-
3. Hongcheon, GW (1)	2018. 07. 31	CNU8203	BARCPU01	MW315229	-	-
4. Yangyang, GW (1)	2018. 09. 24	CNU8218	BARCPU02	MW315230	-	-
5. Iksan, JB (1)	2018. 08. 08	CNU8204	BARCPU11	MW315231	EF1ACPU32	MW348780
	2018. 08. 08	-	-	-	EF1ACPU32	MW348780
	2018. 08. 08	-	-	-	EF1ACPU32	MW348780
	2018. 08. 08	-	-	-	EF1ACPU32	MW348780
	2018. 08. 08	-	-	-	EF1ACPU33	MW348781
6. Jeongeup, JB (14)	2018. 07. 18	CNU8185	BARCPU09	MW315232	EF1ACPU24	MW348782
	2018. 07. 18	-	-	-	EF1ACPU25	MW348783
	2018. 07. 18	-	-	-	EF1ACPU26	MW348784
	2018. 07. 18	-	-	-	EF1ACPU27	MW348785
	2018. 07. 18	-	-	-	EF1ACPU28	MW348786
	2018. 07. 18	CNU8189	BARCPU03	MW315233	EF1ACPU06	MW348787
	2018. 07. 18	-	-	-	EF1ACPU07	MW348788
	2018. 07. 18	-	-	-	EF1ACPU08	MW348789
	2018. 07. 18	-	-	-	EF1ACPU09	MW348790
	2018. 07. 18	-	-	-	EF1ACPU09	MW348790
	2018. 07. 18	-	-	-	EF1ACPU11	MW348791
	2018. 07. 18	-	-	-	EF1ACPU21	MW348792
	2018. 07. 18	-	-	-	EF1ACPU21	MW348792
	2018. 07. 18	-	-	-	EF1ACPU47	MW348793
	2018. 07. 18	-	-	-	PU1†	MW348794
	2018. 07. 18	CNU8179	BARCPU01	MW315234	-	-
	2018. 07. 18	CNU8180	BARCPU02	MW315235	-	-
	2018. 07. 18	CNU8181	BARCPU03	MW315236	-	-
	2018. 07. 18	CNU8182	BARCPU03	MW315237	-	-
	2018. 07. 18	CNU8183	BARCPU02	MW315238	-	-
	2018. 07. 18	CNU8184	BARCPU01	MW315239	-	-
	2018. 07. 18	CNU8186	BARCPU01	MW315240	-	-
	2018. 07. 18	CNU8187	BARCPU09	MW315241	-	-
	2018. 07. 18	CNU8188	BARCPU09	MW315242	-	-
	2018. 07. 18	CNU8191	BARCPU02	MW315243	-	-
	2018. 07. 18	CNU8192	BARCPU02	MW315244	-	-
	2018. 07. 18	CNU8194	BARCPU02	MW315245	-	-
7. Damyang, JN (1)	2018. 10. 15	CNU8238	BARCPU02	MW315246	-	-
8. Gwangju Metropolitan City (40)	2018. 05. 22	CNU8202	BARCPU02	MW315247	-	-
	2019. 04. 20	CNU10685	BARCPI11	MW315248	EF1ACPI30	MW348795
	2019. 04. 20	-	-	-	EF1ACPI30	MW348795
	2019. 04. 20	-	-	-	EF1ACPI30	MW348795
	2019. 04. 20	-	-	-	EF1ACPI31	MW348796
	2019. 04. 20	-	-	-	EF1ACPI31	MW348796
	2019. 04. 20	CNU10689	BARCPI01	MW315249	EF1ACPI01	MW348797
	2019. 04. 20	-	-	-	EF1ACPI02	MW348798

	2019. 04. 20	-	-	-	EF1ACPI03	MW348799
	2019. 04. 20	-	-	-	EF1ACPI04	MW348800
	2019. 04. 20	-	-	-	EF1ACPI04	MW348800
	2019. 04. 20	CNU10690	BARCPI10	MW315250	EF1ACPI25	MW348801
	2019. 04. 20	-	-	-	EF1ACPI26	MW348802
	2019. 04. 20	-	-	-	EF1ACPI27	MW348803
	2019. 04. 20	-	-	-	EF1ACPI28	MW348804
	2019. 04. 20	-	-	-	EF1ACPI29	MW348805
	2019. 04. 20	CNU10684	BARCPI04	MW315251	-	-
	2019. 04. 20	CNU10686	BARCPI12	MW315252	-	-
	2019. 04. 20	CNU10687	BARCPI04	MW315253	-	-
	2019. 04. 20	CNU10688	BARCPI01	MW315254	-	-
	2019. 04. 20	CNU10691	BARCPI12	MW315255	-	-
	2019. 04. 20	CNU10692	BARCPI01	MW315256	-	-
	2019. 04. 20	CNU10693	BARCPI04	MW315257	-	-
	2019. 04. 20	CNU10694	BARCPI04	MW315258	-	-
	2019. 04. 20	CNU10695	BARCPI01	MW315259	-	-
	2019. 04. 20	CNU10696	BARCPI09	MW315260	-	-
	2019. 04. 20	CNU10697	BARCPI01	MW315261	-	-
	2019. 04. 20	CNU10698	BARCPI01	MW315262	-	-
	2019. 04. 20	CNU10699	BARCPI01	MW315263	-	-
	2019. 04. 20	CNU10700	BARCPI09	MW315264	-	-
	2019. 04. 20	CNU10701	BARCPI01	MW315265	-	-
	2019. 05. 01	CNU10702	BARCPI01	MW315266	-	-
	2019. 05. 01	CNU11057	BARCPI01	MW315267	-	-
	2019. 05. 01	CNU11058	BARCPI01	MW315268	-	-
	2019. 05. 01	CNU11059	BARCPI04	MW315269	-	-
	2019. 05. 01	CNU11060	BARCPI01	MW315270	-	-
	2019. 04. 20	CNU11061	BARCPI01	MW315271	-	-
	2019. 04. 20	CNU11062	BARCPI09	MW315272	-	-
	2019. 04. 20	CNU11063	BARCPI04	MW315273	-	-
	2019. 04. 20	CNU11064	BARCPI01	MW315274	-	-
	2019. 04. 20	CNU11065	BARCPI01	MW315275	-	-
	2019. 04. 20	CNU11066	BARCPI01	MW315276	-	-
	2019. 04. 20	CNU11067	BARCPI03	MW315277	-	-
	2019. 04. 20	CNU11068	BARCPI04	MW315278	-	-
	2019. 04. 20	CNU11069	BARCPI04	MW315279	-	-
	2019. 04. 20	CNU11070	BARCPI04	MW315280	-	-
	2019. 04. 20	CNU11071	BARCPI04	MW315281	-	-
	2019. 04. 20	CNU11072	BARCPI05	MW315282	-	-
	2019. 04. 20	CNU11073	BARCPI01	MW315283	-	-
	2019. 04. 20	CNU11074	BARCPI04	MW315284	-	-
	2019. 04. 20	CNU11075	BARCPI12	MW315285	-	-
	2019. 04. 20	CNU11076	BARCPI09	MW315286	-	-
9. Mt. Seungdalsan, Muan; JN (2)	2018. 08. 19	CNU8482	BARCPI04	MW315287	EF1ACPI10	MW348806
	2018. 08. 19	-	-	-	EF1ACPI10	MW348806
	2018. 08. 19	-	-	-	EF1ACPI12	MW348807
	2018. 08. 19	-	-	-	EF1ACPI12	MW348807
	2018. 08. 19	-	-	-	EF1ACPI12	MW348807
	2018. 08. 19	-	-	-	EF1ACPI12	MW348807
	2018. 08. 19	-	-	-	EF1ACPI13	MW348808
	2018. 08. 19	-	-	-	EF1ACPI35	MW348809
	2018. 08. 19	-	-	-	EF1ACPI36	MW348810
	2018. 08. 19	-	-	-	EF1ACPI37	MW348811
	2018. 08. 19	CNU8483	BARCPI05	MW315288	-	-
10. Gangjin, JN (11)	2018. 10. 18	CNU8240	BARCPU02	MW315289	EF1ACPU01	MW348812
	2018. 10. 18	-	-	-	EF1ACPU01	MW348812
	2018. 10. 18	-	-	-	EF1ACPU01	MW348812
	2018. 10. 18	-	-	-	EF1ACPU04	MW348813
	2018. 10. 18	-	-	-	EF1ACPU05	MW348814
	2018. 10. 18	CNU8239	BARCPU13	MW315290	-	-
	2018. 10. 18	CNU8241	BARCPU13	MW315291	-	-
	2018. 10. 18	CNU8242	BARCPU02	MW315292	-	-
	2018. 10. 18	CNU8243	BARCPU02	MW315293	-	-
	2018. 10. 18	CNU8244	BARCPU13	MW315294	-	-
	2018. 10. 18	CNU8245	BARCPU02	MW315295	-	-
	2018. 10. 18	CNU8246	BARCPU02	MW315296	-	-

	2018. 10. 18	CNU8247	BARCPU02	MW315297	-	-
	2018. 10. 18	CNU8248	BARCPU02	MW315298	-	-
	2018. 10. 18	CNU8249	BARCPU02	MW315299	-	-
11. Boseong, JN (14)	2018. 06. 28	CNU8160	BARCPU01	MW315300	-	-
	2018. 06. 28	CNU8161	BARCPU04	MW315301	EF1ACPU10	MW348815
	2018. 06. 28	-	-	-	EF1ACPU10	MW348815
	2018. 06. 28	-	-	-	EF1ACPU10	MW348815
	2018. 06. 28	-	-	-	EF1ACPU12	MW348816
	2018. 06. 28	-	-	-	EF1ACPU48	MW348817
	2018. 06. 28	-	-	-	EF1ACPU49	MW348818
	2018. 06. 28	-	-	-	EF1ACPU50	MW348819
	2018. 06. 28	-	-	-	EF1ACPU51	MW348820
	2018. 06. 28	-	-	-	EF1ACPU52	MW348821
	2018. 06. 28	-	-	-	EF1ACPU53	MW348822
	2018. 06. 28	CNU8254	BARCPU05	MW315302	EF1ACPU01	MW348823
	2018. 06. 28	-	-	-	EF1ACPU13	MW348824
	2018. 06. 28	-	-	-	EF1ACPU14	MW348825
	2018. 06. 28	-	-	-	EF1ACPU15	MW348826
	2018. 06. 28	-	-	-	PU2†	MW348827
	2018. 06. 28	CNU8162	BARCPU02	MW315303	-	-
	2018. 06. 28	CNU8163	BARCPU03	MW315304	-	-
	2018. 06. 28	CNU8166	BARCPU02	MW315305	-	-
	2018. 06. 28	CNU8167	BARCPU02	MW315306	-	-
	2018. 06. 28	CNU8168	BARCPU02	MW315307	-	-
	2018. 06. 28	CNU8169	BARCPU01	MW315308	-	-
	2018. 06. 28	CNU8170	BARCPU05	MW315309	-	-
	2018. 06. 28	CNU8250	BARCPU02	MW315310	-	-
	2018. 06. 28	CNU8251	BARCPU02	MW315311	-	-
	2018. 06. 28	CNU8252	BARCPU02	MW315312	-	-
	2018. 06. 28	CNU8253	BARCPU01	MW315313	-	-
12. Suncheon, JN (38)	2018. 06. 08	CNU8195	BARCPU10	MW315314	EF1ACPU29	MW348828
	2018. 06. 08	-	-	-	EF1ACPU30	MW348829
	2018. 06. 08	-	-	-	EF1ACPU30	MW348829
	2018. 06. 08	-	-	-	EF1ACPU31	MW348830
	2018. 06. 08	-	-	-	EF1ACPU31	MW348830
	2018. 10. 03	CNU8229	BARCPU14	MW315315	EF1ACPU39	MW348831
	2018. 10. 03	-	-	-	EF1ACPU40	MW348832
	2018. 10. 03	-	-	-	EF1ACPU41	MW348833
	2018. 10. 03	-	-	-	EF1ACPU42	MW348834
	2018. 10. 03	-	-	-	PU6†	MW348835
	2018. 10. 03	CNU8230	BARCPU15	MW315316	EF1ACPU43	MW348836
	2018. 10. 03	-	-	-	EF1ACPU44	MW348837
	2018. 10. 03	-	-	-	EF1ACPU45	MW348838
	2018. 10. 03	-	-	-	EF1ACPU46	MW348839
	2018. 10. 03	-	-	-	PU7†	MW348840
	2012. 06. 20	CP4545	BARCPU01	MW315317	-	-
	2012. 06. 20	CP4546	BARCPU01	MW315318	-	-
	2012. 06. 20	CP4547	BARCPU01	MW315319	-	-
	2012. 06. 20	CP4548	BARCPU01	MW315320	-	-
	2012. 06. 20	CP4549	BARCPU02	MW315321	-	-
	2012. 06. 20	CP4550	BARCPU01	MW315322	-	-
	2012. 06. 20	CP4556	BARCPU02	MW315323	-	-
	2012. 06. 20	CP4557	BARCPU01	MW315324	-	-
	2012. 06. 20	CP4558	BARCPU01	MW315325	-	-
	2012. 06. 20	CP4559	BARCPU03	MW315326	-	-
	2012. 06. 20	CP4560	BARCPU02	MW315327	-	-
	2012. 06. 20	CP4561	BARCPU02	MW315328	-	-
	2018. 06. 08	CNU8200	BARCPU01	MW315329	-	-
	2018. 06. 13	CNU8201	BARCPU02	MW315330	-	-
	2018. 10. 03	CNU8225	BARCPU02	MW315331	-	-
	2018. 10. 03	CNU8226	BARCPU02	MW315332	-	-
	2018. 10. 03	CNU8227	BARCPU02	MW315333	-	-
	2018. 10. 03	CNU8228	BARCPU02	MW315334	-	-
	2018. 10. 03	CNU8231	BARCPU02	MW315335	-	-
	2018. 10. 03	CNU8232	BARCPU02	MW315336	-	-
	2018. 10. 03	CNU8233	BARCPU02	MW315337	-	-

	2018. 10. 03	CNU8234	BARCPU15	MW315338	-	-
	2018. 10. 03	CNU8235	BARCPU02	MW315339	-	-
	2018. 06. 08	CNU8197	BARCPI06	MW315340	EF1ACPI12	MW348841
	2018. 06. 08	-	-	-	EF1ACPI12	MW348841
	2018. 06. 08	-	-	-	EF1ACPI12	MW348841
	2018. 06. 08	-	-	-	EF1ACPI12	MW348841
	2018. 06. 08	-	-	-	EF1ACPI16	MW348842
	2019. 04. 12	CNU10681	BARCPI12	MW315341	EF1ACPI12	MW348843
	2019. 04. 12	-	-	-	EF1ACPI32	MW348844
	2019. 04. 12	-	-	-	EF1ACPI33	MW348845
	2019. 04. 12	-	-	-	EF1ACPI33	MW348845
	2019. 04. 12	-	-	-	EF1ACPI34	MW348846
	2018. 10. 03	CNU8236	BARCPU02	MW315342	-	-
	2018. 10. 03	CNU8237	BARCPU02	MW315343	-	-
	2012. 06. 20	CP4551	BARCPI01	MW315344	-	-
	2018. 06. 08	CNU8196	BARCPI01	MW315345	-	-
	2018. 06. 08	CNU8198	BARCPI05	MW315346	-	-
	2018. 06. 08	CNU8199	BARCPI01	MW315347	-	-
	2019. 04. 12	CNU10682	BARCPI01	MW315348	-	-
	2019. 04. 12	CNU10683	BARCPI01	MW315349	-	-
	2019. 04. 12	CNU11055	BARCPI04	MW315350	-	-
	2019. 04. 12	CNU11056	BARCPI01	MW315351	-	-
13. Sancheong, GN (1)	2016. 08. 31	CNU8486	BARCPI09	MW315352	PI6†	MW348847
	2016. 08. 31	-	-	-	PI6†	MW348847
	2016. 08. 31	-	-	-	EF1ACPI22	MW348848
	2016. 08. 31	-	-	-	EF1ACPI23	MW348849
	2016. 08. 31	-	-	-	EF1ACPI24	MW348850
14. Mt. Jayangsan, Haman; GN (1)	2016. 10. 06	CNU8484	BARCPI07	MW315353	PI4†	MW348851
	2016. 10. 06	-	-	-	PI5†	MW348852
	2016. 10. 06	-	-	-	PI5†	MW348852
	2016. 10. 06	-	-	-	EF1ACPI17	MW348853
	2016. 10. 06	-	-	-	EF1ACPI18	MW348854
15. Geoje, GN (6)	2018. 07. 09	CNU8171	BARCPU06	MW315354	EF1ACPU01	MW348855
	2018. 07. 09	-	-	-	EF1ACPU16	MW348856
	2018. 07. 09	-	-	-	EF1ACPU17	MW348857
	2018. 07. 09	-	-	-	EF1ACPU18	MW348858
	2018. 07. 09	-	-	-	EF1ACPU19	MW348859
	2018. 07. 09	CNU8173	BARCPU07	MW315355	PU3†	MW348860
	2018. 07. 09	-	-	-	PU3†	MW348860
	2018. 07. 09	-	-	-	EF1ACPU01	MW348861
	2018. 07. 09	-	-	-	EF1ACPU01	MW348861
	2018. 07. 09	-	-	-	EF1ACPU20	MW348862
	2018. 07. 09	CNU8172	BARCPU01	MW315356	-	-
	2018. 10. 07	CNU8224	BARCPU02	MW315357	-	-
	2012. 06. 29	CP4555	BARCPI05	MW315358	EF1ACPI12	MW348863
	2012. 06. 29	-	-	-	EF1ACPI12	MW348863
	2012. 06. 29	-	-	-	EF1ACPI14	MW348864
	2012. 06. 29	-	-	-	EF1ACPI15	MW348865
	2012. 06. 29	-	-	-	PI3†	MW348866
	2012. 06. 29	CP4554	BARCPI04	MW315359	-	-
16. Changwon, GN (4)	2018. 10. 05	CNU8220	BARCPU02	MW315360	-	-
	2018. 10. 05	CNU8221	BARCPU13	MW315361	-	-
	2018. 10. 05	CNU8222	BARCPU01	MW315362	-	-
	2018. 10. 05	CNU8223	BARCPU02	MW315363	-	-
17. Mt. Wonhyosan, Yangsan; GN (1)	2016. 06. 30	CNU8485	BARCPI08	MW315364	EF1ACPI12	MW348867
	2016. 06. 30	-	-	-	EF1ACPI19	MW348868
	2016. 06. 30	-	-	-	EF1ACPI20	MW348869
	2016. 06. 30	-	-	-	EF1ACPI20	MW348869
	2016. 06. 30	-	-	-	EF1ACPI20	MW348869
	2016. 06. 30	-	-	-	EF1ACPI21	MW348870
	2016. 06. 30	-	-	-	EF1ACPI38	MW348871
	2016. 06. 30	-	-	-	EF1ACPI39	MW348872
	2016. 06. 30	-	-	-	EF1ACPI39	MW348872
	2016. 06. 30	-	-	-	EF1ACPI40	MW348873
	2016. 06. 30	-	-	-	EF1ACPI41	MW348874

	2016. 06. 30	-	-	-	EF1ACPI42	MW348875
	2016. 06. 30	-	-	-	EF1ACPI42	MW348875
18. Mt. Hwawangsan, Changnyeong; GN (1)	2014. 06. 26	CNU8487	BARCPI09	MW315365	-	-
19. Cheongdo, GB (4)	2018. 07. 09	CNU8175	BARCPU08	MW315366	EF1ACPU01	MW348876
	2018. 07. 09	-	-	-	EF1ACPU01	MW348876
	2018. 07. 09	-	-	-	EF1ACPU01	MW348876
	2018. 07. 09	-	-	-	EF1ACPU01	MW348876
	2018. 07. 09	-	-	-	EF1ACPU01	MW348876
	2018. 07. 09	-	-	-	EF1ACPU01	MW348876
	2018. 07. 09	-	-	-	EF1ACPU22	MW348877
	2018. 07. 09	-	-	-	EF1ACPU23	MW348878
	2018. 07. 09	-	-	-	EF1ACPU54	MW348879
	2018. 07. 09	-	-	-	EF1ACPU54	MW348879
	2018. 07. 09	-	-	-	EF1ACPU55	MW348880
	2018. 07. 09	-	-	-	EF1ACPU55	MW348880
	2018. 07. 09	-	-	-	PU4†	MW348881
	2018. 07. 09	-	-	-	PU4†	MW348881
	2018. 07. 09	CNU8176	BARCPU01	MW315367	-	-
	2018. 07. 09	CNU8177	BARCPU01	MW315368	-	-
	2018. 07. 09	CNU8174	BARCPU01	MW315369	-	-
20. Gyeongju, GB (2)	2012. 06. 25	CP4552	BARCPI02	MW315370	PI1†	MW348882
	2012. 06. 25	-	-	-	PI2†	MW348883
	2012. 06. 25	-	-	-	EF1ACPI05	MW348884
	2012. 06. 25	-	-	-	EF1ACPI06	MW348885
	2012. 06. 25	-	-	-	EF1ACPI07	MW348886
	2012. 06. 26	CP4553	BARCPI03	MW315371	EF1ACPI08	MW348887
	2012. 06. 26	-	-	-	EF1ACPI09	MW348888
	2012. 06. 26	-	-	-	EF1ACPI11	MW348889
	2012. 06. 26	-	-	-	EF1ACPI12	MW348890
21. Jeju, JP (2)	2018. 06. 15	CNU8207	BARCPU12	MW315372	EF1ACPU01	MW348891
	2018. 06. 15	-	-	-	EF1ACPU01	MW348891
	2018. 06. 15	-	-	-	EF1ACPU34	MW348892
	2018. 06. 15	-	-	-	EF1ACPU35	MW348893
	2018. 06. 15	-	-	-	EF1ACPU36	MW348894
	2018. 06. 28	CNU8205	BARCPU02	MW315373	-	-

GG, Gyeonggido Province; GW, Gangwondo Province; JB, Jeollabukdo Province; JN, Jeollanamdo Province; GB, Gyeongsangbukdo Province; GN, Gyeongsangnamdo Province; and JP, Jejudo Province. Numbers in parentheses indicate the sample size. Names starting with BARCPU indicate *COI* haplotypes of *C. punctiferalis*, whereas those starting with BARCPI indicate *COI* haplotypes of *C. pinicolalis*; †, untranslated haplotypes; -, individuals that were not sequenced for *COI* and *EF1α*.

Table S2. List of *COI* sequences of *Conogethes* species retrieved from GenBank and BOLD Systems

	Species	Locality	Length (bp)	Haplotype	GenBank Accession no.	BOLD Bin no.	References
1	*C. punctiferalis*	Korea	658	BARCPU01	KC136063	BOLD:AAC8071	Unpublished
2	*C. punctiferalis*	Korea	658	BARCPU01	KC136064	BOLD:AAC8071	Unpublished
3	*C. punctiferalis*	Korea	658	BARCPU01	KC136065	BOLD:AAC8071	Unpublished
4	*C. punctiferalis*	Korea	657	BARCPU16	JN087376	BOLD:AAC8071	Unpublished
5	*C. punctiferalis*	Korea	657	BARCPU01	MN609051	BOLD:AAC8071	Unpublished
6	*C. punctiferalis*	Korea	657	BARCPU02	MN609052	BOLD:AAC8071	Unpublished
7	*C. punctiferalis*	China	658	BARCPU17	MG954425	-	Unpublished
8	*C. punctiferalis*	China	658	BARCPU18	MG954426	-	Unpublished
9	*C. punctiferalis*	China	15,325	BARCPU02	KX150457	-	Unpublished
10	*C. punctiferalis*	China	15,355	BARCPU10	JX448619	BOLD:AAC8071	[1]
11	*C. punctiferalis*	Pakistan	658	BARCPU19	KX862984	BOLD:AAC8071	[2]
12	*C. punctiferalis*	Pakistan	658	BARCPU19	KX860179	BOLD:AAC8071	[2]
13	*C. punctiferalis*	Pakistan	658	BARCPU20	JF857967	BOLD:AAC8071	Unpublished
14	*C. punctiferalis*	Pakistan	677	BARCPU21	MK301225	BOLD:AAC8071	Unpublished
15	*C. punctiferalis*	Australia	658	BARCPU22	HQ953119	BOLD:AAD8750	Unpublished
16	*C. punctiferalis*	Australia	658	BARCPU22	HQ953121	BOLD:AAD8750	Unpublished
17	*C. punctiferalis*	Australia	658	BARCPU23	HQ953122	BOLD:AAD8750	Unpublished
18	*C. punctiferalis*	Australia	658	BARCPU22	HQ953124	BOLD:AAD8750	Unpublished
19	*C. punctiferalis*	Australia	658	BARCPU24	HQ953125	BOLD:AAD8750	Unpublished
20	*C. punctiferalis*	Australia	658	BARCPU25	HQ953126	BOLD:AAD8750	Unpublished
21	*C. punctiferalis*	Australia	658	BARCPU22	-	BOLD:AAD8750	Unpublished
22	*C. punctiferalis*	Australia	658	BARCPU26	-	BOLD:AAD8750	Unpublished
23	*C. punctiferalis*	Australia	658	BARCPU27	-	BOLD:AAD8750	Unpublished
24	*C. punctiferalis*	Australia	658	BARCPU25	-	BOLD:AAD8750	Unpublished
25	*C. punctiferalis*	Australia	658	BARCPU22	-	BOLD:AAD8750	Unpublished
26	*C. punctiferalis*	Australia	658	BARCPU28	-	BOLD:AAD8750	Unpublished
27	*C. punctiferalis*	Australia	658	BARCPU29	-	BOLD:AAD8750	Unpublished
28	*C. punctiferalis*	Australia	658	BARCPU22	-	BOLD:AAD8750	Unpublished
29	*C. punctiferalis*	Australia	658	BARCPU30	-	BOLD:AAD8750	Unpublished
30	*C. punctiferalis*	Australia	658	BARCPU31	-	BOLD:AAD8750	Unpublished
31	*C. punctiferalis*	Australia	658	BARCPU22	-	BOLD:AAD8750	Unpublished
32	*C. punctiferalis*	Australia	658	BARCPU25	-	BOLD:AAD8750	Unpublished
33	*C. punctiferalis*	Japan	1,522	BARCPU32	AB751251	BOLD:AAC8071	Unpublished
34	*C. punctiferalis*	Thailand	658	BARCPU02	-	BOLD:AAC8071	Unpublished
35	*C. punctiferalis*	Thailand	658	BARCPU33	-	BOLD:AAC8071	Unpublished
36	*C. pluto*	Papua New Guinea	658	BARCPL01	KY323303	BOLD:AAE1679	[3]
37	*C. pluto*	Papua New Guinea	658	BARCPL01	KY323373	BOLD:AAE1679	[3]
38	*C. pluto*	Papua New Guinea	658	BARCPL01	KY323156	BOLD:AAE1679	[3]
39	*C. pluto*	Papua New Guinea	658	BARCPL01	KY323116	BOLD:AAE1679	[3]
40	*C. pluto*	Papua New Guinea	658	BARCPL01	KY323091	BOLD:AAE1679	[3]
41	*C. pluto*	Papua New Guinea	658	BARCPL01	GU695720	BOLD:AAE1679	Unpublished
42	*C. pluto*	Australia	658	BARCPL01	HQ953136	BOLD:AAE1679	Unpublished
43	*C. pluto*	Australia	658	BARCPL01	HQ953110	BOLD:AAE1679	Unpublished
44	*C. pluto*	Australia	658	BARCPL01	HQ953116	BOLD:AAE1679	Unpublished
45	*C. pluto*	Australia	658	BARCPL01	HQ953118	BOLD:AAE1679	Unpublished
46	*C. pluto*	Australia	658	BARCPL02	HQ953117	BOLD:AAE1679	Unpublished
47	*C. pluto*	Australia	658	BARCPL02	HQ953134	BOLD:AAE1679	Unpublished
48	*C. pluto*	Australia	658	BARCPL02	HQ953135	BOLD:AAE1679	Unpublished
49	*C. pluto*	Australia	658	BARCPL03	-	BOLD:AAE1679	Unpublished
50	*C. pluto*	Australia	658	BARCPL04	-	BOLD:AAE1679	Unpublished
51	*C. pluto*	Australia	658	BARCPL03	-	BOLD:AAE1679	Unpublished
52	*C. pluto*	Australia	658	BARCPL05	-	BOLD:AAE1679	Unpublished
53	*C. semifascialis*	Papua New Guinea	658	BARCSE01	KY323243	BOLD:AAF1272	[3]
54	*C. semifascialis*	Papua New Guinea	658	BARCSE02	KY323190	BOLD:AAF1272	[3]
55	*C. semifascialis*	Papua New Guinea	658	BARCSE01	KY323309	BOLD:AAF1272	[3]
56	*C. semifascialis*	Papua New Guinea	658	BARCSE03	GU695721	BOLD:AAF1272	Unpublished
57	*C. semifascialis*	Papua New Guinea	658	BARCSE01	-	BOLD:AAF1272	Unpublished
58	*C. semifascialis*	Australia	658	BARCSE04	HQ953123	BOLD:AAF1272	Unpublished
59	*C. semifascialis*	Australia	658	BARCSE05	HQ953120	BOLD:AAF1272	Unpublished
60	*C. semifascialis*	Australia	658	BARCSE06	KF392629	BOLD:ABZ6085	[4]
61	*C. semifascialis*	Australia	658	BARCSE07	-	BOLD:ABZ6085	Unpublished
62	*C. semifascialis*	Australia	658	BARCSE08	-	BOLD:ABZ6085	Unpublished
63	*C. semifascialis*	Australia	658	BARCSE07	-	BOLD:ABZ6085	Unpublished
64	*C. semifascialis*	Australia	658	BARCSE08	-	BOLD:ABZ6085	Unpublished
65	*C. tharsalea*	Australia	658	BARCTH01	HQ953137	BOLD:AAJ7249	Unpublished
66	*C. tharsalea*	Australia	658	BARCTH02	HQ953138	BOLD:AAJ7249	Unpublished
67	*C. tharsalea*	Australia	658	BARCTH03	HQ953139	BOLD:AAJ7249	Unpublished
68	*C. tharsalea*	Australia	658	BARCTH04	-	BOLD:AAJ7249	Unpublished
69	*C. ersealis*	Australia	658	BARCER01	HQ953132	BOLD:AAO6794	Unpublished

70	*C. ersealis*	Australia	658	BARCER02	HQ953133	BOLD:AAO6794	Unpublished
71	*C. diminutiva*	Australia	658	BARCDI01	HQ953130	BOLD:AAP0131	Unpublished
72	*C. haemactalis*	Australia	658	BARCHA01	HQ953127	BOLD:AAO4737	Unpublished
73	*C. haemactalis*	Australia	658	BARCHA01	HQ953128	BOLD:AAO4737	Unpublished
74	*C. haemactalis*	Australia	658	BARCHA01	HQ953129	BOLD:AAO4737	Unpublished
75	*C. haemactalis*	Australia	658	BARCHA01	-	BOLD:AAO4737	Unpublished
76	*C. evaxalis*	Australia	658	BARCEV01	HQ953111	BOLD:ABZ8282	Unpublished
77	*C. evaxalis*	Australia	658	BARCEV01	HQ953114	BOLD:ABZ8282	Unpublished
78	*C. evaxalis*	Australia	658	BARCEV02	HQ953115	BOLD:ABZ8282	Unpublished
79	*C. evaxalis*	Papua New Guinea	658	BARCEV03	JX970234	BOLD:AAF1273	Unpublished
80	*C. evaxalis*	Papua New Guinea	658	BARCEV03	JX970235	BOLD:AAF1273	Unpublished
81	*C. pandamalis*	Malaysia	1,438	BARCPA01	MK459688	BOLD:AEA7122	[5]

-, not available.

Table S3. Pupal period and lifespan of *Conogethes pinicolalis* (days)

	Pupae (no. of individuals)	Adult (no. of individuals)
Mean ± S.D.	12.02 ± 1.24 (46)	8.05 ± 5.09 (20)

Values indicate the mean ± S.D. for each stage of *C. pinicolalis* development.

Table S4. Pairwise comparisons of *COI* haplotypes of *Conogethes* species

		1	2	3	4	5	6	7	8	9	10	11	12	13	14	15	16	17	18	19	20	21	22	23	24	25
1	*C. punctiferalis* (Current study, BARCPU01)	-	0.92	0.76	1.07	1.07	0.76	0.76	1.07	1.07	0.76	1.37	0.15	1.22	1.07	1.07	1.07	0.92	1.07	0.15	1.07	1.07	5.04	5.19	4.89	5.04
2	*C. punctiferalis* (Current study, China, Thailand BARCPU02)	6	-	0.15	0.46	0.15	0.15	0.31	0.15	0.15	0.15	0.76	1.07	0.31	0.15	0.15	0.15	0.31	0.15	0.76	0.15	0.15	5.34	5.50	5.19	5.34
3	*C. punctiferalis* (Current study, BARCPU03)	5	1	-	0.31	0.31	0.31	0.15	0.31	0.31	0.31	0.92	0.92	0.46	0.31	0.31	0.31	0.15	0.31	0.92	0.31	0.31	5.50	5.65	5.34	5.50
4	*C. punctiferalis* (Current study, BARCPU04)	7	3	2	-	0.61	0.61	0.46	0.61	0.61	0.61	1.22	1.22	0.76	0.61	0.61	0.61	0.46	0.61	1.22	0.61	0.61	5.19	5.34	5.04	5.19
5	*C. punctiferalis* (Current study, BARCPU05)	7	1	2	4	-	0.31	0.46	0.31	0.31	0.31	0.92	1.22	0.46	0.31	0.31	0.31	0.46	0.31	0.92	0.31	0.31	5.50	5.65	5.34	5.50
6	*C. punctiferalis* (Current study, BARCPU06)	5	1	2	4	2	-	0.46	0.31	0.31	0.31	0.92	0.92	0.46	0.31	0.31	0.31	0.46	0.31	0.61	0.31	0.31	5.34	5.50	5.19	5.34
7	*C. punctiferalis* (Current study, BARCPU07)	5	2	1	3	3	3	-	0.46	0.46	0.46	1.07	0.92	0.61	0.46	0.46	0.46	0.31	0.46	0.92	0.46	0.46	5.50	5.65	5.34	5.50
8	*C. punctiferalis* (Current study, BARCPU08)	7	1	2	4	2	2	3	-	0.31	0.31	0.92	1.22	0.46	0.31	0.31	0.31	0.46	0.31	0.92	0.31	0.31	5.50	5.65	5.34	5.50
9	*C. punctiferalis* (Current study, BARCPU09)	7	1	2	4	2	2	3	2	-	0.31	0.92	0.92	0.15	0.31	0.31	0.31	0.46	0.31	0.92	0.31	0.31	5.19	5.34	5.04	5.19
10	*C. punctiferalis* (Current study, China, BARCPU10)	5	1	2	4	2	2	3	2	2	-	0.61	0.92	0.46	0.31	0.31	0.31	0.46	0.31	0.61	0.31	0.31	5.19	5.34	5.04	5.19
11	*C. punctiferalis* (Current study, BARCPU11)	9	5	6	8	6	6	7	6	6	4	-	1.53	1.07	0.92	0.92	0.92	1.07	0.92	1.22	0.92	0.92	5.50	5.65	5.34	5.50
12	*C. punctiferalis* (Current study, BARCPU12)	1	7	6	8	8	6	6	8	6	6	10	-	1.07	1.22	1.22	1.22	1.07	1.22	0.31	1.22	1.22	4.89	5.04	4.73	4.89
13	*C. punctiferalis* (Current study, BARCPU13)	8	2	3	5	3	3	4	3	1	3	7	7	-	0.46	0.46	0.15	0.61	0.46	1.07	0.46	0.46	5.34	5.50	5.19	5.34
14	*C. punctiferalis* (Current study, BARCPU14)	7	1	2	4	2	2	3	2	2	2	6	8	3	-	0.31	0.31	0.46	0.31	0.92	0.31	0.31	5.50	5.65	5.34	5.50
15	*C. punctiferalis* (Current study, BARCPU15)	7	1	2	4	2	2	3	2	2	2	6	8	3	2	-	0.31	0.46	0.31	0.92	0.31	0.31	5.50	5.65	5.34	5.50
16	*C. punctiferalis* (Korea, BARCPU16)	7	1	2	4	2	2	3	2	2	2	6	8	1	2	2	-	0.46	0.31	0.92	0.31	0.31	5.50	5.65	5.34	5.50
17	*C. punctiferalis* (China, BARCPU17)	6	2	1	3	3	3	2	3	3	3	7	7	4	3	3	3	-	0.46	1.07	0.46	0.46	5.65	5.80	5.50	5.65
18	*C. punctiferalis* (China, BARCPU18)	7	1	2	4	2	2	3	2	2	2	6	8	3	2	2	2	3	-	0.92	0.31	0.31	5.50	5.65	5.34	5.50
19	*C. punctiferalis* (Pakistan, BARCPU19)	1	5	6	8	6	4	6	6	6	4	8	2	7	6	6	6	7	6	-	0.92	0.92	4.89	5.04	4.73	4.89
20	*C. punctiferalis* (Pakistan, BARCPU20)	7	1	2	4	2	2	3	2	2	2	6	8	3	2	2	2	3	2	6	-	0.31	5.50	5.65	5.34	5.50
21	*C. punctiferalis* (Pakistan, BARCPU21)	7	1	2	4	2	2	3	2	2	2	6	8	3	2	2	2	3	2	6	2	-	5.50	5.65	5.34	5.50
22	*C. punctiferalis* (Australia, BARCPU22)	33	35	36	34	36	35	36	36	34	34	36	32	35	36	36	36	37	36	32	36	36	-	0.15	0.15	0.31
23	*C. punctiferalis* (Australia, BARCPU23)	34	36	37	35	37	36	37	37	35	35	37	33	36	37	37	37	38	37	33	37	37	1	-	0.31	0.46
24	*C. punctiferalis* (Australia, BARCPU24)	32	34	35	33	35	34	35	35	33	33	35	31	34	35	35	35	36	35	31	35	35	1	2	-	0.46
25	*C. punctiferalis* (Australia, BARCPU25)	33	35	36	34	36	35	36	36	34	34	36	32	35	36	36	36	37	36	32	36	36	2	3	3	-
26	*C. punctiferalis* (Australia, BARCPU26)	34	36	37	35	37	36	37	35	35	35	37	33	36	37	37	37	38	37	33	37	37	1	2	2	3
27	*C. punctiferalis* (Australia, BARCPU27)	16	17	16	16	18	16	17	18	16	16	20	15	17	18	18	18	17	18	17	18	16	29	30	28	29
28	*C. punctiferalis* (Australia, BARCPU28)	32	34	35	33	35	34	35	35	33	33	35	31	34	35	35	35	36	35	31	35	35	1	2	2	3
29	*C. punctiferalis* (Australia, BARCPU29)	32	34	35	33	35	34	35	35	33	33	35	31	34	35	35	35	36	35	31	35	35	1	2	2	3
30	*C. punctiferalis* (Australia, BARCPU30)	34	36	37	35	37	36	37	37	35	35	37	33	36	37	37	37	38	37	33	37	37	1	2	2	3
31	*C. punctiferalis* (Australia, BARCPU31)	15	16	15	15	17	15	16	17	15	15	19	14	16	17	17	17	16	17	16	17	15	28	29	27	28
32	*C. punctiferalis* (Japan, BARCPU32)	7	1	2	4	2	2	3	2	2	2	6	8	3	2	2	2	3	2	6	2	2	36	37	35	36
33	*C. punctiferalis* (Thailand, BARCPU33)	6	2	3	3	3	3	4	3	3	1	5	7	4	3	3	3	4	3	5	3	3	33	34	32	33
34	*C. pinicolalis* (Current study, BARCPI01)	34	35	34	33	36	36	35	34	34	34	38	33	35	36	36	36	33	36	35	36	36	44	45	43	44
35	*C. pinicolalis* (Current study, BARCPI02)	34	37	36	35	38	36	37	36	36	36	40	33	37	38	38	38	35	38	35	38	38	45	46	44	45
36	*C. pinicolalis* (Current study, BARCPI03)	34	35	36	35	34	36	36	36	34	34	38	33	35	36	36	36	35	36	33	36	36	42	43	41	42
37	*C. pinicolalis* (Current study, BARCPI04)	34	37	36	35	38	38	36	36	36	36	40	33	37	38	38	38	35	38	35	38	38	44	45	43	44
38	*C. pinicolalis* (Current study, BARCPI05)	35	36	35	34	37	37	36	35	35	35	39	34	36	37	37	37	34	37	36	37	37	45	46	44	45
39	*C. pinicolalis* (Current study, BARCPI06)	33	34	33	32	35	35	34	33	33	33	37	32	34	35	35	35	32	35	34	35	35	43	44	42	43
40	*C. pinicolalis* (Current study, BARCPI07)	35	36	35	34	37	37	36	35	35	35	39	34	36	37	37	37	34	37	36	37	37	45	46	44	45
41	*C. pinicolalis* (Current study, BARCPI08)	34	35	36	35	36	34	37	34	34	34	38	33	35	36	36	36	35	36	33	36	36	43	44	42	43
42	*C. pinicolalis* (Current study, BARCPI09)	37	38	37	36	39	39	38	37	37	37	41	36	38	39	39	39	36	39	38	39	39	44	45	43	44
43	*C. pinicolalis* (Current study, BARCPI10)	34	35	34	33	36	36	35	34	34	34	38	33	35	36	36	36	33	36	35	36	36	44	45	43	44
44	*C. pinicolalis* (Current study, BARCPI11)	33	36	35	34	37	37	35	35	35	35	39	32	36	37	37	37	34	37	34	37	37	45	46	44	45
45	*C. pinicolalis* (Current study, BARCPI12)	35	36	35	34	37	37	36	35	35	35	39	34	36	37	37	37	34	37	36	37	37	45	46	44	45
46	*C. pluto* (Papua New Guinea, Autralia, BARCPL01)	37	40	41	39	41	39	41	41	39	39	43	36	40	41	41	41	42	41	36	41	41	40	41	41	40
47	*C. pluto* (Australia, BARCPL02)	38	41	42	40	42	40	42	42	40	40	44	37	41	42	42	42	43	42	37	42	42	41	42	42	41
48	*C. pluto* (Australia, BARCPL03)	36	39	40	38	40	38	40	40	38	38	42	35	39	40	40	40	41	40	35	40	40	41	42	42	41
49	*C. pluto* (Australia, BARCPL04)	37	40	41	39	41	39	41	41	39	39	43	36	40	41	41	41	42	41	36	41	41	40	41	41	40
50	*C. pluto* (Australia, BARCPL05)	38	41	42	40	42	40	42	42	40	40	44	37	41	42	42	42	43	42	37	42	42	39	40	40	39
51	*C. semifascialis* (Papua New Guinea, BARCSE01)	34	35	35	33	36	35	36	36	34	34	36	33	35	36	36	36	34	36	34	36	36	25	26	26	25
52	*C. semifascialis* (Papua New Guinea, BARCSE02)	36	37	37	35	38	37	38	38	36	36	38	35	37	38	38	38	36	38	36	38	38	25	26	26	25
53	*C. semifascialis* (Papua New Guinea, BARCSE03)	36	37	37	35	38	37	38	38	36	36	38	35	37	38	38	38	36	38	36	38	38	25	26	26	25
54	*C. semifascialis* (Australia, BARCSE04)	35	36	36	34	37	36	37	37	35	35	37	34	36	37	37	37	35	37	35	37	37	26	27	27	26
55	*C. semifascialis* (Australia, BARCSE05)	35	36	36	34	37	36	37	37	35	35	37	34	36	37	37	37	35	37	35	37	37	28	29	29	28
56	*C. semifascialis* (Australia, BARCSE06)	16	17	16	16	18	16	17	18	16	16	20	15	17	18	18	18	17	18	17	18	16	29	30	28	29
57	*C. semifascialis* (Australia, BARCSE07)	15	16	15	15	17	15	16	17	15	15	19	14	16	17	17	17	16	17	16	17	15	30	31	29	30
58	*C. semifascialis* (Australia, BARCSE08)	14	15	14	14	16	14	15	16	14	14	18	13	15	16	16	16	15	16	15	16	14	29	30	28	29
59	*C. tharsalea* (Australia, BARCTH01)	66	63	64	64	63	63	65	64	62	62	60	65	63	64	64	64	65	62	65	64	64	66	67	66	66
60	*C. tharsalea* (Australia, BARCTH02)	69	66	67	67	66	66	68	67	65	65	63	68	66	67	67	67	68	65	68	67	67	69	70	69	69
61	*C. tharsalea* (Australia, BARCTH03)	68	65	66	66	65	65	67	66	64	64	62	67	65	66	66	66	67	64	67	66	66	68	69	68	68
62	*C. tharsalea* (Australia, BARCTH04)	67	64	65	65	64	64	66	65	63	63	61	66	64	65	65	65	66	63	66	65	65	67	68	67	67

63	*C. ersealis* (Australia, BARCER01)	51	53	54	53	54	53	54	54	52	52	56	50	53	54	54	54	55	52	50	54	52	48	49	49	48
64	*C. ersealis* (Australia, BARCER02)	49	51	52	51	52	51	52	52	50	50	54	48	51	52	52	52	53	50	48	52	50	46	47	47	46
65	*C. diminutiva* (Australia, BARCDI01)	51	55	54	53	54	55	54	56	54	54	58	50	55	56	56	56	55	54	52	56	56	56	57	57	56
66	*C. haemactalis* (Australia, BARCHA01)	63	63	63	61	63	64	64	64	62	62	66	62	63	64	64	64	64	62	63	64	62	63	64	64	65
67	*C. evaxalis* (Australia, BARCEV01)	57	59	59	58	60	58	59	60	58	58	62	56	59	60	60	60	60	60	57	60	58	61	62	60	61
68	*C. evaxalis* (Australia, BARCEV02)	56	58	58	57	59	57	58	59	57	57	61	55	58	59	59	59	59	59	56	59	57	62	63	61	62
69	*C. evaxalis* (Australia, BARCEV03)	52	52	52	51	53	51	53	53	51	51	55	51	52	53	53	53	53	53	52	53	51	66	67	65	66
70	*C. pandamalis* (Malaysia, BARCPA01)	38	39	38	38	38	40	38	38	38	38	42	37	39	40	40	40	39	40	39	40	38	46	47	47	46

		26	27	28	29	30	31	32	33	34	35	36	37	38	39	40	41	42	43	44	45	46	47	48	49	50
1	*C. punctiferalis* (Current study, BARCPU01)	5.19	2.44	4.89	4.89	5.19	2.29	1.07	0.92	5.19	5.19	5.19	5.19	5.34	5.04	5.34	5.19	5.65	5.19	5.04	5.34	5.65	5.80	5.50	5.65	5.80
2	*C. punctiferalis* (Current study, China, Thailand BARCPU02)	5.50	2.60	5.19	5.19	5.50	2.44	0.15	0.31	5.34	5.65	5.34	5.65	5.50	5.19	5.50	5.34	5.80	5.34	5.50	5.50	6.11	6.26	5.95	6.11	6.26
3	*C. punctiferalis* (Current study, BARCPU03)	5.65	2.44	5.34	5.34	5.65	2.29	0.31	0.46	5.19	5.50	5.50	5.50	5.34	5.04	5.34	5.50	5.65	5.19	5.34	5.34	6.26	6.41	6.11	6.26	6.41
4	*C. punctiferalis* (Current study, BARCPU04)	5.34	2.44	5.04	5.04	5.34	2.29	0.61	0.46	5.04	5.34	5.34	5.34	5.19	4.89	5.19	5.34	5.50	5.04	5.19	5.19	5.95	6.11	5.80	5.95	6.11
5	*C. punctiferalis* (Current study, BARCPU05)	5.65	2.75	5.34	5.34	5.65	2.60	0.31	0.46	5.50	5.80	5.19	5.80	5.65	5.34	5.65	5.50	5.95	5.50	5.65	5.65	6.26	6.41	6.11	6.26	6.41
6	*C. punctiferalis* (Current study, BARCPU06)	5.50	2.44	5.19	5.19	5.50	2.29	0.31	0.46	5.50	5.50	5.50	5.80	5.65	5.34	5.65	5.19	5.95	5.50	5.65	5.65	5.95	6.11	5.80	5.95	6.11
7	*C. punctiferalis* (Current study, BARCPU07)	5.65	2.60	5.34	5.34	5.65	2.44	0.46	0.61	5.34	5.65	5.50	5.50	5.50	5.19	5.50	5.65	5.80	5.34	5.34	5.50	6.26	6.41	6.11	6.26	6.41
8	*C. punctiferalis* (Current study, BARCPU08)	5.34	2.75	5.34	5.34	5.65	2.60	0.31	0.46	5.19	5.50	5.50	5.50	5.34	5.04	5.34	5.19	5.65	5.19	5.34	5.34	6.26	6.41	6.11	6.26	6.41
9	*C. punctiferalis* (Current study, BARCPU09)	5.34	2.44	5.04	5.04	5.34	2.29	0.31	0.46	5.19	5.50	5.19	5.50	5.34	5.04	5.34	5.19	5.65	5.19	5.34	5.34	5.95	6.11	5.80	5.95	6.11
10	*C. punctiferalis* (Current study, China, BARCPU10)	5.34	2.44	5.04	5.04	5.34	2.29	0.31	0.15	5.19	5.50	5.19	5.50	5.34	5.04	5.34	5.19	5.65	5.19	5.34	5.34	5.95	6.11	5.80	5.95	6.11
11	*C. punctiferalis* (Current study, BARCPU11)	5.65	3.05	5.34	5.34	5.65	2.90	0.92	0.76	5.80	6.11	5.80	6.11	5.95	5.65	5.95	5.80	6.26	5.80	5.95	5.95	6.57	6.72	6.41	6.57	6.72
12	*C. punctiferalis* (Current study, BARCPU12)	5.04	2.29	4.73	4.73	5.04	2.14	1.22	1.07	5.04	5.04	5.04	5.04	5.19	4.89	5.19	5.04	5.50	5.04	4.89	5.19	5.50	5.65	5.34	5.50	5.65
13	*C. punctiferalis* (Current study, BARCPU13)	5.50	2.60	5.19	5.19	5.50	2.44	0.46	0.61	5.34	5.65	5.34	5.65	5.50	5.19	5.50	5.34	5.80	5.34	5.50	5.50	6.11	6.26	5.95	6.11	6.26
14	*C. punctiferalis* (Current study, BARCPU14)	5.65	2.75	5.34	5.34	5.65	2.60	0.31	0.46	5.50	5.80	5.50	5.80	5.65	5.34	5.65	5.50	5.95	5.50	5.65	5.65	6.26	6.41	6.11	6.26	6.41
15	*C. punctiferalis* (Current study, BARCPU15)	5.65	2.75	5.34	5.34	5.65	2.60	0.31	0.46	5.50	5.80	5.50	5.80	5.65	5.34	5.65	5.50	5.95	5.50	5.65	5.65	6.26	6.41	6.11	6.26	6.41
16	*C. punctiferalis* (Korea, BARCPU16)	5.65	2.75	5.34	5.34	5.65	2.60	0.31	0.46	5.50	5.80	5.50	5.80	5.65	5.34	5.65	5.50	5.95	5.50	5.65	5.65	6.26	6.41	6.11	6.26	6.41
17	*C. punctiferalis* (China, BARCPU17)	5.80	2.60	5.50	5.50	5.80	2.44	0.46	0.61	5.04	5.34	5.34	5.34	5.19	4.89	5.19	5.34	5.50	5.04	5.19	5.19	6.41	6.57	6.26	6.41	6.57
18	*C. punctiferalis* (China, BARCPU18)	5.65	2.75	5.34	5.34	5.65	2.60	0.31	0.46	5.50	5.80	5.50	5.80	5.65	5.34	5.65	5.50	5.95	5.50	5.65	5.65	6.26	6.41	6.11	6.26	6.41
19	*C. punctiferalis* (Pakistan, BARCPU19)	5.04	2.60	4.73	4.73	5.04	2.44	0.92	0.76	5.34	5.34	5.04	5.34	5.50	5.19	5.50	5.04	5.80	5.34	5.19	5.50	5.50	5.65	5.34	5.50	5.65
20	*C. punctiferalis* (Pakistan, BARCPU20)	5.65	2.75	5.34	5.34	5.65	2.60	0.31	0.46	5.50	5.80	5.50	5.80	5.65	5.34	5.65	5.50	5.95	5.50	5.65	5.65	6.26	6.41	6.11	6.26	6.41
21	*C. punctiferalis* (Pakistan, BARCPU21)	5.65	2.44	5.34	5.34	5.65	2.29	0.31	0.46	5.50	5.80	5.50	5.80	5.65	5.34	5.65	5.50	5.95	5.50	5.65	5.65	6.26	6.41	6.11	6.26	6.41
22	*C. punctiferalis* (Australia, BARCPU22)	0.15	4.43	0.15	0.15	0.15	4.28	5.50	5.04	6.72	6.87	6.41	6.72	6.87	6.57	6.87	6.57	6.72	6.72	6.87	6.87	6.11	6.26	6.26	6.11	5.95
23	*C. punctiferalis* (Australia, BARCPU23)	0.31	4.58	0.31	0.31	0.31	4.43	5.65	5.19	6.87	7.02	6.57	6.87	7.02	6.72	7.02	6.72	6.87	6.87	7.02	7.02	6.26	6.41	6.41	6.26	6.11
24	*C. punctiferalis* (Australia, BARCPU24)	0.31	4.28	0.31	0.31	0.31	4.12	5.34	4.89	6.57	6.72	6.26	6.57	6.72	6.41	6.72	6.41	6.57	6.57	6.72	6.72	6.26	6.41	6.41	6.26	6.11
25	*C. punctiferalis* (Australia, BARCPU25)	0.46	4.43	0.46	0.46	0.46	4.28	5.50	5.04	6.72	6.87	6.41	6.72	6.87	6.57	6.87	6.57	6.72	6.72	6.87	6.87	6.11	6.26	6.26	6.11	5.95
26	*C. punctiferalis* (Australia, BARCPU26)	-	4.58	0.31	0.31	0.31	4.43	5.65	5.19	6.57	6.72	6.57	6.57	6.72	6.41	6.72	6.41	6.57	6.57	6.72	6.72	6.26	6.41	6.41	6.26	6.11
27	*C. punctiferalis* (Australia, BARCPU27)	30	-	4.28	4.28	4.58	0.15	2.75	2.60	5.50	5.50	5.50	5.80	5.65	5.34	5.65	5.50	5.65	5.50	5.65	5.65	6.26	6.41	6.11	6.26	6.11
28	*C. punctiferalis* (Australia, BARCPU28)	2	28	-	0.31	0.31	4.12	5.34	4.89	6.57	6.72	6.26	6.57	6.72	6.41	6.72	6.41	6.57	6.57	6.72	6.72	5.95	6.11	6.11	5.95	5.80
29	*C. punctiferalis* (Australia, BARCPU29)	2	28	2	-	0.31	4.12	5.34	4.89	6.57	6.72	6.26	6.57	6.72	6.41	6.72	6.41	6.57	6.57	6.72	6.72	6.26	6.41	6.41	6.26	6.11
30	*C. punctiferalis* (Australia, BARCPU30)	2	30	2	2	-	4.43	5.65	5.19	6.87	7.02	6.57	6.87	7.02	6.72	7.02	6.72	6.87	6.87	7.02	7.02	6.26	6.41	6.41	6.26	6.11
31	*C. punctiferalis* (Australia, BARCPU31)	29	1	27	27	29	-	2.60	2.44	5.34	5.34	5.34	5.65	5.50	5.19	5.50	5.34	5.50	5.34	5.50	5.50	6.11	6.26	5.95	6.11	5.95
32	*C. punctiferalis* (Japan, BARCPU32)	37	18	35	35	37	17	-	0.46	5.50	5.80	5.50	5.80	5.65	5.34	5.65	5.50	5.95	5.50	5.65	5.65	6.26	6.41	6.11	6.26	6.41
33	*C. punctiferalis* (Thailand, BARCPU33)	34	17	32	32	34	16	3	-	5.19	5.50	5.19	5.50	5.34	5.04	5.34	5.19	5.65	5.19	5.34	5.34	5.80	5.95	5.65	5.80	5.95
34	*C. pinicolalis* (Current study, BARCPI01)	43	36	43	43	45	35	36	34	-	0.31	1.22	0.31	0.15	0.15	0.15	0.31	0.46	0.31	0.46	0.15	6.57	6.72	6.41	6.57	6.72
35	*C. pinicolalis* (Current study, BARCPI02)	44	36	44	44	46	35	38	36	2	-	1.53	0.61	0.46	0.46	0.46	0.31	0.76	0.61	0.76	0.46	6.57	6.72	6.41	6.57	6.72
36	*C. pinicolalis* (Current study, BARCPI03)	43	36	41	41	43	35	36	34	8	10	-	1.22	1.37	1.37	1.37	1.22	1.68	1.53	1.37	1.37	6.11	6.26	5.95	6.11	6.26
37	*C. pinicolalis* (Current study, BARCPI04)	43	38	43	43	45	37	38	36	2	4	8	-	0.46	0.46	0.46	0.61	0.76	0.31	0.15	0.46	6.26	6.41	6.11	6.26	6.41
38	*C. pinicolalis* (Current study, BARCPI05)	44	37	44	44	46	36	37	35	1	3	9	3	-	0.31	0.31	0.46	0.61	0.46	0.61	0.31	6.72	6.87	6.57	6.72	6.87
39	*C. pinicolalis* (Current study, BARCPI06)	42	35	42	42	44	34	35	33	1	3	9	3	2	-	0.31	0.46	0.61	0.46	0.61	0.31	6.41	6.57	6.26	6.41	6.57
40	*C. pinicolalis* (Current study, BARCPI07)	44	37	44	44	46	36	37	35	1	3	9	3	2	2	-	0.46	0.61	0.46	0.61	0.31	6.72	6.87	6.57	6.72	6.87
41	*C. pinicolalis* (Current study, BARCPI08)	42	36	42	42	44	35	36	34	2	2	8	4	3	3	3	-	0.76	0.61	0.76	0.46	6.26	6.41	6.11	6.26	6.41
42	*C. pinicolalis* (Current study, BARCPI09)	43	37	43	43	45	36	39	37	3	5	11	5	4	4	4	5	-	0.76	0.92	0.61	6.72	6.87	6.57	6.72	6.87
43	*C. pinicolalis* (Current study, BARCPI10)	43	36	43	43	45	35	36	34	2	4	10	2	3	3	3	4	5	-	0.46	0.46	6.26	6.41	6.11	6.26	6.41
44	*C. pinicolalis* (Current study, BARCPI11)	44	37	44	44	46	36	37	35	3	5	9	1	4	4	4	5	6	3	-	0.61	6.41	6.57	6.26	6.41	6.57
45	*C. pinicolalis* (Current study, BARCPI12)	44	37	44	44	46	36	37	35	1	3	9	3	2	2	2	3	4	3	4	-	6.72	6.87	6.57	6.72	6.87
46	*C. pluto* (Papua New Guinea, Autralia, BARCPL01)	41	41	39	41	41	40	41	38	43	43	40	41	44	42	44	41	44	41	42	44	-	0.15	0.15	0.31	0.15
47	*C. pluto* (Australia, BARCPL02)	42	42	40	42	42	41	42	39	44	44	41	42	45	43	45	42	45	42	43	45	1	-	0.31	0.46	0.31

48	*C. pluto* (Australia, BARCPL03)	42	40	40	42	42	39	40	37	42	42	39	40	43	41	43	40	43	40	41	43	1	2	-	0.46	0.31
49	*C. pluto* (Australia, BARCPL04)	41	41	39	41	41	40	41	38	43	43	40	41	44	42	44	41	44	41	42	44	2	3	3	-	0.46
50	*C. pluto* (Australia, BARCPL05)	40	40	38	40	40	39	42	39	44	44	41	42	45	43	45	42	45	42	43	45	1	2	2	3	-
51	*C. semifascialis* (Papua New Guinea, BARCSE01)	26	32	24	24	26	31	36	33	39	40	40	41	40	38	40	39	42	39	42	40	44	45	43	43	43
52	*C. semifascialis* (Papua New Guinea, BARCSE02)	26	30	24	24	26	29	38	35	39	40	40	41	40	38	40	39	40	39	42	40	42	43	41	41	41
53	*C. semifascialis* (Papua New Guinea, BARCSE03)	26	34	24	24	26	33	38	35	39	40	40	41	40	38	40	39	42	39	42	40	44	45	43	43	43
54	*C. semifascialis* (Australia, BARCSE04)	27	33	25	25	27	32	37	34	40	41	41	42	41	39	41	40	43	40	43	41	43	44	42	42	42
55	*C. semifascialis* (Australia, BARCSE05)	29	33	27	27	29	32	37	34	40	41	41	42	41	39	41	40	43	40	43	41	43	44	42	42	42
56	*C. semifascialis* (Australia, BARCSE06)	30	0	28	28	30	1	18	17	36	36	36	38	37	35	37	36	37	36	37	37	41	42	40	41	40
57	*C. semifascialis* (Australia, BARCSE07)	31	1	29	29	31	2	17	16	35	35	35	37	36	34	36	35	38	35	36	36	42	43	41	42	41
58	*C. semifascialis* (Australia, BARCSE08)	30	2	28	28	30	1	16	15	34	34	34	36	35	33	35	34	37	34	35	35	41	42	40	41	40
59	*C. tharsalea* (Australia, BARCTH01)	67	67	65	66	67	66	64	61	75	76	75	75	76	74	76	74	78	73	76	76	71	72	70	71	70
60	*C. tharsalea* (Australia, BARCTH02)	70	70	68	69	70	69	67	64	76	77	76	76	77	77	77	75	79	74	77	77	74	75	73	74	73
61	*C. tharsalea* (Australia, BARCTH03)	69	69	67	68	69	68	66	63	75	76	75	75	76	76	76	74	78	73	76	76	73	74	72	73	72
62	*C. tharsalea* (Australia, BARCTH04)	68	68	66	67	68	67	65	62	74	75	74	74	75	75	75	73	77	72	75	75	72	73	71	72	71
63	*C. ersealis* (Australia, BARCER01)	49	47	47	49	49	48	54	52	49	50	47	47	50	48	50	48	52	47	47	50	48	49	49	50	47
64	*C. ersealis* (Australia, BARCER02)	47	45	45	47	47	46	52	50	47	48	45	45	48	46	48	46	50	45	45	48	46	47	47	48	45
65	*C. diminutiva* (Australia, BARCDI01)	57	51	55	55	57	52	56	54	51	52	49	49	52	52	52	52	54	49	49	50	55	56	54	57	54
66	*C. haemactalis* (Australia, BARCHA01)	64	62	62	62	64	61	63	61	61	63	60	61	62	60	62	62	64	59	60	62	66	67	65	64	66
67	*C. evaxalis* (Australia, BARCEV01)	62	56	60	62	61	55	60	58	59	59	59	59	60	58	60	58	62	61	59	60	61	62	60	63	61
68	*C. evaxalis* (Australia, BARCEV02)	63	55	61	63	62	54	59	57	60	60	58	60	61	59	61	59	63	62	60	61	62	63	61	64	62
69	*C. evaxalis* (Australia, BARCEV03)	67	51	65	65	66	50	53	51	53	53	55	55	54	52	54	52	56	55	55	54	64	65	63	64	64
70	*C. pandamalis* (Malaysia, BARCPA01)	45	38	45	47	47	37	40	39	35	37	32	35	36	34	36	37	38	35	36	36	39	40	38	39	40

		51	52	53	54	55	56	57	58	59	60	61	62	63	64	65	66	67	68	69	70
1	*C. punctiferalis* (Current study, BARCPU01)	5.19	5.50	5.50	5.34	5.34	2.44	2.29	2.14	10.08	10.53	10.38	10.23	7.79	7.48	7.79	9.62	8.70	8.55	7.94	5.80
2	*C. punctiferalis* (Current study, China, Thailand BARCPU02)	5.34	5.65	5.65	5.50	5.50	2.60	2.44	2.29	9.62	10.08	9.92	9.77	8.09	7.79	8.40	9.62	9.01	8.86	7.94	5.95
3	*C. punctiferalis* (Current study, BARCPU03)	5.34	5.65	5.65	5.50	5.50	2.44	2.29	2.14	9.77	10.23	10.08	9.92	8.24	7.94	8.24	9.62	9.01	8.86	7.94	5.80
4	*C. punctiferalis* (Current study, BARCPU04)	5.04	5.34	5.34	5.19	5.19	2.44	2.29	2.14	9.77	10.23	10.08	9.92	8.09	7.79	8.09	9.31	8.86	8.70	7.79	5.80
5	*C. punctiferalis* (Current study, BARCPU05)	5.50	5.80	5.80	5.65	5.65	2.75	2.60	2.44	9.62	10.08	9.92	9.77	8.24	7.94	8.24	9.62	9.16	9.01	8.09	5.80
6	*C. punctiferalis* (Current study, BARCPU06)	5.34	5.65	5.65	5.50	5.50	2.44	2.29	2.14	9.62	10.08	9.92	9.77	8.09	7.79	8.40	9.77	8.86	8.70	7.79	6.11
7	*C. punctiferalis* (Current study, BARCPU07)	5.50	5.80	5.80	5.65	5.65	2.60	2.44	2.29	9.92	10.38	10.23	10.08	8.24	7.94	8.24	9.77	9.01	8.86	8.09	5.80
8	*C. punctiferalis* (Current study, BARCPU08)	5.50	5.80	5.80	5.65	5.65	2.75	2.60	2.44	9.77	10.23	10.08	9.92	8.24	7.94	8.55	9.77	9.16	9.01	8.09	5.80
9	*C. punctiferalis* (Current study, BARCPU09)	5.19	5.50	5.50	5.34	5.34	2.44	2.29	2.14	9.47	9.92	9.77	9.62	7.94	7.63	8.24	9.47	8.86	8.70	7.79	5.80
10	*C. punctiferalis* (Current study, China, BARCPU10)	5.19	5.50	5.50	5.34	5.34	2.44	2.29	2.14	9.47	9.92	9.77	9.62	7.94	7.63	8.24	9.47	8.86	8.70	7.79	5.80
11	*C. punctiferalis* (Current study, BARCPU11)	5.50	5.80	5.80	5.65	5.65	3.05	2.90	2.75	9.16	9.62	9.47	9.31	8.55	8.24	8.86	10.08	9.47	9.31	8.40	6.41
12	*C. punctiferalis* (Current study, BARCPU12)	5.04	5.34	5.34	5.19	5.19	2.29	2.14	1.99	9.92	10.38	10.23	10.08	7.63	7.33	7.63	9.47	8.55	8.40	7.79	5.65
13	*C. punctiferalis* (Current study, BARCPU13)	5.34	5.65	5.65	5.50	5.50	2.60	2.44	2.29	9.62	10.08	9.92	9.77	8.09	7.79	8.40	9.62	9.01	8.86	7.94	5.95
14	*C. punctiferalis* (Current study, BARCPU14)	5.50	5.80	5.80	5.65	5.65	2.75	2.60	2.44	9.77	10.23	10.08	9.92	8.24	7.94	8.55	9.77	9.16	9.01	8.09	6.11
15	*C. punctiferalis* (Current study, BARCPU15)	5.50	5.80	5.80	5.65	5.65	2.75	2.60	2.44	9.77	10.23	10.08	9.92	8.24	7.94	8.55	9.77	9.16	9.01	8.09	6.11
16	*C. punctiferalis* (Korea, BARCPU16)	5.50	5.80	5.80	5.65	5.65	2.75	2.60	2.44	9.77	10.23	10.08	9.92	8.24	7.94	8.55	9.77	9.16	9.01	8.09	6.11
17	*C. punctiferalis* (China, BARCPU17)	5.19	5.50	5.50	5.34	5.34	2.60	2.44	2.29	9.92	10.38	10.23	10.08	8.40	8.09	8.40	9.77	9.16	9.01	8.09	5.95
18	*C. punctiferalis* (China, BARCPU18)	5.50	5.80	5.80	5.65	5.65	2.75	2.60	2.44	9.47	9.92	9.77	9.62	7.94	7.63	8.24	9.47	9.16	9.01	8.09	6.11
19	*C. punctiferalis* (Pakistan, BARCPU19)	5.19	5.50	5.50	5.34	5.34	2.60	2.44	2.29	9.92	10.38	10.23	10.08	7.63	7.33	7.94	9.62	8.70	8.55	7.94	5.95
20	*C. punctiferalis* (Pakistan, BARCPU20)	5.50	5.80	5.80	5.65	5.65	2.75	2.60	2.44	9.77	10.23	10.08	9.92	8.24	7.94	8.55	9.77	9.16	9.01	8.09	6.11
21	*C. punctiferalis* (Pakistan, BARCPU21)	5.50	5.80	5.80	5.65	5.65	2.44	2.29	2.14	9.77	10.23	10.08	9.92	7.94	7.63	8.55	9.47	8.86	8.70	7.79	5.80
22	*C. punctiferalis* (Australia, BARCPU22)	3.82	3.82	3.82	3.97	4.28	4.43	4.58	4.43	10.08	10.53	10.38	10.23	7.33	7.02	8.55	9.62	9.31	9.47	10.08	7.02
23	*C. punctiferalis* (Australia, BARCPU23)	3.97	3.97	3.97	4.12	4.43	4.58	4.73	4.58	10.23	10.69	10.53	10.38	7.48	7.18	8.70	9.77	9.47	9.62	10.23	7.18
24	*C. punctiferalis* (Australia, BARCPU24)	3.97	3.97	3.97	4.12	4.43	4.28	4.43	4.28	10.08	10.53	10.38	10.23	7.48	7.18	8.70	9.77	9.16	9.31	9.92	7.18
25	*C. punctiferalis* (Australia, BARCPU25)	3.82	3.82	3.82	3.97	4.28	4.43	4.58	4.43	10.08	10.53	10.38	10.23	7.33	7.02	8.55	9.92	9.31	9.47	10.08	7.02
26	*C. punctiferalis* (Australia, BARCPU26)	3.97	3.97	3.97	4.12	4.43	4.58	4.73	4.58	10.23	10.69	10.53	10.38	7.48	7.18	8.70	9.77	9.47	9.62	10.23	6.87
27	*C. punctiferalis* (Australia, BARCPU27)	4.89	4.58	5.19	5.04	5.04	0.00	0.15	0.31	10.23	10.69	10.53	10.38	7.18	6.87	7.79	9.47	8.55	8.40	7.79	5.80
28	*C. punctiferalis* (Australia, BARCPU28)	3.66	3.66	3.66	3.82	4.12	4.28	4.43	4.28	9.92	10.38	10.23	10.08	7.18	6.87	8.40	9.47	9.16	9.31	9.92	6.87
29	*C. punctiferalis* (Australia, BARCPU29)	3.66	3.66	3.66	3.82	4.12	4.28	4.43	4.28	10.08	10.53	10.38	10.23	7.48	7.18	8.40	9.47	9.47	9.62	9.92	7.18
30	*C. punctiferalis* (Australia, BARCPU30)	3.97	3.97	3.97	4.12	4.43	4.58	4.73	4.58	10.23	10.69	10.53	10.38	7.48	7.18	8.70	9.77	9.31	9.47	10.08	7.18
31	*C. punctiferalis* (Australia, BARCPU31)	4.73	4.43	5.04	4.89	4.89	0.15	0.31	0.15	10.08	10.53	10.38	10.23	7.33	7.02	7.94	9.31	8.40	8.24	7.63	5.65
32	*C. punctiferalis* (Japan, BARCPU32)	5.50	5.80	5.80	5.65	5.65	2.75	2.60	2.44	9.77	10.23	10.08	9.92	8.24	7.94	8.55	9.62	9.16	9.01	8.09	6.11
33	*C. punctiferalis* (Thailand, BARCPU33)	5.04	5.34	5.34	5.19	5.19	2.60	2.44	2.29	9.31	9.77	9.62	9.47	7.94	7.63	8.24	9.31	8.86	8.70	7.79	5.95
34	*C. pinicolalis* (Current study, BARCPI01)	5.95	5.95	5.95	6.11	6.11	5.50	5.34	5.19	11.45	11.60	11.45	11.30	7.48	7.18	7.79	9.31	9.01	9.16	8.09	5.34
35	*C. pinicolalis* (Current study, BARCPI02)	6.11	6.11	6.11	6.26	6.26	5.50	5.34	5.19	11.60	11.76	11.60	11.45	7.63	7.33	7.94	9.62	9.01	9.16	8.09	5.65

36	*C. pinicolalis* (Current study, BARCPI03)	6.11	6.11	6.11	6.26	6.26	5.50	5.34	5.19	11.45	11.60	11.45	11.30	7.18	6.87	7.48	9.16	9.01	8.86	8.40	4.89
37	*C. pinicolalis* (Current study, BARCPI04)	6.26	6.26	6.26	6.41	6.41	5.80	5.65	5.50	11.45	11.60	11.45	11.30	7.18	6.87	7.48	9.31	9.01	9.16	8.40	5.34
38	*C. pinicolalis* (Current study, BARCPI05)	6.11	6.11	6.11	6.26	6.26	5.65	5.50	5.34	11.60	11.76	11.60	11.45	7.63	7.33	7.94	9.47	9.16	9.31	8.24	5.50
39	*C. pinicolalis* (Current study, BARCPI06)	5.80	5.80	5.80	5.95	5.95	5.34	5.19	5.04	11.30	11.76	11.60	11.45	7.33	7.02	7.94	9.16	8.86	9.01	7.94	5.19
40	*C. pinicolalis* (Current study, BARCPI07)	6.11	6.11	6.11	6.26	6.26	5.65	5.50	5.34	11.60	11.76	11.60	11.45	7.63	7.33	7.94	9.47	9.16	9.31	8.24	5.50
41	*C. pinicolalis* (Current study, BARCPI08)	5.95	5.95	5.95	6.11	6.11	5.50	5.34	5.19	11.30	11.45	11.30	11.15	7.33	7.02	7.94	9.47	8.86	9.01	7.94	5.65
42	*C. pinicolalis* (Current study, BARCPI09)	6.41	6.11	6.41	6.57	6.57	5.65	5.80	5.65	11.91	12.06	11.91	11.76	7.94	7.63	8.24	9.77	9.47	9.62	8.55	5.80
43	*C. pinicolalis* (Current study, BARCPI10)	5.95	5.95	5.95	6.11	6.11	5.50	5.34	5.19	11.15	11.30	11.15	10.99	7.18	6.87	7.48	9.01	9.31	9.47	8.40	5.34
44	*C. pinicolalis* (Current study, BARCPI11)	6.41	6.41	6.41	6.57	6.57	5.65	5.50	5.34	11.60	11.76	11.60	11.45	7.18	6.87	7.48	9.16	9.01	9.16	8.40	5.50
45	*C. pinicolalis* (Current study, BARCPI12)	6.11	6.11	6.11	6.26	6.26	5.65	5.50	5.34	11.60	11.76	11.60	11.45	7.63	7.33	7.63	9.47	9.16	9.31	8.24	5.50
46	*C. pluto* (Papua New Guinea, Autralia, BARCPL01)	6.72	6.41	6.72	6.57	6.57	6.26	6.41	6.26	10.84	11.30	11.15	10.99	7.33	7.02	8.40	10.08	9.31	9.47	9.77	5.95
47	*C. pluto* (Australia, BARCPL02)	6.87	6.57	6.87	6.72	6.72	6.41	6.57	6.41	10.99	11.45	11.30	11.15	7.48	7.18	8.55	10.23	9.47	9.62	9.92	6.11
48	*C. pluto* (Australia, BARCPL03)	6.57	6.26	6.57	6.41	6.41	6.11	6.26	6.11	10.69	11.15	10.99	10.84	7.48	7.18	8.24	9.92	9.16	9.31	9.62	5.80
49	*C. pluto* (Australia, BARCPL04)	6.57	6.26	6.57	6.41	6.41	6.26	6.41	6.26	10.84	11.30	11.15	10.99	7.63	7.33	8.70	9.77	9.62	9.77	9.77	5.95
50	*C. pluto* (Australia, BARCPL05)	6.57	6.26	6.57	6.41	6.41	6.11	6.26	6.11	10.69	11.15	10.99	10.84	7.18	6.87	8.24	10.08	9.31	9.47	9.77	6.11
51	*C. semifascialis* (Papua New Guinea, BARCSE01)	-	0.31	0.31	0.15	0.46	4.89	4.73	4.58	10.53	10.99	10.84	10.69	8.70	8.40	9.16	9.01	9.62	9.77	9.47	7.02
52	*C. semifascialis* (Papua New Guinea, BARCSE02)	2	-	0.61	0.46	0.76	4.58	4.73	4.58	10.53	10.99	10.84	10.69	8.70	8.40	9.16	9.01	9.62	9.77	9.47	7.02
53	*C. semifascialis* (Papua New Guinea, BARCSE03)	2	4	-	0.46	0.76	5.19	5.04	4.89	10.53	10.99	10.84	10.69	8.70	8.40	9.16	9.16	9.77	9.92	9.62	7.02
54	*C. semifascialis* (Australia, BARCSE04)	1	3	3	-	0.31	5.04	4.89	4.73	10.69	11.15	10.99	10.84	8.86	8.55	9.31	9.16	9.77	9.92	9.62	6.87
55	*C. semifascialis* (Australia, BARCSE05)	3	5	5	2	-	5.04	4.89	4.73	10.99	11.45	11.30	11.15	8.86	8.55	9.31	9.16	9.77	9.92	9.62	6.87
56	*C. semifascialis* (Australia, BARCSE06)	32	30	34	33	33	-	0.15	0.31	10.23	10.69	10.53	10.38	7.18	6.87	7.79	9.47	8.55	8.40	7.79	5.80
57	*C. semifascialis* (Australia, BARCSE07)	31	31	33	32	32	1	-	0.15	10.08	10.53	10.38	10.23	7.02	6.72	7.63	9.31	8.40	8.24	7.63	5.65
58	*C. semifascialis* (Australia, BARCSE08)	30	30	32	31	31	2	1	-	9.92	10.38	10.23	10.08	7.18	6.87	7.79	9.16	8.24	8.09	7.48	5.50
59	*C. tharsalea* (Australia, BARCTH01)	69	69	69	70	72	67	66	65	-	0.61	0.61	0.15	11.30	11.15	12.06	10.99	11.60	11.76	11.60	11.60
60	*C. tharsalea* (Australia, BARCTH02)	72	72	72	73	75	70	69	68	4	-	0.31	0.46	11.60	11.45	12.06	11.60	11.76	11.91	11.91	12.06
61	*C. tharsalea* (Australia, BARCTH03)	71	71	71	72	74	69	68	67	4	2	-	0.46	11.60	11.15	11.76	11.60	11.91	12.06	11.91	11.91
62	*C. tharsalea* (Australia, BARCTH04)	70	70	70	71	73	68	67	66	1	3	3	-	11.45	11.30	11.91	11.15	11.76	11.91	11.76	11.76
63	*C. ersealis* (Australia, BARCER01)	57	57	57	58	58	47	46	47	74	76	76	75	-	0.46	3.05	8.86	9.31	9.31	9.47	8.24
64	*C. ersealis* (Australia, BARCER02)	55	55	55	56	56	45	44	45	73	75	73	74	3	-	2.60	8.70	9.16	9.16	9.31	7.94
65	*C. diminutiva* (Australia, BARCDI01)	60	60	60	61	61	51	50	51	79	79	77	78	20	17	-	9.62	10.69	10.69	10.23	8.40
66	*C. haemactalis* (Australia, BARCHA01)	59	59	60	60	60	62	61	60	72	76	76	73	58	57	63	-	9.47	9.62	9.31	10.23
67	*C. evaxalis* (Australia, BARCEV01)	63	63	64	64	64	56	55	54	76	77	78	77	61	60	70	62	-	0.15	1.83	9.62
68	*C. evaxalis* (Australia, BARCEV02)	64	64	65	65	65	55	54	53	77	78	79	78	61	60	70	63	1	-	1.68	9.47
69	*C. evaxalis* (Australia, BARCEV03)	62	62	63	63	63	51	50	49	76	78	78	77	62	61	67	61	12	11	-	9.47
70	*C. pandamalis* (Malaysia, BARCPA01)	46	46	46	45	45	38	37	36	76	79	78	77	54	52	55	67	63	62	62	-

Table S5. Pairwise comparisons of *EF1α* haplotypes of *Conogethes pinicolalis* and *C. punctiferalis*

		1	2	3	4	5	6	7	8	9	10	11	12	13	14	15	16	17	18	19	20	21	22	23	24	25	26	27	28	29	30
1	EF1ACPU01	-	0.13	0.25	0.13	0.13	0.76	0.25	0.25	0.38	0.13	0.64	0.76	0.13	0.25	0.25	0.13	0.13	0.13	0.25	0.51	0.64	0.13	0.13	0.38	0.13	0.25	0.25	0.25	0.25	0.13
2	EF1ACPU02	1	-	0.38	0.25	0.25	0.89	0.38	0.38	0.51	0.25	0.76	0.89	0.25	0.38	0.38	0.25	0.25	0.25	0.38	0.64	0.76	0.25	0.25	0.51	0.25	0.38	0.38	0.38	0.38	0.25
3	EF1ACPU03	2	3	-	0.38	0.38	1.02	0.51	0.51	0.64	0.38	0.89	1.02	0.38	0.51	0.51	0.38	0.38	0.38	0.51	0.76	0.89	0.38	0.38	0.38	0.38	0.51	0.51	0.51	0.51	0.38
4	EF1ACPU04	1	2	3	-	0.25	0.89	0.38	0.38	0.51	0.25	0.76	0.89	0.25	0.38	0.38	0.25	0.25	0.25	0.38	0.64	0.76	0.25	0.25	0.51	0.25	0.38	0.38	0.38	0.38	0.25
5	EF1ACPU05	1	2	3	2	-	0.89	0.38	0.38	0.51	0.25	0.76	0.89	0.25	0.38	0.38	0.25	0.25	0.25	0.38	0.64	0.76	0.25	0.25	0.51	0.25	0.38	0.38	0.38	0.38	0.25
6	EF1ACPU06	6	7	8	7	7	-	0.76	0.76	0.89	0.89	0.89	1.27	0.89	0.76	0.76	0.89	0.89	0.89	0.76	1.27	1.40	0.89	0.89	1.14	0.89	1.02	1.02	1.02	1.02	0.89
7	EF1ACPU07	2	3	4	3	3	6	-	0.25	0.38	0.38	0.89	1.02	0.38	0.51	0.51	0.38	0.38	0.38	0.51	0.76	0.89	0.38	0.38	0.64	0.38	0.51	0.51	0.51	0.51	0.38
8	EF1ACPU08	2	3	4	3	3	6	2	-	0.38	0.38	0.89	1.02	0.38	0.51	0.51	0.38	0.38	0.38	0.51	0.76	0.89	0.38	0.38	0.64	0.38	0.51	0.51	0.51	0.51	0.38
9	EF1ACPU09	3	4	5	4	4	7	3	3	-	0.51	1.02	1.14	0.51	0.64	0.64	0.51	0.51	0.51	0.64	0.89	1.02	0.51	0.51	0.76	0.51	0.64	0.64	0.64	0.64	0.51
10	EF1ACPU10	1	2	3	2	2	7	3	3	4	-	0.76	0.64	0.25	0.38	0.38	0.25	0.25	0.25	0.38	0.64	0.76	0.25	0.25	0.51	0.25	0.38	0.38	0.38	0.38	0.25
11	EF1ACPU11	5	6	7	6	6	7	7	7	8	6	-	1.14	0.76	0.64	0.64	0.76	0.76	0.76	0.64	1.14	0.76	0.76	0.76	1.02	0.76	0.89	0.89	0.89	0.89	0.76
12	EF1ACPU12	6	7	8	7	7	10	8	8	9	5	9	-	0.89	0.76	0.76	0.89	0.89	0.89	0.76	1.27	1.40	0.89	0.89	1.14	0.89	1.02	1.02	1.02	1.02	0.89
13	EF1ACPU13	1	2	3	2	2	7	3	3	4	2	6	7	-	0.38	0.38	0.25	0.25	0.25	0.38	0.64	0.76	0.25	0.25	0.51	0.25	0.38	0.38	0.38	0.38	0.25
14	EF1ACPU14	2	3	4	3	3	6	4	4	5	3	5	6	3	-	0.25	0.38	0.38	0.38	0.25	0.76	0.89	0.38	0.38	0.64	0.38	0.51	0.51	0.51	0.51	0.38
15	EF1ACPU15	2	3	4	3	3	6	4	4	5	3	5	6	3	2	-	0.38	0.38	0.38	0.25	0.76	0.89	0.38	0.38	0.64	0.38	0.51	0.51	0.51	0.51	0.38
16	EF1ACPU16	1	2	3	2	2	7	3	3	4	2	6	7	2	3	3	-	0.25	0.25	0.38	0.64	0.76	0.25	0.25	0.51	0.25	0.38	0.38	0.38	0.38	0.25
17	EF1ACPU17	1	2	3	2	2	7	3	3	4	2	6	7	2	3	3	2	-	0.25	0.38	0.64	0.76	0.25	0.25	0.51	0.25	0.38	0.38	0.38	0.38	0.25
18	EF1ACPU18	1	2	3	2	2	7	3	3	4	2	6	7	2	3	3	2	2	-	0.38	0.64	0.76	0.25	0.25	0.51	0.25	0.38	0.38	0.38	0.38	0.25
19	EF1ACPU19	2	3	4	3	3	6	4	4	5	3	5	6	3	2	2	3	3	3	-	0.76	0.89	0.38	0.38	0.64	0.38	0.51	0.51	0.51	0.51	0.38
20	EF1ACPU20	4	5	6	5	5	10	6	6	7	5	9	10	5	6	6	5	5	5	6	-	1.14	0.64	0.64	0.89	0.64	0.76	0.76	0.76	0.76	0.64
21	EF1ACPU21	5	6	7	6	6	11	7	7	8	6	6	11	6	7	7	6	6	6	7	9	-	0.76	0.76	1.02	0.76	0.89	0.89	0.89	0.89	0.76
22	EF1ACPU22	1	2	3	2	2	7	3	3	4	2	6	7	2	3	3	2	2	2	3	5	6	-	0.25	0.51	0.25	0.38	0.38	0.38	0.38	0.25
23	EF1ACPU23	1	2	3	2	2	7	3	3	4	2	6	7	2	3	3	2	2	2	3	5	6	2	-	0.51	0.25	0.38	0.38	0.38	0.38	0.25
24	EF1ACPU24	3	4	3	4	4	9	5	5	6	4	8	9	4	5	5	4	4	4	5	7	8	4	4	-	0.51	0.64	0.64	0.64	0.64	0.51
25	EF1ACPU25	1	2	3	2	2	7	3	3	4	2	6	7	2	3	3	2	2	2	3	5	6	2	2	4	-	0.13	0.38	0.38	0.38	0.25
26	EF1ACPU26	2	3	4	3	3	8	4	4	5	3	7	8	3	4	4	3	3	3	4	6	7	3	3	5	1	-	0.51	0.51	0.51	0.38
27	EF1ACPU27	2	3	4	3	3	8	4	4	5	3	7	8	3	4	4	3	3	3	4	6	7	3	3	5	3	4	-	0.51	0.51	0.38
28	EF1ACPU28	2	3	4	3	3	8	4	4	5	3	7	8	3	4	4	3	3	3	4	6	7	3	3	5	3	4	4	-	0.51	0.38
29	EF1ACPU29	2	3	4	3	3	8	4	4	5	3	7	8	3	4	4	3	3	3	4	6	7	3	3	5	3	4	4	4	-	0.38
30	EF1ACPU30	1	2	3	2	2	7	3	3	4	2	6	7	2	3	3	2	2	2	3	5	6	2	2	4	2	3	3	3	3	-
31	EF1ACPU31	2	3	4	3	3	8	4	4	5	3	7	8	3	4	4	3	3	3	4	6	7	3	3	5	3	4	4	4	4	1
32	EF1ACPU32	1	2	3	2	2	7	3	3	4	2	6	7	2	3	3	2	2	2	3	5	6	2	2	4	2	3	3	3	3	2
33	EF1ACPU33	3	4	5	4	4	9	5	5	6	4	8	9	4	5	5	4	4	4	5	7	8	4	4	6	4	5	5	5	5	4
34	EF1ACPU34	2	3	4	3	3	8	4	4	5	3	5	8	3	4	4	3	3	3	4	6	5	3	3	5	3	4	4	4	4	3
35	EF1ACPU35	3	4	5	4	4	9	5	5	6	4	8	9	4	5	5	4	4	4	5	6	8	4	4	6	4	5	5	5	5	4
36	EF1ACPU36	5	6	7	6	6	11	7	7	8	6	10	11	6	7	7	6	6	6	7	9	10	6	6	8	6	7	7	7	7	6
37	EF1ACPU37	1	2	3	2	2	7	3	3	4	2	6	7	2	3	3	2	2	2	3	5	6	2	2	4	2	3	3	3	3	2
38	EF1ACPU38	2	3	4	3	3	8	4	4	5	3	7	8	3	4	4	3	3	3	4	6	7	3	3	5	3	4	4	4	4	3
39	EF1ACPU39	6	7	8	7	7	10	8	8	9	7	9	10	7	6	6	7	7	7	6	10	11	7	7	9	7	8	8	8	8	7
40	EF1ACPU40	7	8	9	8	8	11	9	9	10	8	10	11	8	7	7	8	8	8	7	11	12	8	8	10	8	9	9	9	9	8
41	EF1ACPU41	6	7	8	7	7	10	8	8	9	7	9	10	7	6	6	7	7	7	6	10	11	7	7	9	7	8	8	8	8	7
42	EF1ACPU42	3	4	5	4	4	9	5	5	6	4	8	9	4	5	5	4	4	4	5	7	8	4	4	6	4	5	5	5	5	4
43	EF1ACPU43	2	3	4	3	3	6	4	4	5	3	5	6	3	2	2	3	3	3	2	6	7	3	3	5	3	4	4	4	4	3
44	EF1ACPU44	5	6	7	6	6	9	7	7	8	6	8	9	6	5	5	6	6	6	5	9	10	6	6	8	6	7	7	7	7	6
45	EF1ACPU45	4	5	6	5	5	7	6	6	7	5	7	8	5	4	4	5	5	5	4	8	9	5	5	7	5	6	6	6	6	5
46	EF1ACPU46	3	4	5	4	4	7	5	5	6	4	6	7	4	3	3	4	4	4	3	7	8	4	4	6	4	5	5	5	5	4
47	EF1ACPU47	4	5	6	5	5	6	6	6	7	5	1	8	5	4	4	5	5	5	4	8	5	5	5	7	5	6	6	6	6	5
48	EF1ACPU48	4	5	6	5	5	10	6	6	7	3	9	6	5	6	6	5	5	5	6	8	9	5	5	7	5	6	6	6	6	5
49	EF1ACPU49	2	3	4	3	3	6	4	4	5	3	5	4	3	2	2	3	3	3	2	6	7	3	3	5	3	4	4	4	4	3
50	EF1ACPU50	2	3	4	3	3	8	4	4	5	1	7	6	3	4	4	3	3	3	4	6	7	3	3	5	3	4	4	4	4	3
51	EF1ACPU51	4	5	6	5	5	8	6	6	7	5	7	4	5	4	4	5	5	5	4	8	9	5	5	7	5	6	6	6	6	5
52	EF1ACPU52	3	4	5	4	4	7	5	5	6	4	6	3	4	3	3	4	4	4	3	7	8	4	4	6	4	5	5	5	5	4
53	EF1ACPU53	3	4	5	4	4	9	5	5	6	2	8	7	4	5	5	4	4	4	5	7	8	4	4	6	4	5	5	5	5	4
54	EF1ACPU54	2	3	4	3	3	8	4	4	5	3	7	8	3	4	4	3	3	3	4	6	7	3	3	5	3	4	4	4	4	3
55	EF1ACPU55	1	2	3	2	2	7	3	3	4	2	6	7	2	3	3	2	2	2	3	5	6	2	2	4	2	3	3	3	3	2
56	EF1ACPI01	11	12	13	12	12	15	13	13	14	12	14	15	12	11	11	12	12	12	11	15	16	12	12	14	12	13	13	13	13	12
57	EF1ACPI02	12	13	14	13	13	16	14	14	15	13	15	16	13	12	12	13	13	13	12	16	17	13	13	15	13	14	14	14	14	13
58	EF1ACPI03	12	13	14	13	13	16	14	14	15	13	15	16	13	12	12	13	13	13	12	16	17	13	13	15	13	14	14	14	14	13

59	EF1ACPI04	15	16	17	16	16	19	17	17	18	16	18	19	16	15	15	16	16	16	15	19	20	16	16	18	16	17	17	17	17	16
60	EF1ACPI05	13	14	15	14	14	17	15	15	16	14	16	17	14	13	13	14	14	14	13	17	18	14	14	16	14	15	15	15	15	14
61	EF1ACPI06	15	16	17	16	16	19	17	17	18	16	18	19	16	15	15	16	16	16	15	19	20	16	16	18	16	17	17	17	17	16
62	EF1ACPI07	13	14	15	14	14	17	15	15	16	14	16	17	14	13	13	14	14	14	13	17	18	14	14	16	14	15	15	15	15	14
63	EF1ACPI08	14	15	16	15	15	18	16	16	17	15	17	18	15	14	14	15	15	15	14	18	19	15	15	17	15	16	16	16	16	15
64	EF1ACPI09	13	14	15	14	14	17	15	15	16	14	16	17	14	13	13	14	14	14	13	17	18	14	14	16	14	15	15	15	15	14
65	EF1ACPI10	15	16	17	16	16	19	17	17	18	16	18	19	16	15	15	16	16	16	15	19	20	16	16	18	16	17	17	17	17	16
66	EF1ACPI11	8	9	10	9	9	12	10	10	11	9	11	12	9	8	8	9	9	9	8	12	13	9	9	11	9	10	10	10	10	9
67	EF1ACPI12	12	13	14	13	13	16	14	14	15	13	15	16	13	12	12	13	13	13	12	16	17	13	13	15	13	14	14	14	14	13
68	EF1ACPI13	13	14	15	14	14	17	15	15	16	14	16	17	14	13	13	14	14	14	13	17	18	14	14	16	14	15	15	15	15	14
69	EF1ACPI14	13	14	15	14	14	17	15	15	16	14	16	17	14	13	13	14	14	14	13	17	18	14	14	16	14	15	15	15	15	14
70	EF1ACPI15	14	15	16	15	15	18	16	16	17	15	17	18	15	14	14	15	15	15	14	18	19	15	15	17	15	16	16	16	16	15
71	EF1ACPI16	12	13	14	13	13	16	14	14	15	13	15	16	13	12	12	13	13	13	12	16	17	13	13	15	13	14	14	14	14	13
72	EF1ACPI17	18	19	20	19	19	22	20	20	21	19	21	22	19	18	18	19	19	19	18	22	23	19	19	21	19	20	20	20	20	19
73	EF1ACPI18	42	43	44	43	43	45	43	43	44	43	42	44	41	42	42	43	43	43	40	46	44	43	43	45	41	40	44	44	44	43
74	EF1ACPI19	13	14	15	14	14	17	15	15	16	14	16	17	14	13	13	14	14	14	13	17	18	14	14	16	14	15	15	15	15	14
75	EF1ACPI20	16	17	18	17	17	20	18	18	19	17	19	20	17	16	16	17	17	17	16	20	21	17	17	19	17	18	18	18	18	17
76	EF1ACPI21	13	14	15	14	14	17	15	15	16	14	16	17	14	13	13	14	14	14	13	17	18	14	14	16	14	15	15	15	15	14
77	EF1ACPI22	17	18	19	18	18	21	19	19	20	18	20	21	18	17	17	18	18	18	17	21	22	18	18	20	18	19	19	19	19	18
78	EF1ACPI23	15	16	17	16	16	19	17	17	18	16	18	19	16	15	15	16	16	16	15	19	20	16	16	18	16	17	17	17	17	16
79	EF1ACPI24	13	14	15	14	14	17	15	15	16	14	16	17	14	13	13	14	14	14	13	17	18	14	14	16	14	15	15	15	15	14
80	EF1ACPI25	13	14	15	14	14	17	15	15	16	14	16	17	14	13	13	14	14	14	13	17	18	14	14	16	14	15	15	15	15	14
81	EF1ACPI26	18	19	20	19	19	22	20	20	21	19	21	22	19	18	18	19	19	19	18	22	23	19	19	20	19	20	20	20	20	19
82	EF1ACPI27	16	17	18	17	17	20	18	18	19	17	19	20	17	16	16	17	17	17	16	20	21	17	17	19	17	18	18	18	18	17
83	EF1ACPI28	13	14	15	14	14	17	15	15	16	14	16	17	14	13	13	14	14	14	13	17	18	14	14	16	14	15	15	15	15	14
84	EF1ACPI29	13	14	15	14	14	17	15	15	16	14	16	17	14	13	13	14	14	14	13	17	18	14	14	16	14	15	15	15	15	14
85	EF1ACPI30	15	16	17	16	16	19	17	17	18	16	18	19	16	15	15	16	16	16	15	19	20	16	16	18	16	17	17	17	17	16
86	EF1ACPI31	13	14	15	14	14	17	15	15	16	14	16	17	14	13	13	14	14	14	13	17	18	14	14	16	14	15	15	15	15	14
87	EF1ACPI32	14	15	16	15	15	18	16	16	17	15	17	18	15	14	14	15	15	15	14	18	19	15	15	17	15	16	14	16	16	15
88	EF1ACPI33	13	14	15	14	14	17	15	15	16	14	16	17	14	13	13	14	14	14	13	17	18	14	14	16	14	15	15	15	15	14
89	EF1ACPI34	14	15	16	15	15	18	16	16	17	15	17	18	15	14	14	15	15	15	14	18	19	15	15	17	15	16	16	16	16	15
90	EF1ACPI35	14	15	16	15	15	18	16	16	17	15	17	18	15	14	14	15	15	15	14	18	19	15	15	17	15	16	16	16	16	15
91	EF1ACPI36	13	14	15	14	14	17	15	15	16	14	16	17	14	13	13	14	14	14	13	17	18	14	14	16	14	15	15	15	15	14
92	EF1ACPI37	15	16	17	16	16	19	17	17	18	16	18	19	16	15	15	16	16	16	15	19	20	16	16	18	16	17	17	17	17	16
93	EF1ACPI38	15	16	17	16	16	19	17	17	18	16	18	19	16	14	15	16	16	16	15	19	20	16	16	18	16	17	17	17	17	16
94	EF1ACPI39	14	15	16	15	15	18	16	16	17	15	17	16	15	14	14	15	15	15	14	18	19	15	15	17	15	16	16	16	16	15
95	EF1ACPI40	18	19	20	19	19	21	20	20	21	19	21	22	19	17	18	19	19	19	18	22	23	19	19	21	19	20	20	20	20	19
96	EF1ACPI41	14	15	16	15	15	18	16	16	17	15	17	18	15	13	14	15	15	15	14	18	19	15	15	17	15	16	16	16	16	15
97	EF1ACPI42	17	18	19	18	18	21	19	19	20	18	20	21	18	16	17	18	18	18	17	21	20	18	18	20	18	19	19	19	19	18
98	PI1	15	16	17	16	16	19	17	17	18	16	18	19	16	15	14	16	16	16	15	19	20	16	16	18	16	17	17	17	17	16
99	PI2	17	18	19	18	18	21	19	19	20	18	20	21	18	17	17	18	18	18	17	21	22	18	18	20	18	19	19	19	19	18
100	PI3	14	15	16	15	15	18	16	16	17	15	17	18	15	14	14	15	15	15	14	18	19	15	15	17	15	16	16	16	16	15
101	PI4	16	17	18	17	17	20	18	18	19	17	19	20	17	16	16	17	17	17	16	20	21	17	17	19	17	18	18	18	18	17
102	PI5	13	14	15	14	14	17	15	15	16	14	16	17	14	13	13	14	14	14	13	17	18	14	14	16	14	15	15	15	15	14
103	PI6	16	17	18	17	17	20	18	18	19	17	19	20	17	16	16	17	17	17	16	20	21	17	17	19	17	18	18	18	18	17
104	PU1	5	6	7	6	6	5	5	5	6	6	6	9	6	5	5	6	6	6	5	9	10	6	6	8	6	7	7	7	7	6
105	PU2	4	5	6	5	5	10	6	6	7	5	9	10	5	6	6	5	5	5	6	8	9	5	5	7	5	6	6	6	6	5
106	PU3	2	3	4	3	3	8	4	4	5	3	7	8	3	4	4	3	3	3	4	6	7	3	3	5	3	4	4	4	4	3
107	PU4	4	5	6	5	5	10	6	6	7	5	9	10	5	6	6	5	5	5	6	8	9	5	5	7	5	6	6	6	6	5
108	PU5	2	3	4	3	3	8	4	4	5	3	7	8	3	4	4	3	3	3	4	6	7	3	3	5	3	4	4	4	4	3
109	PU6	4	5	6	5	5	8	6	6	7	5	7	8	5	4	4	5	5	5	4	8	9	5	5	7	5	6	6	6	6	5
110	PU7	5	6	7	6	6	9	7	7	8	6	8	9	6	5	4	6	6	6	5	9	10	6	6	8	6	7	7	7	7	6

		31	32	33	34	35	36	37	38	39	40	41	42	43	44	45	46	47	48	49	50	51	52	53	54	55	56	57	58	59	60
1	EF1ACPU01	0.25	0.13	0.38	0.25	0.38	0.64	0.13	0.25	0.76	0.89	0.76	0.38	0.25	0.64	0.51	0.38	0.51	0.51	0.25	0.25	0.51	0.38	0.38	0.25	0.13	1.40	1.52	1.52	1.90	1.65
2	EF1ACPU02	0.38	0.25	0.51	0.38	0.51	0.76	0.25	0.38	0.89	1.02	0.89	0.51	0.38	0.76	0.64	0.51	0.64	0.64	0.38	0.38	0.64	0.51	0.51	0.38	0.25	1.52	1.65	1.65	2.03	1.78
3	EF1ACPU03	0.51	0.38	0.64	0.51	0.64	0.89	0.38	0.51	1.02	1.14	1.02	0.64	0.51	0.89	0.76	0.64	0.76	0.76	0.51	0.51	0.76	0.64	0.64	0.51	0.38	1.65	1.78	1.78	2.16	1.90
4	EF1ACPU04	0.38	0.25	0.51	0.38	0.51	0.76	0.25	0.38	0.89	1.02	0.89	0.51	0.38	0.76	0.64	0.51	0.64	0.64	0.38	0.38	0.64	0.51	0.51	0.38	0.25	1.52	1.65	1.65	2.03	1.78
5	EF1ACPU05	0.38	0.25	0.51	0.38	0.51	0.76	0.25	0.38	0.89	1.02	0.89	0.51	0.38	0.76	0.64	0.51	0.64	0.64	0.38	0.38	0.64	0.51	0.51	0.38	0.25	1.52	1.65	1.65	2.03	1.78
6	EF1ACPU06	1.02	0.89	1.14	1.02	1.14	1.40	0.89	1.02	1.27	1.40	1.27	1.14	0.76	1.14	0.89	0.89	0.76	1.27	0.76	1.02	1.02	0.89	1.14	1.02	0.89	1.90	2.03	2.03	2.41	2.16
7	EF1ACPU07	0.51	0.38	0.64	0.51	0.64	0.89	0.38	0.51	1.02	1.14	1.02	0.64	0.51	0.89	0.76	0.64	0.76	0.76	0.51	0.51	0.76	0.64	0.64	0.51	0.38	1.65	1.78	1.78	2.16	1.90
8	EF1ACPU08	0.51	0.38	0.64	0.51	0.64	0.89	0.38	0.51	1.02	1.14	1.02	0.64	0.51	0.89	0.76	0.64	0.76	0.76	0.51	0.51	0.76	0.64	0.64	0.51	0.38	1.65	1.78	1.78	2.16	1.90
9	EF1ACPU09	0.64	0.51	0.76	0.64	0.76	1.02	0.51	0.64	1.14	1.27	1.14	0.76	0.64	1.02	0.89	0.76	0.89	0.89	0.64	0.64	0.89	0.76	0.76	0.64	0.51	1.78	1.90	1.90	2.28	2.03
10	EF1ACPU10	0.38	0.25	0.51	0.38	0.51	0.76	0.25	0.38	0.89	1.02	0.89	0.51	0.38	0.76	0.64	0.51	0.64	0.38	0.38	0.13	0.64	0.51	0.25	0.38	0.25	1.52	1.65	1.65	2.03	1.78
11	EF1ACPU11	0.89	0.76	1.02	0.64	1.02	1.27	0.76	0.89	1.14	1.27	1.14	1.02	0.64	1.02	0.89	0.76	0.13	1.14	0.64	0.89	0.89	0.76	1.02	0.89	0.76	1.78	1.90	1.90	2.28	2.03
12	EF1ACPU12	1.02	0.89	1.14	1.02	1.14	1.40	0.89	1.02	1.27	1.40	1.27	1.14	0.76	1.14	1.02	0.89	1.02	0.76	0.51	0.76	0.51	0.38	0.89	1.02	0.89	1.90	2.03	2.03	2.41	2.16
13	EF1ACPU13	0.38	0.25	0.51	0.38	0.51	0.76	0.25	0.38	0.89	1.02	0.89	0.51	0.38	0.76	0.64	0.51	0.64	0.64	0.38	0.38	0.64	0.51	0.51	0.38	0.25	1.52	1.65	1.65	2.03	1.78
14	EF1ACPU14	0.51	0.38	0.64	0.51	0.64	0.89	0.38	0.51	0.76	0.89	0.76	0.64	0.25	0.64	0.51	0.38	0.51	0.76	0.25	0.51	0.51	0.38	0.64	0.51	0.38	1.40	1.52	1.52	1.90	1.65
15	EF1ACPU15	0.51	0.38	0.64	0.51	0.64	0.89	0.38	0.51	0.76	0.89	0.76	0.64	0.25	0.64	0.51	0.38	0.51	0.76	0.25	0.51	0.51	0.38	0.64	0.51	0.38	1.40	1.52	1.52	1.90	1.65
16	EF1ACPU16	0.38	0.25	0.51	0.38	0.51	0.76	0.25	0.38	0.89	1.02	0.89	0.51	0.38	0.76	0.64	0.51	0.64	0.64	0.38	0.38	0.64	0.51	0.51	0.38	0.25	1.52	1.65	1.65	2.03	1.78
17	EF1ACPU17	0.38	0.25	0.51	0.38	0.51	0.76	0.25	0.38	0.89	1.02	0.89	0.51	0.38	0.76	0.64	0.51	0.64	0.64	0.38	0.38	0.64	0.51	0.51	0.38	0.25	1.52	1.65	1.65	2.03	1.78
18	EF1ACPU18	0.38	0.25	0.51	0.38	0.51	0.76	0.25	0.38	0.89	1.02	0.89	0.51	0.38	0.76	0.64	0.51	0.64	0.64	0.38	0.38	0.64	0.51	0.51	0.38	0.25	1.52	1.65	1.65	2.03	1.78
19	EF1ACPU19	0.51	0.38	0.64	0.51	0.64	0.89	0.38	0.51	0.76	0.89	0.76	0.64	0.25	0.64	0.51	0.38	0.51	0.76	0.25	0.51	0.51	0.38	0.64	0.51	0.38	1.40	1.52	1.52	1.90	1.65
20	EF1ACPU20	0.76	0.64	0.89	0.76	0.76	1.14	0.64	0.76	1.27	1.40	1.27	0.89	0.76	1.14	1.02	0.89	1.02	1.02	0.76	0.76	1.02	0.89	0.89	0.76	0.64	1.90	2.03	2.03	2.41	2.16
21	EF1ACPU21	0.89	0.76	1.02	0.64	1.02	1.27	0.76	0.89	1.40	1.52	1.40	1.02	0.89	1.27	1.14	1.02	0.64	1.14	0.89	0.89	1.14	1.02	1.02	0.89	0.76	2.03	2.16	2.16	2.54	2.28
22	EF1ACPU22	0.38	0.25	0.51	0.38	0.51	0.76	0.25	0.38	0.89	1.02	0.89	0.51	0.38	0.76	0.64	0.51	0.64	0.64	0.38	0.38	0.64	0.51	0.51	0.38	0.25	1.52	1.65	1.65	2.03	1.78
23	EF1ACPU23	0.38	0.25	0.51	0.38	0.51	0.76	0.25	0.38	0.89	1.02	0.89	0.51	0.38	0.76	0.64	0.51	0.64	0.64	0.38	0.38	0.64	0.51	0.51	0.38	0.25	1.52	1.65	1.65	2.03	1.78
24	EF1ACPU24	0.64	0.51	0.76	0.64	0.76	1.02	0.51	0.64	1.14	1.27	1.14	0.76	0.64	1.02	0.89	0.76	0.89	0.89	0.64	0.64	0.89	0.76	0.76	0.64	0.51	1.78	1.90	1.90	2.28	2.03
25	EF1ACPU25	0.38	0.25	0.51	0.38	0.51	0.76	0.25	0.38	0.89	1.02	0.89	0.51	0.38	0.76	0.64	0.51	0.64	0.64	0.38	0.38	0.64	0.51	0.51	0.38	0.25	1.52	1.65	1.65	2.03	1.78
26	EF1ACPU26	0.51	0.38	0.64	0.51	0.64	0.89	0.38	0.51	1.02	1.14	1.02	0.64	0.51	0.89	0.76	0.64	0.76	0.76	0.51	0.51	0.76	0.64	0.64	0.51	0.38	1.65	1.78	1.78	2.16	1.90
27	EF1ACPU27	0.51	0.38	0.64	0.51	0.64	0.89	0.38	0.51	1.02	1.14	1.02	0.64	0.51	0.89	0.76	0.64	0.76	0.76	0.51	0.51	0.76	0.64	0.64	0.51	0.38	1.65	1.78	1.78	2.16	1.90
28	EF1ACPU28	0.51	0.38	0.64	0.51	0.64	0.89	0.38	0.51	1.02	1.14	1.02	0.64	0.51	0.89	0.76	0.64	0.76	0.76	0.51	0.51	0.76	0.64	0.64	0.51	0.38	1.65	1.78	1.78	2.16	1.90
29	EF1ACPU29	0.51	0.38	0.64	0.51	0.64	0.89	0.38	0.51	1.02	1.14	1.02	0.64	0.51	0.89	0.76	0.64	0.76	0.76	0.51	0.51	0.76	0.64	0.64	0.51	0.38	1.65	1.78	1.78	2.16	1.90
30	EF1ACPU30	0.13	0.25	0.51	0.38	0.51	0.76	0.25	0.38	0.89	1.02	0.89	0.51	0.38	0.76	0.64	0.51	0.64	0.64	0.38	0.38	0.64	0.51	0.51	0.38	0.25	1.52	1.65	1.65	2.03	1.78
31	EF1ACPU31	-	0.38	0.64	0.51	0.64	0.89	0.38	0.51	1.02	1.14	1.02	0.64	0.51	0.89	0.76	0.64	0.76	0.76	0.51	0.51	0.76	0.64	0.64	0.51	0.38	1.65	1.78	1.78	2.16	1.90
32	EF1ACPU32	3	-	0.51	0.38	0.51	0.76	0.25	0.38	0.89	1.02	0.89	0.51	0.38	0.76	0.64	0.51	0.64	0.64	0.38	0.38	0.64	0.51	0.51	0.38	0.25	1.52	1.65	1.65	2.03	1.78
33	EF1ACPU33	5	4	-	0.64	0.76	1.02	0.51	0.64	1.14	1.27	1.14	0.76	0.64	1.02	0.89	0.76	0.89	0.89	0.64	0.38	0.89	0.76	0.76	0.64	0.51	1.52	1.65	1.65	2.03	1.78
34	EF1ACPU34	4	3	5	-	0.64	0.89	0.38	0.51	1.02	1.14	1.02	0.64	0.51	0.89	0.76	0.64	0.51	0.76	0.51	0.51	0.76	0.64	0.64	0.51	0.38	1.65	1.78	1.78	2.16	1.90
35	EF1ACPU35	5	4	6	5	-	1.02	0.51	0.64	1.14	1.27	1.14	0.76	0.64	1.02	0.89	0.76	0.89	0.89	0.64	0.64	0.89	0.76	0.76	0.64	0.51	1.78	1.90	1.90	2.28	2.03
36	EF1ACPU36	7	6	8	7	8	-	0.76	0.89	1.40	1.52	1.40	1.02	0.89	1.27	1.14	1.02	1.14	1.14	0.89	0.89	1.14	1.02	1.02	0.89	0.76	2.03	2.16	2.16	2.54	2.28
37	EF1ACPU37	3	2	4	3	4	6	-	0.38	0.89	1.02	0.89	0.51	0.38	0.76	0.64	0.51	0.64	0.64	0.38	0.38	0.64	0.51	0.51	0.38	0.25	1.52	1.65	1.65	2.03	1.78
38	EF1ACPU38	4	3	5	4	5	7	3	-	1.02	1.14	1.02	0.64	0.51	0.89	0.76	0.64	0.76	0.76	0.51	0.51	0.76	0.64	0.64	0.51	0.38	1.52	1.65	1.65	2.03	1.78
39	EF1ACPU39	8	7	9	8	9	11	7	8	-	0.38	0.25	1.14	0.76	0.89	1.02	0.89	1.02	1.27	0.76	1.02	1.02	0.89	1.14	1.02	0.89	1.90	2.03	2.03	2.41	2.16
40	EF1ACPU40	9	8	10	9	10	12	8	9	3	-	0.38	1.27	0.89	1.02	1.14	1.02	1.14	1.40	0.89	1.14	1.14	1.02	1.27	1.14	1.02	2.03	2.16	2.16	2.54	2.28
41	EF1ACPU41	8	7	9	8	9	11	7	8	2	3	-	1.14	0.76	0.89	1.02	0.89	1.02	1.27	0.76	1.02	1.02	0.89	1.14	1.02	0.89	1.90	2.03	2.03	2.41	2.16
42	EF1ACPU42	5	4	6	5	6	8	4	5	9	10	9	-	0.64	1.02	0.89	0.76	0.89	0.89	0.64	0.64	0.89	0.76	0.76	0.64	0.51	1.78	1.90	1.90	2.28	2.03
43	EF1ACPU43	4	3	5	4	5	7	3	4	6	7	6	5	-	0.64	0.51	0.38	0.51	0.76	0.25	0.51	0.51	0.38	0.64	0.51	0.38	1.40	1.52	1.52	1.90	1.65
44	EF1ACPU44	7	6	8	7	8	10	6	7	7	8	7	8	5	-	0.64	0.76	0.89	1.14	0.64	0.89	0.89	0.76	1.02	0.89	0.76	1.78	1.90	1.90	2.28	2.03
45	EF1ACPU45	6	5	7	6	7	9	5	6	8	9	8	7	4	5	-	0.64	0.76	1.02	0.51	0.76	0.76	0.64	0.89	0.76	0.64	1.65	1.78	1.78	2.16	1.90
46	EF1ACPU46	5	4	6	5	6	8	4	5	7	8	7	6	3	6	5	-	0.64	0.89	0.38	0.64	0.64	0.51	0.76	0.64	0.51	1.52	1.65	1.65	1.78	1.78
47	EF1ACPU47	6	5	7	4	7	9	5	6	8	9	8	7	4	7	6	5	-	1.02	0.51	0.76	0.76	0.64	0.89	0.76	0.64	1.65	1.78	1.78	2.16	1.90
48	EF1ACPU48	6	5	7	6	7	9	5	6	10	11	10	7	6	9	8	7	8	-	0.76	0.51	0.76	0.64	0.64	0.76	0.64	1.90	2.03	2.03	2.41	2.16
49	EF1ACPU49	4	3	5	4	5	7	3	4	6	7	6	5	2	5	4	3	4	6	-	0.51	0.25	0.13	0.64	0.51	0.38	1.40	1.52	1.52	1.90	1.65
50	EF1ACPU50	4	3	3	4	5	7	3	4	8	9	8	5	4	7	6	5	6	4	4	-	0.76	0.64	0.38	0.51	0.38	1.65	1.78	1.78	2.16	1.90
51	EF1ACPU51	6	5	7	6	7	9	5	6	8	9	8	7	4	7	6	5	6	6	2	6	-	0.13	0.89	0.76	0.64	1.65	1.78	1.78	2.16	1.90
52	EF1ACPU52	5	4	6	5	6	8	4	5	7	8	7	6	3	6	5	4	5	5	1	5	1	-	0.76	0.64	0.51	1.52	1.65	1.65	2.03	1.78
53	EF1ACPU53	5	4	6	5	6	8	4	5	9	10	9	6	5	8	7	6	7	5	5	3	7	6	-	0.64	0.51	1.777	1.90	1.90	2.28	2.03
54	EF1ACPU54	4	3	5	4	5	7	3	4	8	9	8	5	4	7	6	5	6	6	4	4	6	5	5	-	0.38	1.65	1.78	1.78	2.16	1.90
55	EF1ACPU55	3	2	4	3	4	6	2	3	7	8	7	4	3	6	5	4	5	5	3	3	5	4	4	3	-	1.52	1.65	1.65	2.03	1.78
56	EF1ACPI01	13	12	12	13	14	16	12	12	15	16	15	14	11	14	13	12	13	15	11	13	13	12	14	13	12	-	0.13	0.13	0.51	0.25
57	EF1ACPI02	14	13	13	14	15	17	13	13	16	17	16	15	12	15	14	13	14	16	12	14	14	13	15	14	13	1	-	0.25	0.64	0.38
58	EF1ACPI03	14	13	13	14	15	17	13	13	16	17	16	15	12	15	14	13	14	16	12	14	14	13	15	14	13	1	2	-	0.64	0.38
59	EF1ACPI04	17	16	16	17	18	20	16	16	19	20	19	18	15	18	17	14	17	19	15	17	17	16	18	17	16	4	5	5	-	0.51

60	EF1ACPI05	15	14	14	15	16	18	14	14	17	18	17	16	13	16	15	14	15	17	13	15	15	14	16	15	14	2	3	3	4	-
61	EF1ACPI06	17	16	16	17	18	20	16	16	19	20	19	18	15	18	17	16	17	19	15	17	17	16	18	17	16	4	5	5	6	4
62	EF1ACPI07	15	14	14	15	16	18	14	14	17	18	17	16	13	16	15	14	15	17	13	15	15	14	16	15	14	2	3	3	4	2
63	EF1ACPI08	16	15	15	16	17	19	15	15	18	19	18	17	14	17	16	15	16	18	14	16	16	15	17	16	15	3	4	4	5	3
64	EF1ACPI09	15	14	14	15	16	18	14	14	17	18	17	16	13	16	15	14	15	17	13	15	15	14	16	15	14	2	3	3	4	2
65	EF1ACPI10	17	16	16	17	18	20	16	16	19	20	19	18	15	18	17	16	17	19	15	17	17	16	18	17	16	4	5	5	6	4
66	EF1ACPI11	10	9	11	10	11	13	9	10	12	13	12	11	8	11	10	9	10	12	8	10	10	9	11	10	9	9	10	10	13	11
67	EF1ACPI12	14	13	13	14	15	17	13	13	16	17	16	15	12	15	14	13	14	16	12	14	14	13	15	14	13	1	2	2	3	1
68	EF1ACPI13	15	14	14	15	16	18	14	14	17	18	17	16	13	16	15	14	15	17	13	15	15	14	16	15	14	2	3	3	4	2
69	EF1ACPI14	15	14	14	15	16	18	14	14	17	18	17	16	13	16	15	14	15	17	13	15	15	14	16	15	14	2	3	3	4	2
70	EF1ACPI15	16	15	15	16	17	19	15	15	18	19	18	17	14	17	16	15	16	18	14	16	16	15	17	16	15	3	4	4	5	3
71	EF1ACPI16	14	13	13	14	15	17	13	13	16	17	16	15	12	15	14	13	14	16	12	14	14	13	15	14	13	3	4	4	5	3
72	EF1ACPI17	20	19	19	20	21	23	19	19	22	23	22	21	18	21	20	19	20	22	18	20	20	19	21	20	19	7	8	8	9	7
73	EF1ACPI18	44	43	45	43	45	47	43	43	44	45	44	45	42	43	44	43	41	45	41	44	42	41	45	44	43	35	36	36	39	37
74	EF1ACPI19	15	14	14	15	16	18	14	14	17	18	17	16	13	16	15	14	15	17	13	15	15	14	16	15	14	2	3	3	4	2
75	EF1ACPI20	18	17	17	18	19	21	17	17	20	21	20	19	16	19	18	17	18	20	16	18	18	17	19	18	17	5	6	6	7	5
76	EF1ACPI21	15	14	14	15	16	18	14	14	17	18	17	16	13	16	15	14	15	17	13	15	15	14	16	15	14	2	3	3	4	2
77	EF1ACPI22	19	18	18	19	20	22	18	18	21	22	21	20	17	20	19	18	19	21	17	19	19	18	20	19	18	6	7	7	10	8
78	EF1ACPI23	17	16	16	17	18	20	16	16	19	20	19	18	15	18	17	16	17	19	15	17	17	16	18	17	16	4	5	5	8	6
79	EF1ACPI24	15	14	14	15	16	18	14	14	17	16	17	16	13	16	15	14	15	17	13	15	15	14	16	15	14	2	3	3	4	2
80	EF1ACPI25	15	14	14	15	16	18	14	14	17	18	17	16	13	16	15	14	15	17	13	15	15	14	16	15	14	2	3	3	4	2
81	EF1ACPI26	20	19	19	20	21	23	19	19	22	23	22	21	18	21	20	19	20	22	18	20	20	19	21	20	19	7	8	8	9	7
82	EF1ACPI27	18	17	17	18	19	21	17	17	20	21	20	19	16	19	18	17	18	20	16	18	18	17	19	18	17	5	6	6	7	5
83	EF1ACPI28	15	14	14	15	16	18	14	14	17	18	17	16	13	16	15	14	15	17	13	15	15	14	16	15	14	2	3	3	4	2
84	EF1ACPI29	15	14	14	15	16	18	14	14	17	18	17	16	13	16	15	14	15	17	13	15	15	14	16	15	14	2	3	3	4	2
85	EF1ACPI30	17	16	16	17	18	20	16	16	19	20	19	18	15	18	17	16	17	19	15	17	17	16	18	17	16	4	5	5	8	6
86	EF1ACPI31	15	14	14	15	16	18	14	14	17	18	17	16	13	16	15	14	15	17	13	15	15	14	16	15	14	2	3	3	6	4
87	EF1ACPI32	16	15	15	16	17	19	15	15	18	19	18	17	14	17	16	15	16	18	14	16	16	15	17	16	15	3	4	4	5	3
88	EF1ACPI33	15	14	14	15	16	18	14	14	17	18	17	16	13	16	15	14	15	17	13	15	15	14	16	15	14	2	3	3	4	2
89	EF1ACPI34	16	15	15	16	17	19	15	15	18	19	18	17	14	17	16	15	16	18	14	16	16	15	17	16	15	3	4	4	5	3
90	EF1ACPI35	16	15	15	16	17	19	15	15	18	19	18	17	14	17	16	15	16	18	14	16	16	15	17	16	15	3	4	4	5	3
91	EF1ACPI36	15	14	14	15	16	18	14	14	17	18	17	16	13	16	15	14	15	17	13	15	15	14	16	15	14	2	3	3	4	2
92	EF1ACPI37	17	16	16	17	18	20	16	16	19	20	19	18	15	18	17	16	17	19	15	17	17	16	18	17	16	4	5	5	6	4
93	EF1ACPI38	17	16	16	17	18	20	16	16	19	20	19	18	15	18	17	16	17	19	15	17	17	16	18	17	16	4	5	5	6	4
94	EF1ACPI39	16	15	15	16	17	19	15	15	18	19	18	17	14	17	16	15	16	18	14	16	16	15	17	16	15	3	4	4	5	3
95	EF1ACPI40	20	19	19	20	21	23	19	19	22	23	22	21	18	21	20	19	20	22	18	20	20	19	21	20	19	7	8	8	9	7
96	EF1ACPI41	16	15	15	16	17	19	15	15	18	19	18	17	14	17	16	15	16	18	14	16	16	15	17	16	15	3	4	4	5	3
97	EF1ACPI42	19	18	18	19	20	22	18	18	21	22	21	20	17	20	19	18	19	21	17	19	19	18	20	19	18	6	7	7	8	6
98	PI1	17	16	16	17	18	20	16	16	19	20	19	18	15	18	17	16	17	19	15	17	17	16	18	17	16	4	5	5	6	4
99	PI2	19	18	18	19	20	22	18	18	21	22	21	20	17	20	19	18	19	21	17	19	19	18	20	19	18	6	7	7	8	6
100	PI3	16	15	15	16	17	19	15	15	18	19	18	17	14	17	16	15	16	18	14	16	16	15	17	16	15	3	4	4	5	3
101	PI4	18	17	17	18	19	21	17	17	20	21	20	19	16	19	18	17	18	20	16	18	18	17	19	18	17	6	7	7	8	6
102	PI5	15	14	14	15	16	18	14	14	17	17	17	16	13	16	15	14	15	17	13	15	15	14	16	15	14	4	5	5	6	4
103	PI6	18	17	17	18	19	21	17	17	20	21	20	19	16	19	18	17	18	20	16	18	18	17	19	18	17	5	6	6	7	5
104	PU1	7	6	8	7	8	10	6	7	9	10	9	8	5	8	7	6	5	9	5	7	7	6	8	7	6	13	14	14	17	15
105	PU2	6	5	7	6	7	9	5	6	10	11	10	7	6	9	8	7	8	8	6	6	8	7	7	6	5	15	16	16	19	17
106	PU3	4	3	5	4	5	7	3	4	8	9	8	5	4	7	6	5	6	5	4	4	6	5	5	4	3	13	14	14	17	15
107	PU4	6	5	7	6	7	9	5	6	10	11	10	7	6	9	8	7	8	8	6	6	8	7	7	6	5	15	16	16	19	17
108	PU5	4	3	5	4	5	7	3	4	8	9	8	5	4	7	6	5	6	6	4	4	6	5	5	4	3	13	14	14	17	15
109	PU6	6	5	7	6	7	9	5	6	4	5	4	7	4	5	6	5	6	8	4	6	6	5	7	6	5	13	14	14	17	15
110	PU7	7	6	8	7	8	10	6	7	9	10	9	8	3	8	7	6	7	9	5	7	7	6	8	7	6	14	15	15	18	16

		61	62	63	64	65	66	67	68	69	70	71	72	73	74	75	76	77	78	79	80	81	82	83	84	85	86	87	88	89	90
1	EF1ACPU01	1.90	1.65	1.78	1.65	1.90	1.02	1.52	1.65	1.65	1.78	1.52	2.28	5.33	1.65	2.03	1.65	2.16	1.90	1.65	1.65	2.28	2.03	1.65	1.65	1.90	1.65	1.78	1.65	1.78	1.78
2	EF1ACPU02	2.03	1.78	1.90	1.78	2.03	1.14	1.65	1.78	1.78	1.90	1.65	2.41	5.46	1.78	2.16	1.78	2.28	2.03	1.78	1.78	2.41	2.16	1.78	1.78	2.03	1.78	1.90	1.78	1.90	1.90
3	EF1ACPU03	2.16	1.90	2.03	1.90	2.16	1.27	1.78	1.90	1.90	2.03	1.78	2.54	5.58	1.90	2.28	1.90	2.41	2.16	1.90	1.90	2.54	2.28	1.90	1.90	2.16	1.90	2.03	1.90	2.03	2.03
4	EF1ACPU04	2.03	1.78	1.90	1.78	2.03	1.14	1.65	1.78	1.78	1.90	1.65	2.41	5.46	1.78	2.16	1.78	2.28	2.03	1.78	1.78	2.41	2.16	1.78	1.78	2.03	1.78	1.90	1.78	1.90	1.90
5	EF1ACPU05	2.03	1.78	1.90	1.78	2.03	1.14	1.65	1.78	1.78	1.90	1.65	2.41	5.46	1.78	2.16	1.78	2.28	2.03	1.78	1.78	2.41	2.16	1.78	1.78	2.03	1.78	1.90	1.78	1.90	1.90
6	EF1ACPU06	2.41	2.16	2.28	2.16	2.41	1.52	2.03	2.16	2.16	2.28	2.03	2.79	5.71	2.16	2.54	2.16	2.67	2.41	2.16	2.16	2.79	2.54	2.16	2.16	2.41	2.16	2.28	2.16	2.28	2.28
7	EF1ACPU07	2.16	1.90	2.03	1.90	2.16	1.27	1.78	1.90	1.90	2.03	1.78	2.54	5.46	1.90	2.28	1.90	2.41	2.16	1.90	1.90	2.54	2.28	1.90	1.90	2.16	1.90	2.03	1.90	2.03	2.03
8	EF1ACPU08	2.16	1.90	2.03	1.90	2.16	1.27	1.78	1.90	1.90	2.03	1.78	2.54	5.46	1.90	2.28	1.90	2.41	2.16	1.90	1.90	2.54	2.28	1.90	1.90	2.16	1.90	2.03	1.90	2.03	2.03
9	EF1ACPU09	2.28	2.03	2.16	2.03	2.28	1.40	1.90	2.03	2.03	2.16	1.90	2.67	5.58	2.03	2.41	2.03	2.54	2.28	2.03	2.03	2.67	2.41	2.03	2.03	2.28	2.03	2.16	2.03	2.16	2.16
10	EF1ACPU10	2.03	1.78	1.90	1.78	2.03	1.14	1.65	1.78	1.78	1.90	1.65	2.41	5.46	1.78	2.16	1.78	2.28	2.03	1.78	1.78	2.41	2.16	1.78	1.78	2.03	1.78	1.90	1.78	1.90	1.90
11	EF1ACPU11	2.28	2.03	2.16	2.03	2.28	1.40	1.90	2.03	2.03	2.16	1.90	2.67	5.33	2.03	2.41	2.03	2.54	2.28	2.03	2.03	2.67	2.41	2.03	2.03	2.28	2.03	2.16	2.03	2.16	2.16
12	EF1ACPU12	2.41	2.16	2.28	2.16	2.41	1.52	2.03	2.16	2.16	2.28	2.03	2.79	5.58	2.16	2.54	2.16	2.67	2.41	2.16	2.16	2.79	2.54	2.16	2.16	2.41	2.16	2.28	2.16	2.28	2.28
13	EF1ACPU13	2.03	1.78	1.90	1.78	2.03	1.14	1.65	1.78	1.78	1.90	1.65	2.41	5.20	1.78	2.16	1.78	2.28	2.03	1.78	1.78	2.41	2.16	1.78	1.78	2.03	1.78	1.90	1.78	1.90	1.90
14	EF1ACPU14	1.90	1.65	1.78	1.65	1.90	1.02	1.52	1.65	1.65	1.78	1.52	2.28	5.33	1.65	2.03	1.65	2.16	1.90	1.65	1.65	2.28	2.03	1.65	1.65	1.90	1.65	1.78	1.65	1.78	1.78
15	EF1ACPU15	1.90	1.65	1.78	1.65	1.90	1.02	1.52	1.65	1.65	1.78	1.52	2.28	5.33	1.65	2.03	1.65	2.16	1.90	1.65	1.65	2.28	2.03	1.65	1.65	1.90	1.65	1.78	1.65	1.78	1.78
16	EF1ACPU16	2.03	1.78	1.90	1.78	2.03	1.14	1.65	1.78	1.78	1.90	1.65	2.41	5.46	1.78	2.16	1.78	2.28	2.03	1.78	1.78	2.41	2.16	1.78	1.78	2.03	1.78	1.90	1.78	1.90	1.90
17	EF1ACPU17	2.03	1.78	1.90	1.78	2.03	1.14	1.65	1.78	1.78	1.90	1.65	2.41	5.46	1.78	2.16	1.78	2.28	2.03	1.78	1.78	2.41	2.16	1.78	1.78	2.03	1.78	1.90	1.78	1.90	1.90
18	EF1ACPU18	2.03	1.78	1.90	1.78	2.03	1.14	1.65	1.78	1.78	1.90	1.65	2.41	5.46	1.78	2.16	1.78	2.28	2.03	1.78	1.78	2.41	2.16	1.78	1.78	2.03	1.78	1.90	1.78	1.90	1.90
19	EF1ACPU19	1.90	1.65	1.78	1.65	1.90	1.02	1.52	1.65	1.65	1.78	1.52	2.28	5.08	1.65	2.03	1.65	2.16	1.90	1.65	1.65	2.28	2.03	1.65	1.65	1.90	1.65	1.78	1.65	1.78	1.78
20	EF1ACPU20	2.41	2.16	2.28	2.16	2.41	1.52	2.03	2.16	2.16	2.28	2.03	2.79	5.84	2.16	2.54	2.16	2.67	2.41	2.16	2.16	2.79	2.54	2.16	2.16	2.41	2.16	2.28	2.16	2.28	2.28
21	EF1ACPU21	2.54	2.28	2.41	2.28	2.54	1.65	2.16	2.28	2.28	2.41	2.16	2.92	5.58	2.28	2.67	2.28	2.79	2.54	2.28	2.28	2.92	2.67	2.28	2.28	2.54	2.28	2.41	2.28	2.41	2.41
22	EF1ACPU22	2.03	1.78	1.90	1.78	2.03	1.14	1.65	1.78	1.78	1.90	1.65	2.41	5.46	1.78	2.16	1.78	2.28	2.03	1.78	1.78	2.41	2.16	1.78	1.78	2.03	1.78	1.90	1.78	1.90	1.90
23	EF1ACPU23	2.03	1.78	1.90	1.78	2.03	1.14	1.65	1.78	1.78	1.90	1.65	2.41	5.46	1.78	2.16	1.78	2.28	2.03	1.78	1.78	2.41	2.16	1.78	1.78	2.03	1.78	1.90	1.78	1.90	1.90
24	EF1ACPU24	2.28	2.03	2.16	2.03	2.28	1.40	1.90	2.03	2.03	2.16	1.90	2.67	5.71	2.03	2.41	2.03	2.54	2.28	2.03	2.03	2.54	2.41	2.03	2.03	2.28	2.03	2.16	2.03	2.16	2.16
25	EF1ACPU25	2.03	1.78	1.90	1.78	2.03	1.14	1.65	1.78	1.78	1.90	1.65	2.41	5.20	1.78	2.16	1.78	2.28	2.03	1.78	1.78	2.41	2.16	1.78	1.78	2.03	1.78	1.90	1.78	1.90	1.90
26	EF1ACPU26	2.16	1.90	2.03	1.90	2.16	1.27	1.78	1.90	1.90	2.03	1.78	2.54	5.08	1.90	2.28	1.90	2.41	2.16	1.90	1.90	2.54	2.28	1.90	1.90	2.16	1.90	2.03	1.90	2.03	2.03
27	EF1ACPU27	2.16	1.90	2.03	1.90	2.16	1.27	1.78	1.90	1.90	2.03	1.78	2.54	5.58	1.90	2.28	1.90	2.41	2.16	1.90	1.90	2.54	2.28	1.90	1.90	2.16	1.90	1.78	1.90	2.03	2.03
28	EF1ACPU28	2.16	1.90	2.03	1.90	2.16	1.27	1.78	1.90	1.90	2.03	1.78	2.54	5.58	1.90	2.28	1.90	2.41	2.16	1.90	1.90	2.54	2.28	1.90	1.90	2.16	1.90	2.03	1.90	2.03	2.03
29	EF1ACPU29	2.16	1.90	2.03	1.90	2.16	1.27	1.78	1.90	1.90	2.03	1.78	2.54	5.58	1.90	2.28	1.90	2.41	2.16	1.90	1.90	2.54	2.28	1.90	1.90	2.16	1.90	2.03	1.90	2.03	2.03
30	EF1ACPU30	2.03	1.78	1.90	1.78	2.03	1.14	1.65	1.78	1.78	1.90	1.65	2.41	5.46	1.78	2.16	1.78	2.28	2.03	1.78	1.78	2.41	2.16	1.78	1.78	2.03	1.78	1.90	1.78	1.90	1.90
31	EF1ACPU31	2.16	1.90	2.03	1.90	2.16	1.27	1.78	1.90	1.90	2.03	1.78	2.54	5.58	1.90	2.28	1.90	2.41	2.16	1.90	1.90	2.54	2.28	1.90	1.90	2.16	1.90	2.03	1.90	2.03	2.03
32	EF1ACPU32	2.03	1.78	1.90	1.78	2.03	1.14	1.65	1.78	1.78	1.90	1.65	2.41	5.46	1.78	2.16	1.78	2.28	2.03	1.78	1.78	2.41	2.16	1.78	1.78	2.03	1.78	1.90	1.78	1.90	1.90
33	EF1ACPU33	2.03	1.78	1.90	1.78	2.03	1.40	1.65	1.78	1.78	1.90	1.65	2.41	5.71	1.78	2.16	1.78	2.28	2.03	1.78	1.78	2.41	2.16	1.78	1.78	2.03	1.78	1.90	1.78	1.90	1.90
34	EF1ACPU34	2.16	1.90	2.03	1.90	2.16	1.27	1.78	1.90	1.90	2.03	1.78	2.54	5.46	1.90	2.28	1.90	2.41	2.16	1.90	1.90	2.54	2.28	1.90	1.90	2.16	1.90	2.03	1.90	2.03	2.03
35	EF1ACPU35	2.28	2.03	2.16	2.03	2.28	1.40	1.90	2.03	2.03	2.16	1.90	2.67	5.71	2.03	2.41	2.03	2.54	2.28	2.03	2.03	2.67	2.41	2.03	2.03	2.28	2.03	2.16	2.03	2.16	2.16
36	EF1ACPU36	2.54	2.28	2.41	2.28	2.54	1.65	2.16	2.28	2.28	2.41	2.16	2.92	5.96	2.28	2.67	2.28	2.79	2.54	2.28	2.28	2.92	2.67	2.28	2.28	2.54	2.28	2.41	2.28	2.41	2.41
37	EF1ACPU37	2.03	1.78	1.90	1.78	2.03	1.14	1.65	1.78	1.78	1.90	1.65	2.41	5.46	1.78	2.16	1.78	2.28	2.03	1.78	1.78	2.41	2.16	1.78	1.78	2.03	1.78	1.90	1.78	1.90	1.90
38	EF1ACPU38	2.03	1.78	1.90	1.78	2.03	1.27	1.65	1.78	1.78	1.90	1.65	2.41	5.46	1.78	2.16	1.78	2.28	2.03	1.78	1.78	2.41	2.16	1.78	1.78	2.03	1.78	1.90	1.78	1.90	1.90
39	EF1ACPU39	2.41	2.16	2.28	2.16	2.41	1.52	2.03	2.16	2.16	2.28	2.03	2.79	5.58	2.16	2.54	2.16	2.67	2.41	2.16	2.16	2.79	2.54	2.16	2.16	2.41	2.16	2.28	2.16	2.28	2.28
40	EF1ACPU40	2.54	2.28	2.41	2.28	2.54	1.65	2.16	2.28	2.28	2.41	2.16	2.92	5.71	2.28	2.67	2.28	2.79	2.54	2.03	2.28	2.92	2.67	2.28	2.28	2.54	2.28	2.41	2.28	2.41	2.41
41	EF1ACPU41	2.41	2.16	2.28	2.16	2.41	1.52	2.03	2.16	2.16	2.28	2.03	2.79	5.58	2.16	2.54	2.16	2.67	2.41	2.16	2.16	2.79	2.54	2.16	2.16	2.41	2.16	2.28	2.16	2.28	2.28
42	EF1ACPU42	2.28	2.03	2.16	2.03	2.28	1.40	1.90	2.03	2.03	2.16	1.90	2.67	5.71	2.03	2.41	2.03	2.54	2.28	2.03	2.03	2.67	2.41	2.03	2.03	2.28	2.03	2.16	2.03	2.16	2.16
43	EF1ACPU43	1.90	1.65	1.78	1.65	1.90	1.02	1.52	1.65	1.65	1.78	1.52	2.28	5.33	1.65	2.03	1.65	2.16	1.90	1.65	1.65	2.28	2.03	1.65	1.65	1.90	1.65	1.78	1.65	1.78	1.78
44	EF1ACPU44	2.28	2.03	2.16	2.03	2.28	1.40	1.90	2.03	2.03	2.16	1.90	2.67	5.46	2.03	2.41	2.03	2.54	2.28	2.03	2.03	2.67	2.41	2.03	2.03	2.28	2.03	2.16	2.03	2.16	2.16
45	EF1ACPU45	2.16	1.90	2.03	1.90	2.16	1.27	1.78	1.90	1.90	2.03	1.78	2.54	5.58	1.90	2.28	1.90	2.41	2.16	1.90	1.90	2.54	2.28	1.90	1.90	2.16	1.90	2.03	1.90	2.03	2.03
46	EF1ACPU46	2.03	1.78	1.90	1.78	2.03	1.14	1.65	1.78	1.78	1.90	1.65	2.41	5.46	1.78	2.16	1.78	2.28	2.03	1.78	1.78	2.41	2.16	1.78	1.78	2.03	1.78	1.90	1.78	1.90	1.90
47	EF1ACPU47	2.16	1.90	2.03	1.90	2.16	1.27	1.78	1.90	1.90	2.03	1.78	2.54	5.20	1.90	2.28	1.90	2.41	2.16	1.90	1.90	2.54	2.28	1.90	1.90	2.16	1.90	2.03	1.90	2.03	2.03
48	EF1ACPU48	2.41	2.16	2.28	2.16	2.41	1.52	2.03	2.16	2.16	2.28	2.03	2.79	5.71	2.16	2.54	2.16	2.67	2.41	2.16	2.16	2.79	2.54	2.16	2.16	2.41	2.16	2.28	2.16	2.28	2.28
49	EF1ACPU49	1.90	1.65	1.78	1.65	1.90	1.02	1.52	1.65	1.65	1.78	1.52	2.28	5.20	1.65	2.03	1.65	2.16	1.90	1.65	1.65	2.28	2.03	1.65	1.65	1.90	1.65	1.78	1.65	1.78	1.78
50	EF1ACPU50	2.16	1.90	2.03	1.90	2.16	1.27	1.78	1.90	1.90	2.03	1.78	2.54	5.58	1.90	2.28	1.90	2.41	2.16	1.90	1.90	2.54	2.28	1.90	1.90	2.16	1.90	2.03	1.90	2.03	2.03
51	EF1ACPU51	2.16	1.90	2.03	1.90	2.16	1.27	1.78	1.90	1.90	2.03	1.78	2.54	5.33	1.90	2.28	1.90	2.41	2.16	1.90	1.90	2.54	2.28	1.90	1.90	2.16	1.90	2.03	1.90	2.03	2.03
52	EF1ACPU52	2.03	1.78	1.90	1.78	2.03	1.14	1.65	1.78	1.78	1.90	1.65	2.41	5.20	1.78	2.16	1.78	2.28	2.03	1.78	1.78	2.41	2.16	1.78	1.78	2.03	1.78	1.90	1.78	1.90	1.90
53	EF1ACPU53	2.28	2.03	2.16	2.03	2.28	1.40	1.90	2.03	2.03	2.16	1.90	2.67	5.71	2.03	2.41	2.03	2.54	2.28	2.03	2.03	2.67	2.41	2.03	2.03	2.28	2.03	2.16	2.03	2.16	2.16
54	EF1ACPU54	2.16	1.90	2.03	1.90	2.16	1.27	1.78	1.90	1.90	2.03	1.78	2.54	5.58	1.90	2.28	1.90	2.41	2.16	1.90	1.90	2.54	2.28	1.90	1.90	2.16	1.90	2.03	1.90	2.03	2.03
55	EF1ACPU55	2.03	1.78	1.90	1.78	2.03	1.14	1.65	1.78	1.78	1.90	1.65	2.41	5.46	1.78	2.16	1.78	2.28	2.03	1.78	1.78	2.41	2.16	1.78	1.78	2.03	1.78	1.90	1.78	1.90	1.90
56	EF1ACPI01	0.51	0.25	0.38	0.25	0.51	1.14	0.13	0.25	0.25	0.38	0.38	0.89	4.44	0.25	0.64	0.25	0.76	0.51	0.25	0.25	0.89	0.64	0.25	0.25	0.51	0.25	0.38	0.25	0.38	0.38
57	EF1ACPI02	0.64	0.38	0.51	0.38	0.64	1.27	0.25	0.38	0.38	0.51	0.51	1.02	4.57	0.38	0.76	0.38	0.89	0.64	0.38	0.38	1.02	0.76	0.38	0.38	0.64	0.38	0.51	0.38	0.51	0.51
58	EF1ACPI03	0.64	0.38	0.51	0.38	0.64	1.27	0.25	0.38	0.38	0.51	0.51	1.02	4.57	0.38	0.76	0.38	0.89	0.64	0.38	0.38	1.02	0.76	0.38	0.38	0.64	0.38	0.51	0.38	0.51	0.51
59	EF1ACPI04	0.76	0.51	0.64	0.51	0.76	1.65	0.38	0.51	0.51	0.64	0.64	1.14	4.95	0.51	0.89	0.51	1.27	1.02	0.51	0.51	1.14	0.89	0.51	0.51	1.02	0.76	0.64	0.51	0.64	0.64

60	EF1ACPI05	0.51	0.25	0.38	0.25	0.51	1.40	0.13	0.25	0.25	0.38	0.38	0.89	4.70	0.25	0.64	0.25	1.02	0.76	0.25	0.25	0.89	0.64	0.25	0.25	0.76	0.51	0.38	0.25	0.38	0.38
61	EF1ACPI06	-	0.51	0.64	0.51	0.76	1.65	0.38	0.51	0.51	0.64	0.64	1.14	4.95	0.51	0.89	0.51	1.27	1.02	0.51	0.51	1.14	0.89	0.51	0.51	1.02	0.76	0.64	0.51	0.64	0.64
62	EF1ACPI07	4	-	0.38	0.25	0.51	1.40	0.13	0.25	0.25	0.38	0.38	0.89	4.70	0.25	0.64	0.25	1.02	0.76	0.25	0.25	0.89	0.64	0.25	0.25	0.76	0.51	0.38	0.25	0.38	0.38
63	EF1ACPI08	5	3	-	0.38	0.51	1.52	0.25	0.38	0.38	0.51	0.51	1.02	4.82	0.38	0.76	0.38	1.14	0.89	0.38	0.38	1.02	0.76	0.38	0.38	0.89	0.64	0.51	0.38	0.51	0.51
64	EF1ACPI09	4	2	3	-	0.51	1.40	0.13	0.25	0.25	0.38	0.38	0.89	4.70	0.25	0.64	0.25	1.02	0.76	0.25	0.25	0.89	0.64	0.25	0.25	0.76	0.51	0.38	0.25	0.38	0.38
65	EF1ACPI10	6	4	4	4	-	1.65	0.38	0.51	0.51	0.64	0.64	1.14	4.95	0.51	0.89	0.51	1.27	1.02	0.51	0.51	1.14	0.89	0.51	0.51	1.02	0.76	0.64	0.51	0.64	0.64
66	EF1ACPI11	13	11	12	11	13	-	1.27	1.40	1.40	1.52	1.27	2.03	4.95	1.40	1.78	1.40	1.90	1.65	1.40	1.40	2.03	1.78	1.40	1.40	1.65	1.40	1.52	1.40	1.52	1.52
67	EF1ACPI12	3	1	2	1	3	10	-	0.13	0.13	0.25	0.25	0.76	4.57	0.13	0.51	0.13	0.89	0.64	0.13	0.13	0.76	0.51	0.13	0.13	0.64	0.38	0.25	0.13	0.25	0.25
68	EF1ACPI13	4	2	3	2	4	11	1	-	0.25	0.38	0.38	0.89	4.70	0.25	0.64	0.25	1.02	0.76	0.25	0.25	0.89	0.64	0.25	0.25	0.76	0.51	0.38	0.25	0.38	0.38
69	EF1ACPI14	4	2	3	2	4	11	1	2	-	0.38	0.38	0.89	4.70	0.25	0.64	0.25	1.02	0.76	0.25	0.25	0.89	0.64	0.25	0.25	0.76	0.51	0.38	0.25	0.38	0.38
70	EF1ACPI15	5	3	4	3	5	12	2	3	3	-	0.51	1.02	4.82	0.38	0.76	0.38	1.14	0.89	0.38	0.38	1.02	0.76	0.38	0.38	0.89	0.64	0.51	0.38	0.51	0.51
71	EF1ACPI16	5	3	4	3	5	10	2	3	3	4	-	0.76	4.82	0.38	0.76	0.38	1.02	0.89	0.38	0.38	1.02	0.51	0.38	0.38	0.89	0.64	0.51	0.38	0.51	0.51
72	EF1ACPI17	9	7	8	7	9	16	6	7	7	8	6	-	5.33	0.89	1.27	0.89	1.52	1.40	0.89	0.89	1.52	1.02	0.89	0.89	1.40	1.14	1.02	0.89	1.02	1.02
73	EF1ACPI18	39	37	38	37	39	39	36	37	37	38	38	42	-	4.70	5.08	4.70	5.20	4.95	4.70	4.70	5.33	5.08	4.70	4.70	4.95	4.70	4.82	4.70	4.82	4.82
74	EF1ACPI19	4	2	3	2	4	11	1	2	2	3	3	7	37	-	0.64	0.25	1.02	0.76	0.25	0.25	0.89	0.64	0.25	0.25	0.76	0.51	0.38	0.25	0.38	0.38
75	EF1ACPI20	7	5	6	5	7	14	4	5	5	6	6	10	40	5	-	0.38	1.40	1.14	0.64	0.64	1.27	1.02	0.64	0.64	1.14	0.89	0.76	0.64	0.76	0.76
76	EF1ACPI21	4	2	3	2	4	11	1	2	2	3	3	7	37	2	3	-	1.015	0.76	0.25	0.25	0.89	0.64	0.25	0.25	0.76	0.51	0.38	0.25	0.38	0.38
77	EF1ACPI22	10	8	9	8	10	15	7	8	8	9	8	12	41	8	11	8	-	0.76	1.02	1.02	1.65	1.27	1.02	1.02	0.76	0.51	1.14	1.02	1.14	1.14
78	EF1ACPI23	8	6	7	6	8	13	5	6	6	7	7	11	39	6	9	6	6	-	0.76	0.76	1.40	1.14	0.76	0.76	0.51	0.25	0.89	0.76	0.89	0.89
79	EF1ACPI24	4	2	3	2	4	11	1	2	2	3	3	7	37	2	5	2	8	6	-	0.254	0.89	0.64	0.25	0.25	0.76	0.51	0.38	0.25	0.38	0.38
80	EF1ACPI25	4	2	3	2	4	11	1	2	2	3	3	7	37	2	5	2	8	6	2	-	0.89	0.64	0.25	0.25	0.76	0.51	0.38	0.25	0.38	0.38
81	EF1ACPI26	9	7	8	7	9	16	6	7	7	8	8	12	42	7	10	7	13	11	7	7	-	1.27	0.89	0.89	1.40	1.14	1.02	0.89	1.02	1.02
82	EF1ACPI27	7	5	6	5	7	14	4	5	5	6	4	8	40	5	8	5	10	9	5	5	10	-	0.64	0.64	1.14	0.89	0.76	0.64	0.76	0.51
83	EF1ACPI28	4	2	3	2	4	11	1	2	2	3	3	7	37	2	5	2	8	6	2	2	7	5	-	0.25	0.76	0.51	0.38	0.25	0.38	0.38
84	EF1ACPI29	4	2	3	2	4	11	1	2	2	3	3	7	37	2	5	2	8	6	2	2	7	5	2	-	0.76	0.51	0.38	0.25	0.38	0.38
85	EF1ACPI30	8	6	7	6	8	13	5	6	6	7	7	11	39	6	9	6	6	4	6	6	11	9	6	6	-	0.25	0.89	0.76	0.89	0.89
86	EF1ACPI31	6	4	5	4	6	11	3	4	4	5	5	9	37	4	7	4	4	2	4	4	9	7	4	4	2	-	0.64	0.51	0.64	0.64
87	EF1ACPI32	5	3	4	3	5	12	2	3	3	4	4	8	38	3	6	3	9	7	3	3	8	6	3	3	7	5	-	0.38	0.51	0.51
88	EF1ACPI33	4	2	3	2	4	11	1	2	2	3	3	7	37	2	5	2	8	6	2	2	7	5	2	2	6	4	3	-	0.381	0.38
89	EF1ACPI34	5	3	4	3	5	12	2	3	3	4	4	8	38	3	6	3	9	7	3	3	8	6	3	3	7	5	4	3	-	0.51
90	EF1ACPI35	5	3	4	3	5	12	2	3	3	4	4	8	38	3	6	3	9	7	3	3	8	4	3	3	7	5	4	3	4	-
91	EF1ACPI36	4	2	3	2	4	11	1	2	2	3	3	7	37	2	5	2	8	6	2	2	7	5	2	2	6	4	3	2	3	3
92	EF1ACPI37	6	4	5	4	6	13	3	4	4	5	5	9	39	4	7	4	10	8	4	4	9	7	4	4	8	6	5	4	5	5
93	EF1ACPI38	6	4	5	4	6	13	3	4	4	5	5	9	39	4	7	4	10	8	4	4	9	7	4	4	8	6	5	4	5	5
94	EF1ACPI39	5	3	4	3	5	12	2	3	3	4	4	8	38	3	6	3	9	7	3	3	8	6	3	3	7	5	4	3	4	4
95	EF1ACPI40	9	7	8	7	9	16	6	7	7	8	8	12	42	7	8	7	13	11	7	7	12	10	7	7	11	9	8	7	8	8
96	EF1ACPI41	5	3	4	3	5	12	2	3	3	4	4	8	38	3	6	3	9	7	3	3	8	6	3	3	7	5	4	3	4	4
97	EF1ACPI42	8	6	7	6	8	15	5	6	6	7	7	11	41	6	9	6	12	10	6	6	11	9	6	6	10	8	7	6	7	7
98	PI1	6	4	5	4	6	13	3	4	4	5	5	9	39	4	7	4	10	8	4	4	9	7	4	4	8	6	5	4	5	5
99	PI2	8	6	7	6	8	15	5	6	6	7	7	11	41	6	9	6	12	10	6	6	11	9	6	6	10	8	7	6	7	7
100	PI3	5	3	4	3	5	12	2	3	3	4	4	8	38	3	6	3	9	7	3	3	8	6	3	3	7	5	4	3	4	4
101	PI4	6	6	7	6	8	14	5	6	6	7	7	11	41	6	9	6	12	10	6	6	11	9	6	6	10	8	7	6	7	7
102	PI5	6	4	5	4	6	13	3	4	4	5	5	9	39	4	7	4	10	8	3	4	9	7	4	4	8	6	5	4	5	5
103	PI6	7	5	6	5	7	14	4	5	5	6	6	10	40	5	8	5	11	9	5	5	10	8	5	5	9	7	6	5	6	6
104	PU1	17	15	16	15	17	11	14	15	15	16	15	20	43	15	18	15	19	17	15	15	20	18	15	15	17	15	16	15	16	16
105	PU2	19	17	18	17	19	12	16	17	17	18	16	22	46	17	20	17	21	19	17	17	22	20	17	17	19	17	18	17	18	18
106	PU3	17	15	16	15	17	10	14	15	15	16	14	20	44	15	18	15	19	17	15	15	20	18	15	15	17	15	16	15	16	16
107	PU4	19	17	18	17	19	12	16	17	17	18	16	22	46	17	20	17	21	19	17	17	22	20	17	17	19	17	18	17	18	18
108	PU5	17	15	16	15	17	10	14	15	15	16	14	20	44	15	18	15	19	17	15	15	20	18	15	15	17	15	16	15	16	16
109	PU6	17	15	16	15	17	10	14	15	15	16	14	20	42	15	18	15	19	17	15	15	20	18	15	15	17	15	16	15	16	16
110	PU7	18	16	17	16	18	11	15	16	16	17	15	21	45	16	19	16	20	18	16	16	21	19	16	16	18	16	17	16	17	17

		91	92	93	94	95	96	97	98	99	100	101	102	103	104	105	106	107	108	109	110
1	EF1ACPU01	1.65	1.90	1.90	1.78	2.28	1.78	2.16	1.90	2.16	1.78	2.03	1.65	2.03	0.64	0.51	0.25	0.51	0.25	0.51	0.64
2	EF1ACPU02	1.78	2.03	2.03	1.90	2.41	1.90	2.28	2.03	2.28	1.90	2.16	1.78	2.16	0.76	0.64	0.38	0.64	0.38	0.64	0.76
3	EF1ACPU03	1.90	2.16	2.16	2.03	2.54	2.03	2.41	2.16	2.41	2.03	2.28	1.90	2.28	0.89	0.76	0.51	0.76	0.51	0.76	0.89
4	EF1ACPU04	1.78	2.03	2.03	1.90	2.41	1.90	2.28	2.03	2.28	1.90	2.16	1.78	2.16	0.76	0.64	0.38	0.64	0.38	0.64	0.76
5	EF1ACPU05	1.78	2.03	2.03	1.90	2.41	1.90	2.28	2.03	2.28	1.90	2.16	1.78	2.16	0.76	0.64	0.38	0.64	0.38	0.64	0.76
6	EF1ACPU06	2.16	2.41	2.41	2.28	2.67	2.28	2.67	2.41	2.67	2.28	2.54	2.16	2.54	0.64	1.27	1.02	1.27	1.02	1.02	1.14
7	EF1ACPU07	1.90	2.16	2.16	2.03	2.54	2.03	2.41	2.16	2.41	2.03	2.28	1.90	2.28	0.64	0.76	0.51	0.76	0.51	0.76	0.89
8	EF1ACPU08	1.90	2.16	2.16	2.03	2.54	2.03	2.41	2.16	2.41	2.03	2.28	1.90	2.28	0.64	0.76	0.51	0.76	0.51	0.76	0.89
9	EF1ACPU09	2.03	2.28	2.28	2.16	2.67	2.16	2.54	2.28	2.54	2.16	2.41	2.03	2.41	0.76	0.89	0.64	0.89	0.64	0.89	1.02
10	EF1ACPU10	1.78	2.03	2.03	1.90	2.41	1.90	2.28	2.03	2.28	1.90	2.16	1.78	2.16	0.76	0.64	0.38	0.64	0.38	0.64	0.76
11	EF1ACPU11	2.03	2.28	2.28	2.16	2.67	2.16	2.54	2.28	2.54	2.16	2.41	2.03	2.41	0.76	1.14	0.89	1.14	0.89	0.89	1.02
12	EF1ACPU12	2.16	2.41	2.41	2.03	2.79	2.28	2.67	2.41	2.67	2.28	2.54	2.16	2.54	1.14	1.27	1.02	1.27	1.02	1.02	1.14
13	EF1ACPU13	1.78	2.03	2.03	1.90	2.41	1.90	2.28	2.03	2.28	1.90	2.16	1.78	2.16	0.76	0.64	0.38	0.64	0.38	0.64	0.76
14	EF1ACPU14	1.65	1.90	1.78	1.78	2.16	1.65	2.03	1.90	2.16	1.78	2.03	1.65	2.03	0.64	0.76	0.51	0.76	0.51	0.51	0.64
15	EF1ACPU15	1.65	1.90	1.90	1.78	2.28	1.78	2.16	1.78	2.16	1.78	2.03	1.65	2.03	0.64	0.76	0.51	0.76	0.51	0.51	0.51
16	EF1ACPU16	1.78	2.03	2.03	1.90	2.41	1.90	2.28	2.03	2.28	1.90	2.16	1.78	2.16	0.76	0.64	0.38	0.64	0.38	0.64	0.76
17	EF1ACPU17	1.78	2.03	2.03	1.90	2.41	1.90	2.28	2.03	2.28	1.90	2.16	1.78	2.16	0.76	0.64	0.38	0.64	0.38	0.64	0.76
18	EF1ACPU18	1.78	2.03	2.03	1.90	2.41	1.90	2.28	2.03	2.28	1.90	2.16	1.78	2.16	0.76	0.64	0.38	0.64	0.38	0.64	0.76
19	EF1ACPU19	1.65	1.90	1.90	1.78	2.28	1.78	2.16	1.90	2.16	1.78	2.03	1.65	2.03	0.64	0.76	0.51	0.76	0.51	0.51	0.64
20	EF1ACPU20	2.16	2.41	2.41	2.28	2.79	2.28	2.67	2.41	2.67	2.28	2.54	2.16	2.54	1.14	1.02	0.76	1.02	0.76	1.02	1.14
21	EF1ACPU21	2.28	2.54	2.54	2.41	2.92	2.41	2.54	2.54	2.79	2.41	2.67	2.28	2.67	1.27	1.14	0.89	1.14	0.89	1.14	1.27
22	EF1ACPU22	1.78	2.03	2.03	1.90	2.41	1.90	2.28	2.03	2.28	1.90	2.16	1.78	2.16	0.76	0.64	0.38	0.64	0.38	0.64	0.76
23	EF1ACPU23	1.78	2.03	2.03	1.90	2.41	1.90	2.28	2.03	2.28	1.90	2.16	1.78	2.16	0.76	0.64	0.38	0.64	0.38	0.64	0.76
24	EF1ACPU24	2.03	2.28	2.28	2.16	2.67	2.16	2.54	2.28	2.54	2.16	2.41	2.03	2.41	1.02	0.89	0.64	0.89	0.64	0.89	1.02
25	EF1ACPU25	1.78	2.03	2.03	1.90	2.41	1.90	2.28	2.03	2.28	1.90	2.16	1.78	2.16	0.76	0.64	0.38	0.64	0.38	0.64	0.76
26	EF1ACPU26	1.90	2.16	2.16	2.03	2.54	2.03	2.41	2.16	2.41	2.03	2.28	1.90	2.28	0.89	0.76	0.51	0.76	0.51	0.76	0.89
27	EF1ACPU27	1.90	2.16	2.16	2.03	2.54	2.03	2.41	2.16	2.41	2.03	2.28	1.90	2.28	0.89	0.76	0.51	0.76	0.51	0.76	0.89
28	EF1ACPU28	1.90	2.16	2.16	2.03	2.54	2.03	2.41	2.16	2.41	2.03	2.28	1.90	2.28	0.89	0.76	0.51	0.76	0.51	0.76	0.89
29	EF1ACPU29	1.90	2.16	2.16	2.03	2.54	2.03	2.41	2.16	2.41	2.03	2.28	1.90	2.28	0.89	0.76	0.51	0.76	0.51	0.76	0.89
30	EF1ACPU30	1.78	2.03	2.03	1.90	2.41	1.90	2.28	2.03	2.28	1.90	2.16	1.78	2.16	0.76	0.64	0.38	0.64	0.38	0.64	0.76
31	EF1ACPU31	1.90	2.16	2.16	2.03	2.54	2.03	2.41	2.16	2.41	2.03	2.28	1.90	2.28	0.89	0.76	0.51	0.76	0.51	0.76	0.89
32	EF1ACPU32	1.78	2.03	2.03	1.90	2.41	1.90	2.28	2.03	2.28	1.90	2.16	1.78	2.16	0.76	0.64	0.38	0.64	0.38	0.64	0.76
33	EF1ACPU33	1.78	2.03	2.03	1.90	2.41	1.90	2.28	2.03	2.28	1.90	2.16	1.78	2.16	1.02	0.89	0.64	0.89	0.64	0.89	1.02
34	EF1ACPU34	1.90	2.16	2.16	2.03	2.54	2.03	2.41	2.16	2.41	2.03	2.28	1.90	2.28	0.89	0.76	0.51	0.76	0.51	0.76	0.89
35	EF1ACPU35	2.03	2.28	2.28	2.16	2.67	2.16	2.54	2.28	2.54	2.16	2.41	2.03	2.41	1.02	0.89	0.64	0.89	0.64	0.89	1.02
36	EF1ACPU36	2.28	2.54	2.54	2.41	2.92	2.41	2.79	2.54	2.79	2.41	2.67	2.28	2.67	1.27	1.14	0.89	1.14	0.89	1.14	1.27
37	EF1ACPU37	1.78	2.03	2.03	1.90	2.41	1.90	2.28	2.03	2.28	1.90	2.16	1.78	2.16	0.76	0.64	0.38	0.64	0.38	0.64	0.76
38	EF1ACPU38	1.78	2.03	2.03	1.90	2.41	1.90	2.28	2.03	2.28	1.90	2.16	1.78	2.16	0.89	0.76	0.51	0.76	0.51	0.76	0.89
39	EF1ACPU39	2.16	2.41	2.41	2.28	2.79	2.28	2.67	2.41	2.67	2.28	2.54	2.16	2.54	1.14	1.27	1.02	1.27	1.02	0.51	1.14
40	EF1ACPU40	2.28	2.54	2.54	2.41	2.92	2.41	2.79	2.54	2.79	2.41	2.67	2.16	2.67	1.27	1.40	1.14	1.40	1.14	0.64	1.27
41	EF1ACPU41	2.16	2.41	2.41	2.28	2.79	2.28	2.67	2.41	2.67	2.28	2.54	2.16	2.54	1.14	1.27	1.02	1.27	1.02	0.51	1.14
42	EF1ACPU42	2.03	2.28	2.28	2.16	2.67	2.16	2.54	2.28	2.54	2.16	2.41	2.03	2.41	1.02	0.89	0.64	0.89	0.64	0.89	1.02
43	EF1ACPU43	1.65	1.90	1.90	1.78	2.28	1.78	2.16	1.90	2.16	1.78	2.03	1.65	2.03	0.64	0.76	0.51	0.76	0.51	0.51	0.38
44	EF1ACPU44	2.03	2.28	2.28	2.16	2.67	2.16	2.54	2.28	2.54	2.16	2.41	2.03	2.41	1.02	1.14	0.89	1.14	0.89	0.64	1.02
45	EF1ACPU45	1.90	2.16	2.16	2.03	2.54	2.03	2.41	2.16	2.41	2.03	2.28	1.90	2.28	0.89	1.02	0.76	1.02	0.76	0.76	0.89
46	EF1ACPU46	1.78	2.03	2.03	1.90	2.41	1.90	2.28	2.03	2.28	1.90	2.16	1.78	2.16	0.76	0.89	0.64	0.89	0.64	0.64	0.76
47	EF1ACPU47	1.90	2.16	2.16	2.03	2.54	2.03	2.41	2.16	2.41	2.03	2.28	1.90	2.28	0.64	1.02	0.76	1.02	0.76	0.76	0.89
48	EF1ACPU48	2.16	2.41	2.41	2.28	2.79	2.28	2.67	2.41	2.67	2.28	2.54	2.16	2.54	1.14	1.02	0.64	1.02	0.76	1.02	1.14
49	EF1ACPU49	1.65	1.90	1.90	1.78	2.28	1.78	2.16	1.90	2.16	1.78	2.03	1.65	2.03	0.64	0.76	0.51	0.76	0.51	0.51	0.64
50	EF1ACPU50	1.90	2.16	2.16	2.03	2.54	2.03	2.41	2.16	2.41	2.03	2.28	1.90	2.28	0.89	0.76	0.51	0.76	0.51	0.76	0.89
51	EF1ACPU51	1.90	2.16	2.16	2.03	2.54	2.03	2.41	2.16	2.41	2.03	2.28	1.90	2.28	0.89	1.02	0.76	1.02	0.76	0.76	0.89
52	EF1ACPU52	1.78	2.03	2.03	1.90	2.41	1.90	2.28	2.03	2.28	1.90	2.16	1.78	2.16	0.76	0.89	0.64	0.89	0.64	0.64	0.76
53	EF1ACPU53	2.03	2.28	2.28	2.16	2.67	2.16	2.54	2.28	2.54	2.16	2.41	2.03	2.41	1.02	0.89	0.64	0.89	0.64	0.89	1.02
54	EF1ACPU54	1.90	2.16	2.16	2.03	2.54	2.03	2.41	2.16	2.41	2.03	2.28	1.90	2.28	0.89	0.76	0.51	0.76	0.51	0.76	0.89
55	EF1ACPU55	1.78	2.03	2.03	1.90	2.41	1.90	2.28	2.03	2.28	1.90	2.16	1.78	2.16	0.76	0.64	0.38	0.64	0.38	0.64	0.76
56	EF1ACPI01	0.25	0.51	0.51	0.38	0.89	0.38	0.76	0.51	0.76	0.38	0.76	0.51	0.64	1.65	1.90	1.65	1.90	1.65	1.65	1.78
57	EF1ACPI02	0.38	0.64	0.64	0.51	1.02	0.51	0.89	0.64	0.89	0.51	0.89	0.64	0.76	1.78	2.03	1.78	2.03	1.78	1.78	1.90
58	EF1ACPI03	0.38	0.64	0.64	0.51	1.02	0.51	0.89	0.64	0.89	0.51	0.89	0.64	0.76	1.78	2.03	1.78	2.03	1.78	1.78	1.90
59	EF1ACPI04	0.51	0.76	0.76	0.64	1.14	0.64	1.02	0.76	1.02	0.64	1.02	0.76	0.89	2.16	2.41	2.16	2.41	2.16	2.16	2.28

60	EF1ACPI05	0.25	0.51	0.51	0.38	0.89	0.38	0.76	0.51	0.76	0.38	0.76	0.51	0.64	1.90	2.16	1.90	2.16	1.90	1.90	2.03
61	EF1ACPI06	0.51	0.76	0.76	0.64	1.14	0.64	1.02	0.76	1.02	0.64	0.76	0.76	0.89	2.16	2.41	2.16	2.41	2.16	2.16	2.28
62	EF1ACPI07	0.25	0.51	0.51	0.38	0.89	0.38	0.76	0.51	0.76	0.38	0.76	0.51	0.64	1.90	2.16	1.90	2.16	1.90	1.90	2.03
63	EF1ACPI08	0.38	0.64	0.64	0.51	1.02	0.51	0.89	0.64	0.89	0.51	0.89	0.64	0.76	2.03	2.28	2.03	2.28	2.03	2.03	2.16
64	EF1ACPI09	0.25	0.51	0.51	0.38	0.89	0.38	0.76	0.51	0.76	0.38	0.76	0.51	0.64	1.90	2.16	1.90	2.16	1.90	1.90	2.03
65	EF1ACPI10	0.51	0.76	0.76	0.64	1.14	0.64	1.02	0.76	1.02	0.64	1.02	0.76	0.89	2.16	2.41	2.16	2.41	2.16	2.16	2.28
66	EF1ACPI11	1.40	1.65	1.65	1.52	2.03	1.52	1.90	1.65	1.90	1.52	1.78	1.65	1.78	1.40	1.52	1.27	1.52	1.27	1.27	1.40
67	EF1ACPI12	0.13	0.38	0.38	0.25	0.76	0.25	0.64	0.38	0.64	0.25	0.64	0.38	0.51	1.78	2.03	1.78	2.03	1.78	1.78	1.90
68	EF1ACPI13	0.25	0.51	0.51	0.38	0.89	0.38	0.76	0.51	0.76	0.38	0.76	0.51	0.64	1.90	2.16	1.90	2.16	1.90	1.90	2.03
69	EF1ACPI14	0.25	0.51	0.51	0.38	0.89	0.38	0.76	0.51	0.76	0.38	0.76	0.51	0.64	1.90	2.16	1.90	2.16	1.90	1.90	2.03
70	EF1ACPI15	0.38	0.64	0.64	0.51	1.02	0.51	0.89	0.64	0.89	0.51	0.89	0.64	0.76	2.03	2.28	2.03	2.28	2.03	2.03	2.16
71	EF1ACPI16	0.38	0.64	0.64	0.51	1.02	0.51	0.89	0.64	0.89	0.51	0.89	0.64	0.76	1.90	2.03	1.78	2.03	1.78	1.78	1.90
72	EF1ACPI17	0.89	1.14	1.14	1.02	1.52	1.02	1.40	1.14	1.40	1.02	1.40	1.14	1.27	2.54	2.79	2.54	2.79	2.54	2.54	2.67
73	EF1ACPI18	4.70	4.95	4.95	4.82	5.33	4.82	5.20	4.95	5.20	4.82	5.20	4.95	5.08	5.46	5.84	5.58	5.84	5.58	5.33	5.71
74	EF1ACPI19	0.25	0.51	0.51	0.38	0.89	0.38	0.76	0.51	0.76	0.38	0.76	0.51	0.64	1.90	2.16	1.90	2.16	1.90	1.90	2.03
75	EF1ACPI20	0.64	0.89	0.89	0.76	1.02	0.76	1.14	0.89	1.14	0.76	1.14	0.89	1.02	2.28	2.54	2.28	2.54	2.28	2.28	2.41
76	EF1ACPI21	0.25	0.51	0.51	0.38	0.89	0.38	0.76	0.51	0.76	0.38	0.76	0.51	0.64	1.90	2.16	1.90	2.16	1.90	1.90	2.03
77	EF1ACPI22	1.02	1.27	1.27	1.14	1.65	1.14	1.52	1.27	1.52	1.14	1.52	1.27	1.40	2.41	2.67	2.41	2.67	2.41	2.41	2.54
78	EF1ACPI23	0.76	1.02	1.02	0.89	1.40	0.89	1.27	1.02	1.27	0.89	1.27	1.02	1.14	2.16	2.41	2.16	2.41	2.16	2.16	2.28
79	EF1ACPI24	0.25	0.51	0.51	0.38	0.89	0.38	0.76	0.51	0.76	0.38	0.76	0.38	0.64	1.90	2.16	1.90	2.16	1.90	1.90	2.03
80	EF1ACPI25	0.25	0.51	0.51	0.38	0.89	0.38	0.76	0.51	0.76	0.38	0.76	0.51	0.64	1.90	2.16	1.90	2.16	1.90	1.90	2.03
81	EF1ACPI26	0.89	1.14	1.14	1.02	1.52	1.02	1.40	1.14	1.40	1.02	1.40	1.14	1.27	2.54	2.79	2.54	2.79	2.54	2.54	2.67
82	EF1ACPI27	0.64	0.89	0.89	0.76	1.27	0.76	1.14	0.89	1.14	0.76	1.14	0.89	1.02	2.28	2.54	2.28	2.54	2.28	2.28	2.41
83	EF1ACPI28	0.25	0.51	0.51	0.38	0.89	0.38	0.76	0.51	0.76	0.38	0.76	0.51	0.64	1.90	2.16	1.90	2.16	1.90	1.90	2.03
84	EF1ACPI29	0.25	0.51	0.51	0.38	0.89	0.38	0.76	0.51	0.76	0.38	0.76	0.51	0.64	1.90	2.16	1.90	2.16	1.90	1.90	2.03
85	EF1ACPI30	0.76	1.02	1.02	0.89	1.40	0.89	1.27	1.02	1.27	0.89	1.27	1.02	1.14	2.16	2.41	2.16	2.41	2.16	2.16	2.28
86	EF1ACPI31	0.51	0.76	0.76	0.64	1.14	0.64	1.02	0.76	1.02	0.64	1.02	0.76	0.89	1.90	2.16	1.90	2.16	1.90	1.90	2.03
87	EF1ACPI32	0.38	0.64	0.64	0.51	1.02	0.51	0.89	0.64	0.89	0.51	0.89	0.64	0.76	2.03	2.28	2.03	2.28	2.03	2.03	2.16
88	EF1ACPI33	0.25	0.51	0.51	0.38	0.89	0.38	0.76	0.51	0.76	0.38	0.76	0.51	0.64	1.90	2.16	1.90	2.16	1.90	1.90	2.03
89	EF1ACPI34	0.38	0.64	0.64	0.51	1.02	0.51	0.89	0.64	0.89	0.51	0.89	0.64	0.76	2.03	2.28	2.03	2.28	2.03	2.03	2.16
90	EF1ACPI35	0.38	0.64	0.64	0.51	1.02	0.51	0.89	0.64	0.89	0.51	0.89	0.64	0.76	2.03	2.28	2.03	2.28	2.03	2.03	2.16
91	EF1ACPI36	-	0.51	0.51	0.38	0.89	0.38	0.76	0.51	0.76	0.38	0.76	0.51	0.64	1.90	2.16	1.90	2.16	1.90	1.90	2.03
92	EF1ACPI37	4	-	0.76	0.64	1.14	0.64	1.02	0.76	1.02	0.64	1.02	0.76	0.89	2.16	2.41	2.16	2.41	2.16	2.16	2.28
93	EF1ACPI38	4	6	-	0.64	0.89	0.38	0.76	0.76	1.02	0.64	1.02	0.76	0.76	2.16	2.41	2.16	2.41	2.16	2.16	2.28
94	EF1ACPI39	3	5	5	-	1.02	0.51	0.89	0.64	0.89	0.51	0.89	0.64	0.76	2.03	2.28	2.03	2.28	2.03	2.03	2.16
95	EF1ACPI40	7	9	7	8	-	0.76	1.14	1.14	1.40	1.02	1.40	1.14	1.27	2.54	2.79	2.54	2.79	2.41	2.54	2.67
96	EF1ACPI41	3	5	3	4	6	-	0.64	0.64	0.89	0.51	0.89	0.64	0.76	2.03	2.28	2.03	2.28	2.03	2.03	2.16
97	EF1ACPI42	6	8	6	7	9	5	-	1.02	1.27	0.89	1.27	1.02	1.14	2.41	2.67	2.41	2.67	2.41	2.41	2.54
98	PI1	4	6	6	5	9	5	8	-	1.02	0.64	1.02	0.76	0.89	2.16	2.41	2.16	2.41	2.16	1.90	2.03
99	PI2	6	8	8	7	11	7	10	8	-	0.89	1.27	1.02	1.14	2.41	2.54	2.41	2.67	2.41	2.41	2.54
100	PI3	3	5	5	4	8	4	7	5	7	-	0.89	0.64	0.76	2.03	2.28	2.03	2.28	2.03	2.03	2.16
101	PI4	6	8	8	7	11	7	10	8	10	7	-	1.02	1.14	2.284	2.54	2.284	2.54	2.28	2.28	2.41
102	PI5	4	6	6	5	9	5	8	6	8	5	8	-	0.89	1.90	2.157	1.90	2.16	1.90	1.90	2.03
103	PI6	5	7	6	6	10	6	9	7	9	6	9	7	-	2.28	2.54	2.28	2.54	2.28	2.28	2.41
104	PU1	15	17	17	16	20	16	19	17	19	16	18	15	18	-	1.14	0.89	1.14	0.89	0.89	1.02
105	PU2	17	19	19	18	22	18	21	19	20	18	20	17	20	9	-	0.76	1.02	0.76	1.02	1.14
106	PU3	15	17	17	16	20	16	19	17	19	16	18	15	18	7	6	-	0.76	0.51	0.76	0.89
107	PU4	17	19	19	18	22	18	21	19	21	18	20	17	20	9	8	6	-	0.76	1.02	1.14
108	PU5	15	17	17	16	19	16	19	17	19	16	18	15	18	7	6	4	6	-	0.76	0.89
109	PU6	15	17	17	16	20	16	19	15	19	16	18	15	18	7	8	6	8	6	-	0.89
110	PU7	16	18	18	17	21	17	20	16	20	17	19	16	19	8	9	7	9	7	7	-

Numbers above the diagonal indicate percent distance values; numbers below the diagonal indicate absolute distance values. *C. punctiferalis*, EF1ACPU01–EF1ACPU55 and PU1–PU7; *C. pinicolalis*, EF1ACPI01–EF1ACPI42 and PI1–PI6.
