# Supplementary material for: Morphological, Ecological, and Molecular Divergence of Conogethes pinicolalis from C. punctiferalis (Lepidoptera: Crambidae)
Source: Insects. 2021 May 15;12(5):455. doi: 10.3390/insects12050455 (PMC8156953; doi:10.3390/insects12050455)
Supplement: Supplementary file 1 [file insects-12-00455-s001.zip › Supplementary References.pdf]

Supplementary References

1. Wu, Q.L.; Gong, Y.J.; Shi, B.C.; Gu, Y.; Wei, S.J. The complete mitochondrial genome of the yellow peach moth *Dichocrocis punctiferalis* (Lepidoptera: Pyralidae). *Mitochondrial DNA* **2013**, *24*, 105–107.
2. Ashfaq, M.; Akhtar, S.; Rafi, M.A.; Mansoor, S.; Hebert, P.D.N. Mapping global biodiversity connections with DNA barcodes: Lepidoptera of Pakistan. *PLoS ONE* **2017**, *12*, e0174749, doi:10.1371/journal.pone.0174749.
3. Sam, K.; Ctvrtecka, R.; Miller, S.E.; Rosati, M.E.; Molem, K.; Damas, K.; Gewa, B.; Novotny, V. Low host specificity and abundance of frugivorous lepidoptera in the lowland rain forests of Papua New Guinea. *PLoS ONE* **2017**, *12*, e0171843.
4. Hebert, P.D.N.; deWaard, J.R.; Zakharov, E.V.; Prosser, S.W.J.; Sones, J.E.; McKeown, J.T.A.; Mantle, B.; Salle, J.L. A DNA 'Barcode Blitz': Rapid digitization and sequencing of a natural history collection. *PLoS ONE* **2013**, *8*, e68535.
5. Mally, R.; Hayden, J.E.; Neinhuis, C.; Jordal, B.H.; Nuss, M. The phylogenetic systematics of Spilomelinae and Pyraustinae (Lepidoptera: Pyraloidea: Crambidae) inferred from DNA and morphology. *Arthropod Syst. Phylogeny* **2019**, *77*, 141–204, doi:10.26049/asp77-1-2019-07.
