# Supplementary figures and images for: Morphological, Ecological, and Molecular Divergence of Conogethes pinicolalis from C. punctiferalis (Lepidoptera: Crambidae)
Source: Insects. 2021 May 15;12(5):455. doi: 10.3390/insects12050455 (PMC8156953; doi:10.3390/insects12050455)

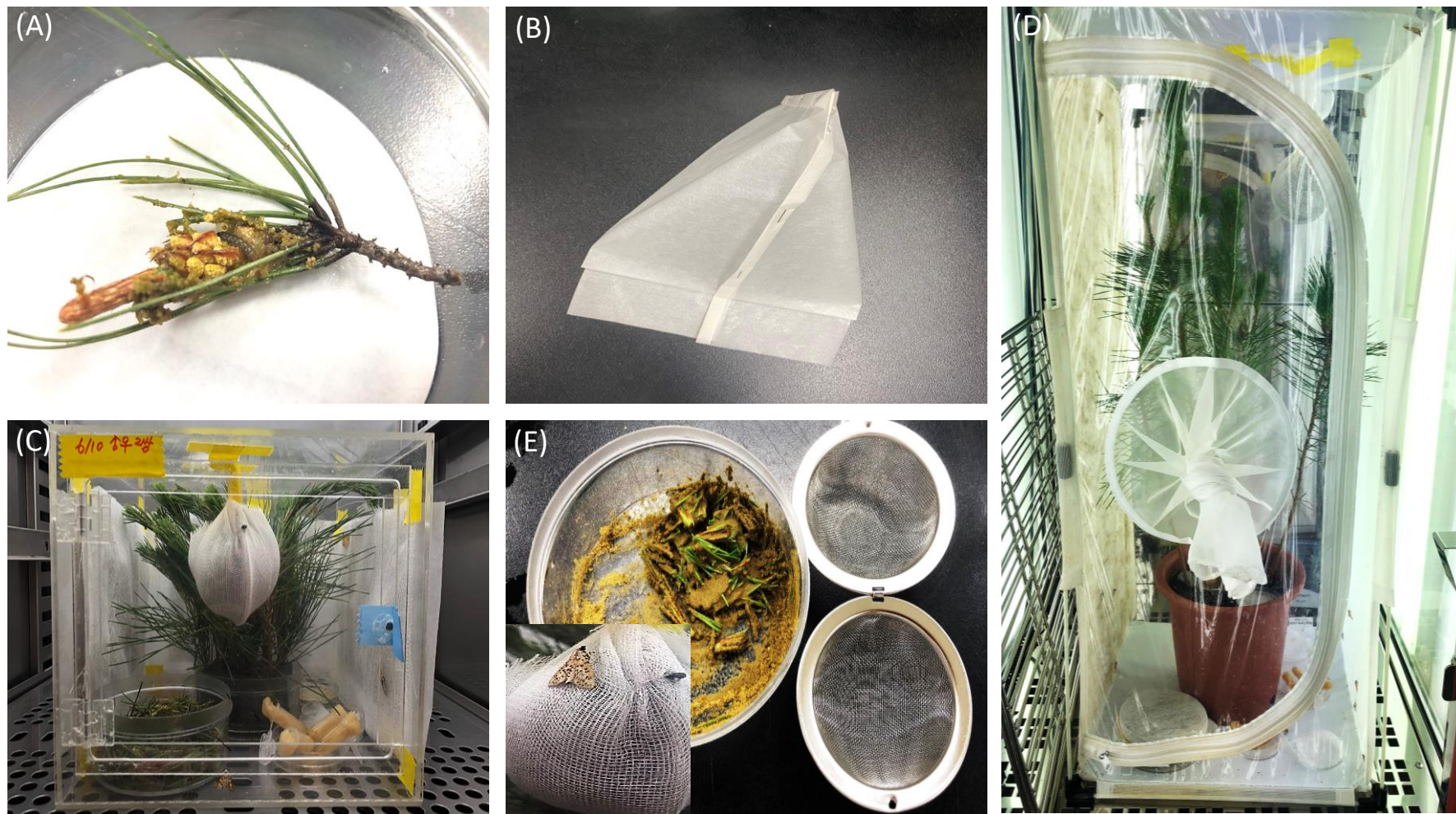

**Figure S1.**

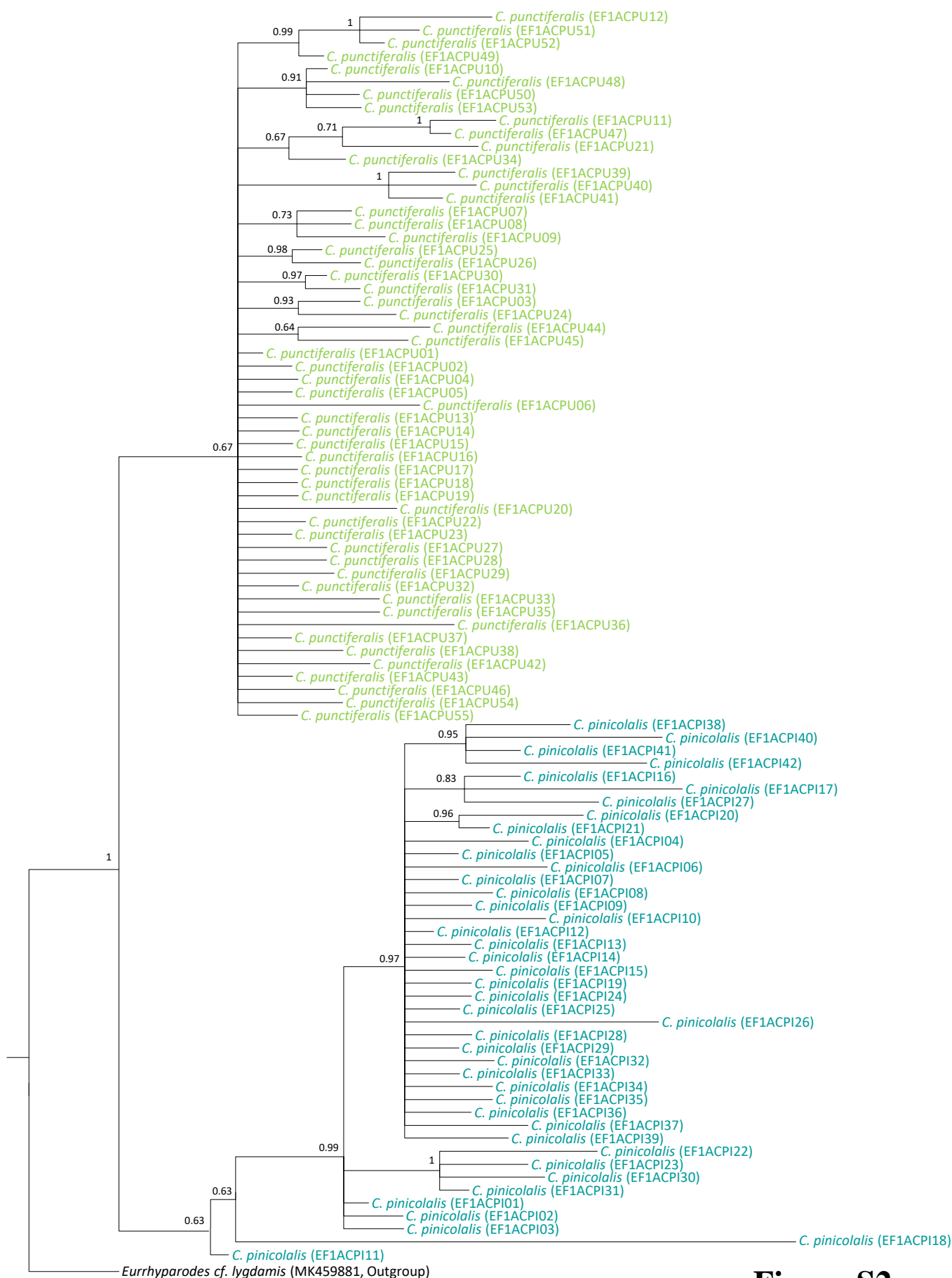

**Figure S2.**

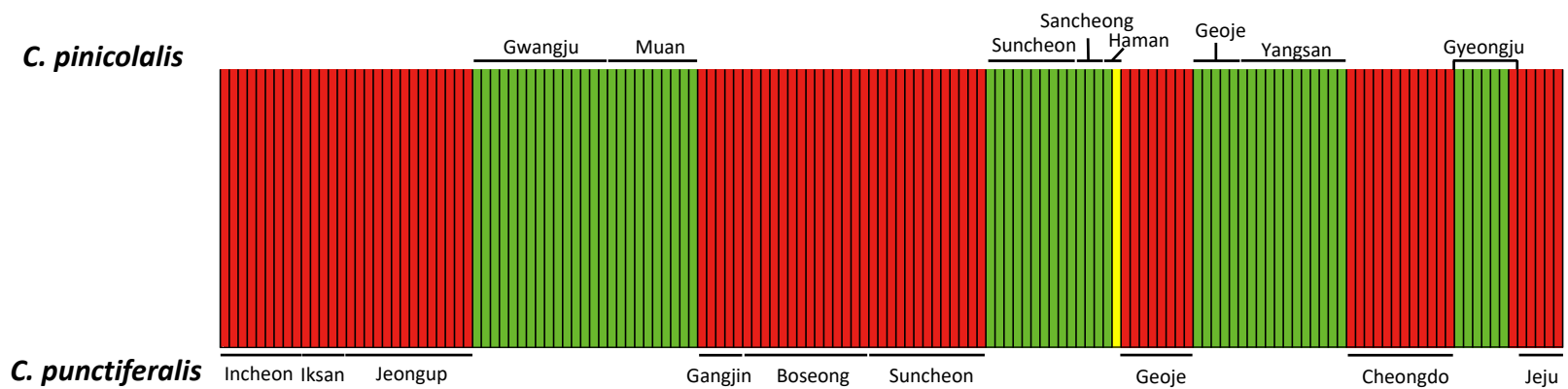

**Figure S3.**

Supplement: Supplementary file 1 [file insects-12-00455-s001.zip › All Figure S1-S3.pdf]
